# How metallylenes activate small molecules†

**DOI:** 10.1039/d0sc05987k

**Published:** 2021-02-18

**Authors:** Pascal Vermeeren, Michael T. Doppert, F. Matthias Bickelhaupt, Trevor A. Hamlin

**Affiliations:** Department of Theoretical Chemistry, Amsterdam Institute of Molecular and Life Sciences (AIMMS), Amsterdam Center for Multiscale Modeling (ACMM), Vrije Universiteit Amsterdam De Boelelaan 1083 1081 HV Amsterdam The Netherlands t.a.hamlin@vu.nl; Institute for Molecules and Materials (IMM), Radboud University Heyendaalseweg 135 6525 AJ Nijmegen The Netherlands

## Abstract

We have studied the activation of dihydrogen by metallylenes using relativistic density functional theory (DFT). Our detailed activation strain and Kohn–Sham molecular orbital analyses have quantified the physical factors behind the decreased reactivity of the metallylene on going down Group 14, from carbenes to stannylenes. Along this series, the reactivity decreases due to a worsening of the back-donation interaction between the filled lone-pair orbital of the metallylene and the σ*-orbital of H_2_, which, therefore, reduces the metallylene–substrate interaction and increases the reaction barrier. As the metallylene ligand is varied from nitrogen to phosphorus to arsenic a significant rate enhancement is observed for the activation of H_2_ due to (i) a reduced steric (Pauli) repulsion between the metallylene and the substrate; and (ii) less activation strain, as the metallylene becomes increasingly more predistorted. Using a rationally designed metallylene with an optimal Group 14 atom and ligand combination, we show that a number of small molecules (*i.e.* HCN, CO_2_, H_2_, NH_3_) may also be readily activated. For the first time, we show the ability of our H_2_ activated designer metallylenes to hydrogenate unsaturated hydrocarbons. The results presented herein will serve as a guide for the rational design of metallylenes toward the activation of small molecules and subsequent reactions.

## Abstract 2

Quantum chemical analyses reveal how model metallylene catalysts activate H_2_. This is the first step towards the rational design of metallylenes for the activation of small molecules and subsequent reactions.

## Graphical abstract

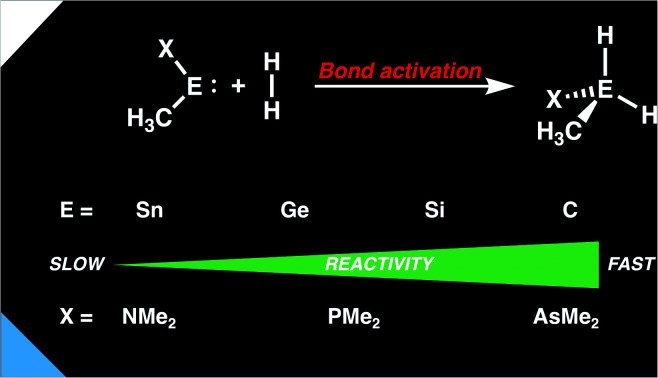

## Introduction

Originating with the seminal work of Philip P. Power in 2010,^1^ the activation of small molecules by main-group elements, a field traditionally dominated by transition metal chemistry, has fascinated chemists. A class of main-group species that has received recent attention are carbenes and their heavier Group 14 analogs (metallylenes).^2^ Owing to their large singlet–triplet energy gap, metallylenes have an sp^2^-hybridized lone pair orbital in the plane of the molecule and a vacant p-type orbital perpendicular to the molecular plane, which resemble the filled and empty nd and ns orbitals found in transition metal catalysts.^1^ As a result, these molecular species are able to participate in similar chemistry as their transition metal analogs.^3^

Recently, Group 14 metallylenes have been shown to activate a number of small molecules, such as H_2_, by oxidative insertion into the respective bond of the molecule.^4–7^ The reactivity of these metallylenes is commonly ascribed to the HOMO–LUMO gap of this species. It has been postulated that metallylenes possessing a small HOMO–LUMO gap are more active towards bond activation since a correlation has been found between the magnitude of the metallylene's band gap and the height of the reaction barrier corresponding to the activation of a chemical bond.^4,6*b*,6*c*^ The energies of the HOMO and LUMO of these metallylenes, and hence their activity in small molecule activation, can be tuned by (i) narrowing the angle between the ligands around the central Group 14 metallylene atom; and (ii) changing the nature of the ligands.^2*a*^ On the contrary, Ess *et al.* showed, by applying an energy decomposition analyses based on absolutely localized molecular orbitals (ALMO-EDA) on transition state structures, that the reaction barrier height for the activation of H_2_ by metallylenes, as well as the differences in reaction barriers between carbenes, silylenes, and germylenes, arise from the activation strain accompanied by the stretch of the H–H bond, which, in turn, is controlled by intermolecular electron repulsion.^8^ Furthermore, Ess revealed that carbenes act, in analogy with transition metal catalysts, as amphiphiles towards H_2_ activation, where both the back-donation HOMO_metallylene_–LUMO_H_2__ and donation LUMO_metallylene_–HOMO_H_2__ interaction are in play. Silylenes and germylenes, on the other hand, react as nucleophiles and hence predominantly feature a back-donation HOMO_metallylene_–LUMO_H_2__ interaction.

**Scheme 1:**
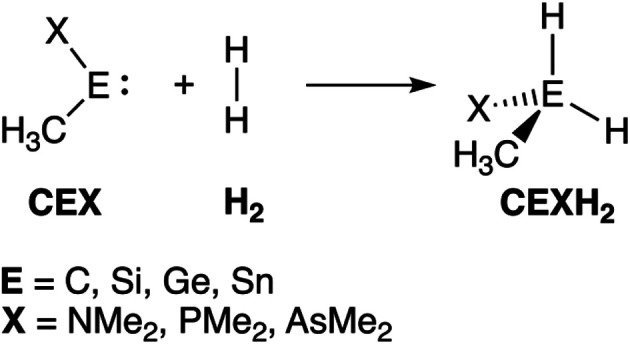
The activation of H_2_ by a singlet metallylene (**CEX**; where E = C, Si, Ge, Sn, and X = NMe_2_, PMe_2_, AsMe_2_).

Herein, we have performed a systematic computational study on the activation of dihydrogen by various metallylenes using relativistic density functional theory (DFT) at ZORA-BP86/TZ2P level,^9–11^ as implemented in the Amsterdam Density Functional (ADF) program.^12^ To this end, we have selected the model singlet metallylene H_3_C–E–X (**CEX**), where E = C, Si, Ge, Sn; and X = NMe_2_, PMe_2_, AsMe_2_ (see Scheme 1), as it resembles the metalyllenes previously used in both experiment and theory.^4*a*,6*b*,8^ The effect of varying the Group 14 central atom E, as well as changing the Group 15 ligand X, on the activation of H_2_ has been analyzed using the activation strain model (ASM)^13^ of reactivity in combination with quantitative Kohn–Sham molecular orbital (KS-MO) theory and a matching canonical energy decomposition analysis (EDA) scheme.^14^ In addition, we show that the rationale found behind the trends in reactivity of our model systems can be extrapolated to explain the reactivity trends in experimentally used metallylenes. Furthermore, we highlight the applicability of the H_2_ activated metallylene species to efficiently hydrogenate unsaturated bonds.

## Results and discussion

### Reaction profiles

Table 1 summarizes the reaction barriers, Δ*E*^‡^, and reaction energies, Δ*E*_rxn_, of the activation of H_2_ by various singlet metallylenes with varying Group 15 ligands, denoted as **CEX**, where E = C, Si, Ge, Sn, and X = NMe_2_, PMe_2_, AsMe_2_ (see Fig. S1† in the ESI for transition state structures). Two distinct trends can be observed. In the first place, varying the central atom E of **CEX** by going down Group 14, *i.e.*, from C to Sn, while keeping the ligand X consistent increases the reaction barrier and makes the reaction less exergonic. An *exception* is the series with the AsMe_2_ ligand. In this series, the reaction barrier slightly lowers from carbon to silicon and increases again from silicon to germanium to tin. The reaction energy along this series, however, becomes steadily less exergonic. Secondly, variation of the Group 15 ligand X from NMe_2_ to PMe_2_ to AsMe_2_ while keeping the central atom E constant stabilizes, for all studied metallylenes, the reaction barrier significantly and makes the reaction more exergonic. The computed trends in reactivity at ZORA-BP86/TZ2P agree well with those calculated in apolar and polar solvent (COSMO(toluene) and COSMO(water)),^15^ the meta-hybrid and dispersion-corrected exchange-correlation functionals ZORA-M06-2X^16^/TZ2P//ZORA-BP86/TZ2P, ZORA-BP86-D3(BJ)^17^/TZ2P, and ZORA-M06-2X/TZ2P//ZORA-BP86-D3(BJ)/TZ2P, and the more accurate DLPNO-CCSD(T)/def2-QZVPP//ZORA-BP86/TZ2P (Tables S1–S6†). Statistical analyses revealed that ZORA-BP86/TZ2P performs equally as well as ZORA-M06-2X/TZ2P//ZORA-BP86/TZ2P and slightly better than ZORA-BP86-D3(BJ)/TZ2P and ZORA-M06-2X/TZ2P//ZORA-BP86-D3(BJ)/TZ2P relative to the DLPNO-CCSD(T)^18^/def2-QZVPP^19^//ZORA-BP86/TZ2P data (see Table S7†).

**Table 1:** Electronic reaction barriers (Δ*E*^‡^) and reaction energies (Δ*E*_rxn_) (in kcal mol^−1^) of the activation of H_2_ by H_3_C–E–X (**CEX**) metallylenes^a^

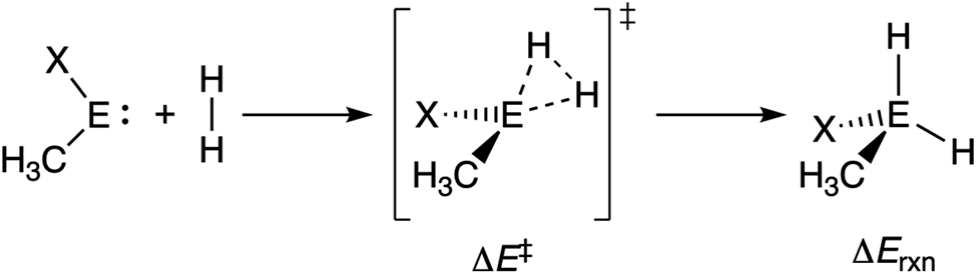			
E	X	Δ*E*^‡^^b^	Δ*E*_rxn_^b^
C	NMe_2_	12.2 (21.3)	−58.3 (−41.8)
C	PMe_2_	8.3 (17.6)	−80.1 (−63.2)
C	AsMe_2_	5.5 (14.7)	−95.6 (−78.7)
Si	NMe_2_	23.4 (31.6)	−35.8 (−25.1)
Si	PMe_2_	8.5 (18.6)	−44.5 (−32.4)
Si	AsMe_2_	2.4 (12.5)	−50.0 (−37.8)
Ge	NMe_2_	38.0 (45.8)	−12.9 (−2.6)
Ge	PMe_2_	18.4 (28.3)	−26.2 (−14.3)
Ge	AsMe_2_	13.1 (22.5)	−30.7 (−19.1)
Sn	NMe_2_	48.6 (55.8)	1.5 (10.1)
Sn	PMe_2_	29.0 (38.0)	−12.1 (1.7)
Sn	AsMe_2_	25.0 (33.5)	−15.2 (−5.4)

a Computed at ZORA-BP86/TZ2P.

b Gibbs free energies are given in parenthesis.

### Variation along group 14 metallylene central atom

**Fig. 1:**
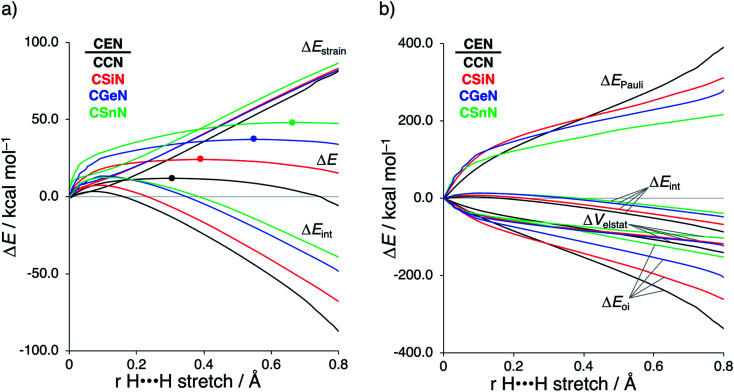
(a) Activation strain analysis and (b) energy decomposition analysis of the H_2_ bond activation by metallylenes **CEN** with variating Group 14 central atom (E = C, Si, Ge, Sn), where the transition states are indicated with a dot and the energies are projected onto the H⋯H bond stretch of H_2_, computed at ZORA-BP86/TZ2P.

Next, we turn to the activation strain model (ASM)^13^ of reactivity to gain quantitative insight into the physical factors leading to the changes in reactivity upon varying the metallylene central atom and the ligand. This model involves decomposing the electronic energy (Δ*E*) into two distinct energy terms, namely, the strain energy (Δ*E*_strain_) that results from the deformation of the individual reactants and the interaction energy (Δ*E*_int_) between the deformed reactants along the reaction coordinate, defined, in this case, by the stretch of the activated H–H bond. This critical reaction coordinate undergoes a well-defined change throughout the reaction and has successfully been used in the past for the analysis of similar reactions.^20^ First, we focus on the effect of changing the central Group 14 atom E on the activation of H_2_. In Fig. 1, we show the activation strain diagram of the **CEN** series for which the effects are the largest. Note that the activation strain diagrams of all other **CEX** series (**CEP** and **CEAs**, respectively) possess the same, only less pronounced characteristics (Fig. S2 and S3†). The increase of the reaction barrier on going from **CCN** to **CSnN** is mainly dictated by a consistently less stabilizing interaction energy. In other words, the reaction with **CCN** goes, along the entire reaction coordinate, with the most stabilizing interaction energy and hence the lowest reaction barrier.^21^ The reaction with **CSnN**, on the contrary, experiences the least stabilizing interaction energy and, therefore, the highest reaction barrier. On top of that, the former reaction also encounters the least destabilizing strain energy, which again lowers the reaction barrier. The important role of the interaction energy on the observed reactivity trend prompted the analysis of the different contributors to the interaction energy using the canonical energy decomposition analysis (EDA).^14^ Our canonical EDA decomposed the Δ*E*_int_ between the reactants into three physically meaningful energy terms: classical electrostatic interaction (Δ*V*_elstat_), (steric) Pauli repulsion (Δ*E*_Pauli_) which, in general, arises from the two-center four-electron repulsion between the closed-shell orbitals of both reactants, and stabilizing orbital interactions (Δ*E*_oi_) that account, among others, for HOMO–LUMO interactions. By performing the EDA, we establish that the trend in Δ*E*_int_ is predominantly determined by the orbital interactions, Δ*E*_oi_, which are, in analogy with the trend in Δ*E*_int_, the most stabilizing for **CCN** and the least for **CSnN** (see Table S12† for analysis at consistent geometry). The Pauli repulsion, Δ*E*_Pauli_, and electrostatic interaction, Δ*V*_elstat_, on the other hand, have a small or even opposite effect (Δ*E*_Pauli_ is more destabilizing for **CCN**) on the interaction energy.^21^

**Fig. 2:**
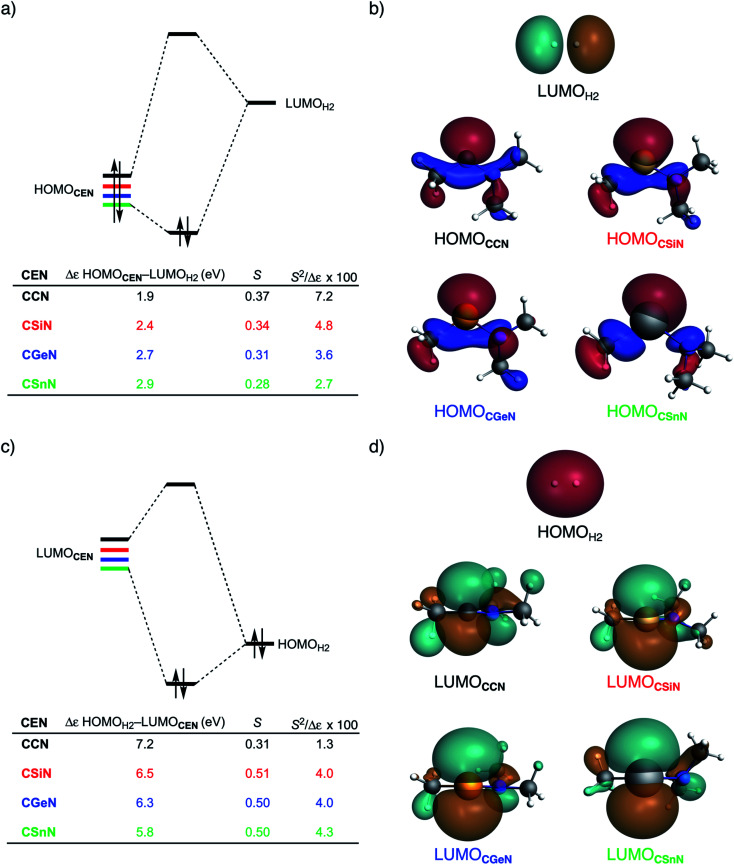
(a) Molecular orbital diagram with the key orbital energy gap and overlap of the HOMO**CEN**–LUMO_H_2__ back-donation interaction, (b) key orbitals contributing to the back-donation interaction (isovalue = 0.03 Bohr^−3/2^), (c) Molecular orbital diagram with the key orbital energy gap and overlap of the HOMO_H_2__–LUMO**CEN** donation interaction, and (d) key orbitals contributing to the donation interaction (isovalue = 0.03 Bohr^−3/2^) of the H_2_ bond activation by **CEN** metallylenes with variating Group 14 central atom (E = C, Si, Ge, Sn) computed using ZORA-BP86/TZ2P on consistent geometries with a H⋯H bond stretch of 0.47 Å.

To further probe the key orbital interactions involved in the H_2_ activation by metallylenes, we analyze the orbitals participating in these interactions using a Kohn–Sham molecular orbital analysis on consistent geometries with a H⋯H bond stretch of 0.47 Å at ZORA-BP86/TZ2P.^14*b*,22,23^ The consistent geometry of a H⋯H bond stretch of 0.47 Å was judiciously selected because it provided transition state-like geometries with energies that differ no more than 2 kcal mol^−1^ compared to the respective transition state. Analysis at this point on the reaction coordinate (near all transition states), rather than the transition state alone, ensured that the results are not skewed by the position of the transition state (*i.e.*, early- or late-transition state).^13*c*^ In contrast with the work of Ess *et al.*,^8^ we find that two major orbital interaction mechanisms are playing a role in all bond activation reactions, namely, the back-donation interaction, where the lone pair orbital of the metallylene (HOMO**CEN**) donates electrons into the σ*-orbital of H_2_ (LUMO_H_2__) (Fig. 2a and b), and the donation interaction, where the empty p-type orbital on the central atom E of the metallylene (LUMO**CEN**) accepts electrons from the σ-orbital of H_2_ (HOMO_H_2__) (Fig. 2c and d).

**Fig. 3:**
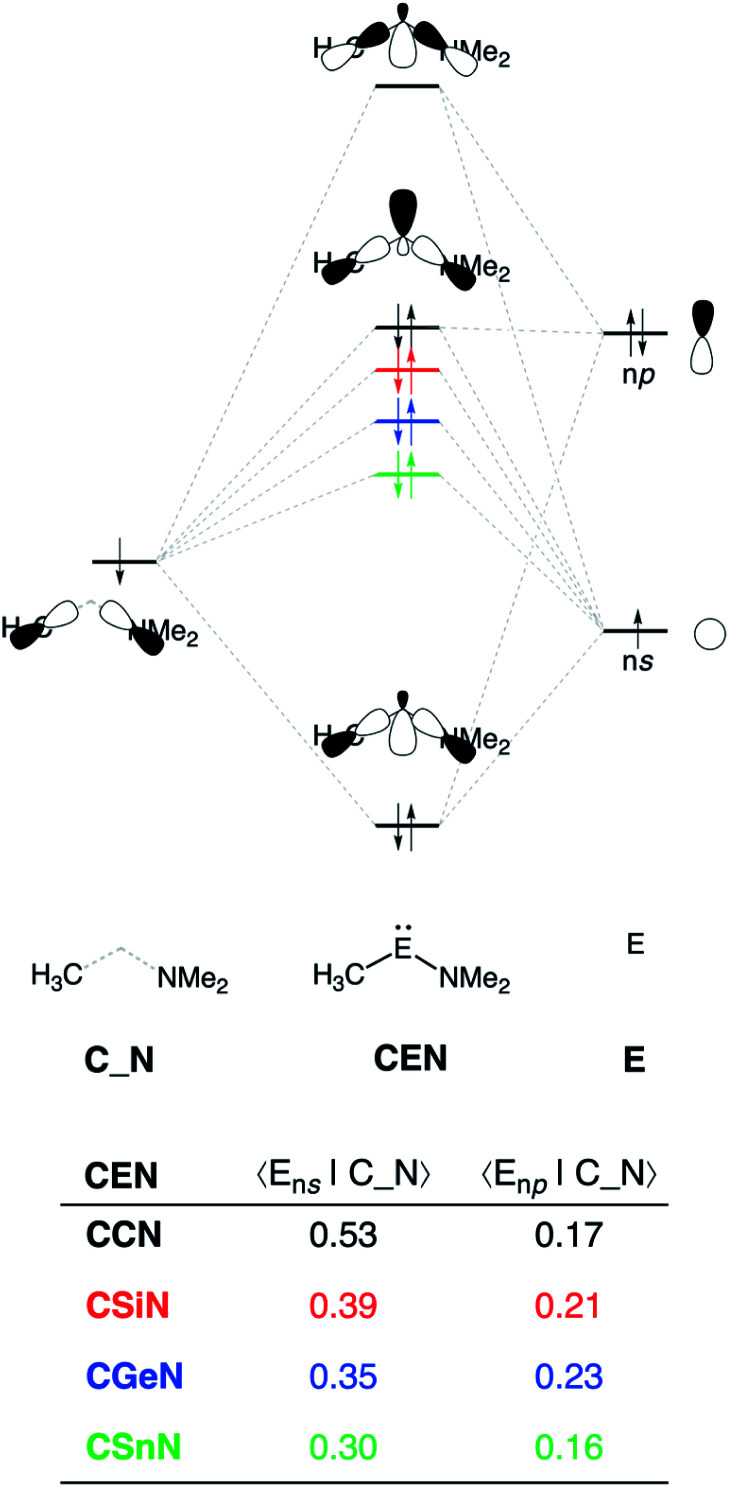
Schematic representation of the construction of the **CEN** HOMO (E = C, Si, Ge, Sn) and orbital overlap between the atomic orbitals of E (E_ns_ and E_np_) and the in-phase ligand orbital (C_N), computed using ZORA-BP86/TZ2P level at consistent geometries with a H⋯H bond stretch of 0.47 Å.

The reduction in stabilizing Δ*E*_oi_ (and thus the increasing reaction barrier), when going from **CCN** to **CSnN**, can be ascribed to the weakening of the back-donation interaction. Along this series, the HOMO**CEN** goes down in energy and becomes more diffuse, *i.e.*, increased spatial extent of the lone pair orbital on E, which leads to a less favorable (larger) HOMO–LUMO gap and a poorer orbital overlap. For the back-donation interaction (Fig. 2a and b), **CCN**, the most reactive metallylene, has the smallest HOMO**CEN**–LUMO_H_2__ orbital energy gap (1.9 eV) and the largest orbital overlap (*S* = 0.37). As we go down Group 14, the HOMO**CEN**–LUMO_H_2__ orbital energy gap increases from 1.9 eV for **CCN** to 2.9 eV for **CSnN**, due to a more stable **CEN** HOMO. In order to understand why the HOMO**CEN** lowers in energy (*i.e.*, stabilizes) when descending in Group 14, we perform an additional Kohn–Sham molecular orbital analysis where the construction of the HOMO**CEN** from the interaction between the ns atomic orbital (AO) of E and the in-phase C_N ligand orbitals is examined (see Fig. 3). Note that the in-phase C_N orbital is the σ-orbital in the **CEN** plane responsible for the formation of the C–E and E–N bonds. We found that the stabilization of the HOMO**CEN**, going down in Group 14, is caused by the reduced antibonding character originating from the interaction between the ns AO of E and the in-phase C_N ligand orbitals. The **CEN** HOMO arises from both the antibonding interaction between the ns AO of E and the C_N ligand orbital, which destabilizes the **CEN** HOMO, and the bonding interaction between the np AO of E and the C_N ligand orbital, which stabilizes the **CEN** HOMO. The antibonding E_ns_–C_N interaction becomes, going from **CCN** to **CSnN**, consistently weaker, as the overlap reduces from 〈C_2s_|C_N〉 = 0.53 to 〈Sn_5s_|C_N〉 = 0.30. As a result, the **CEN** HOMO experiences less antibonding character, and hence lowers in energy (stabilizes). Additionally, along this series, the bonding E_np_–C_N interaction becomes more pronounced, which also contributes to the stabilization of the **CEN** HOMO. Note that the 〈Sn_5p_|C_N〉 orbital overlap is slightly less compared to the other Group 14 analogs, due to the diffuseness of the Sn 5p orbital that extends past the nodal surface of the C_N ligand orbital and, therefore, reduces the orbital overlap. Besides an increased HOMO**CEN**–LUMO_H_2__ energy gap, there is also a continuous decrease in orbital overlap upon going from **CCN** to **CSnN**. Along this series, the increasing diffuseness of the HOMO**CEN**, as the AOs of E becomes larger, gives rise to a spatial mismatch with the LUMO_H_2__, resulting in a less favorable HOMO**CEN**–LUMO_H_2__ orbital overlap.

The stronger donation interaction between the LUMO**CEN**–HOMO_H_2__ of **CGeN** and **CSnN**, on the other hand, will partly, but not completely, compensate their weaker back-donation interaction compared to **CCN** and **CSiN** (Fig. 2c and d). Despite the fact that almost all orbital overlaps between LUMO**CEN**–HOMO_H_2__ are larger compared to HOMO**CEN**–LUMO_H_2__ (*S* = 0.31 for **CCN**, *S* = 0.51 for **CSiN**, *S* = 0.50 for **CGeN**, and *S* = 0.50 for **CSnN**), the donation orbital interaction mechanism is not able to overrule the trend dictated by the back-donation interaction, because the LUMO**CEN**–HOMO_H_2__ energy gaps, ranging from 7.1 eV for **CCN** to 5.8 eV for **CSnN**, are significantly larger than the HOMO**CEN**–LUMO_H_2__ analogs. Thus, it can be concluded that the strong back-donation orbital interaction of **CCN** induces a significant stabilizing orbital interaction energy, which manifests in a more favorable interaction energy and hence a lower reaction barrier. The back-donation orbital mechanism becomes, going down Group 14, less prominent, resulting in reduced orbital interactions and, as a consequence, a higher reaction barrier.

### Variation along group 15 ligand

**Fig. 4:**
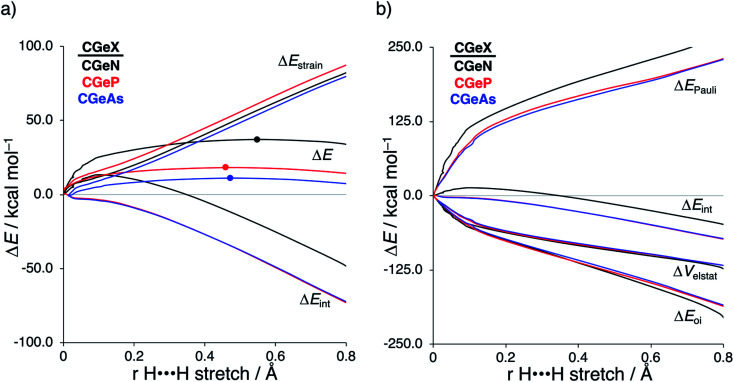
(a) Activation strain analysis and (b) energy decomposition analysis of the H_2_ bond activation by germylenes **CGeX** with variating Group 15 ligands (X = NMe_2_, PMe_2_, AsMe_2_), where the transition states are indicated with a dot and the energies are projected onto the H⋯H bond stretch of H_2_, computed at ZORA-BP86/TZ2P.

After establishing the trends in reactivity upon changing the central Group 14 metallylene atom, we analyze the role of the Group 15 ligand on the reactivity of the metallylene towards the activation of H_2_. Here, we solely discuss the reactivity trend of **CGeX**, which resembles metallylenes used experimentally.^6*b*^ The activation strain diagram of all other metallylenes (**CCX**, **CSiX**, and **CSnX**) show similar characteristics and are shown in Fig. S5–S7 in the ESI.† The activation strain analysis (ASA) provided in Fig. 4a clearly shows that the reaction barriers lower when varying the Group 15 ligand from NMe_2_ to PMe_2_ to AsMe_2_, which is, as we will discuss later, originating from both a reduced Pauli-repulsive orbital overlap between the reactants and less activation strain in the germylene. The high **CGeN** reaction barrier is solely caused by a less stabilizing interaction energy that is even repulsive at an early stage of the reaction. By applying the energy decomposition analysis, we established that the more destabilizing Pauli repulsion between the filled orbitals of **CGeN** and H_2_ is the causal actor behind the less stabilizing interaction energy and hence the higher reaction barrier (Fig. 4b). The electrostatic and orbital interactions are, on the other side, equal or even more stabilizing compared to **CGeP** and **CGeAs**, and, therefore, not decisive for the observed trend in reactivity.^21^ The difference between **CGeP** and **CGeAs** can be ascribed to their difference in strain energy, and this appears to be due to the favorable pre-distortion of the ligand in the latter. The PMe_2_ ligand of **CGeP** is, in the equilibrium geometry of the germylene, trigonal planar, due to a strong hyperconjugation interaction between the empty 4p atomic orbital of germanium and the filled 3p atomic orbital of phosphorus (〈4p_Ge_|3p_P_〉 = 0.27) (Fig. S9†). Along the course of the reaction, however, the phosphorus ligand must pyrimidalize which leads to the loss of the stabilizing hyperconjugation interaction. On the contrary, the arsenic ligand of **CGeAs** is, in the equilibrium geometry of the metallylene, already pyramidal (*i.e.*, favorably predistorted) and, therefore, benefits from a less destabilizing activation strain.

**Fig. 5:**
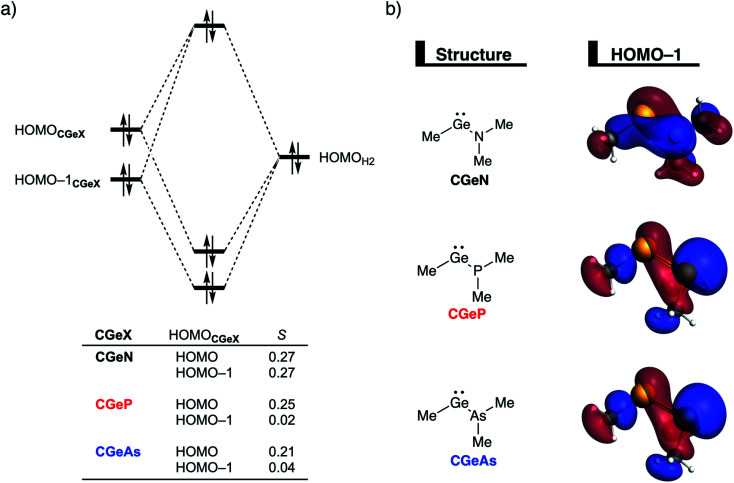
(a) Molecular orbital diagram of the most important occupied–occupied orbital overlap of the H_2_ bond activation by germylenes with various Group 15 ligands (**CGeX**) and (b) key metallylene occupied orbitals (isovalue = 0.03 Bohr^−3/2^) computed at ZORA-BP86/TZ2P on consistent geometries with a H⋯H bond stretch of 0.47 Å.

To understand the origin of the more destabilizing Pauli repulsion for the H_2_ activation using **CGeN** compared to **CGeP** and **CGeAs**, which causes the intrinsic differences in their reactivity, we perform a Kohn–Sham molecular orbital (KS-MO) analysis.^14*b*,22^ The occupied molecular orbitals of **CGeX** and H_2_, that determine the underlying differences in Pauli repulsion, were quantified on consistent geometries with a H⋯H bond stretch of 0.47 Å (Fig. 5a). The most important occupied MOs of **CGeX** involved in the two-center four-electron interaction are the HOMO and HOMO−1, which are the lone pair orbital of the germylene and the hyperconjugation between the empty 4p-type orbital on the central Ge atom and the filled np lone pair orbital of Group 15 ligand. The difference in Pauli repulsion between the reactions with the three different **CGeX** germylenes is predominantly caused by the HOMO−1**CGeX**–HOMO_H_2__ interaction, going from *S* = 0.27 for **CGeN** to *S* = 0.02 and 0.04 for **CGeP** and **CGeAs**, respectively. Interestingly, the interaction between the lone pair orbital of Ge and the filled σ-orbital of H_2_ (HOMO**CGeX**–HOMO_H_2__) is the main responsible factor for the magnitude of the Pauli repulsion but only has a small contribution to the underlying trend in Pauli repulsion between the different germylenes. The large difference in repulsive occupied–occupied orbital overlap can be explained by looking at the pyramidalization of the Group 15 ligand. In their equilibrium geometry, the NMe_2_ and PMe_2_ ligands of **CGeN** and **CGeP**, respectively, are trigonal planar due to hyperconjugation between the empty 4p-type orbital of Ge and the filled np orbital of the Group 15 ligand (*vide supra*; Fig. S7†). To reduce steric repulsion with the incoming H_2_, the Group 15 ligand of **CGeP** and **CGeAs** deforms from trigonal planar to trigonal pyramidal which goes with the loss of hyperconjugation (**CGeP**: 〈4p_Ge_|3p_P_〉 = 0.02, **CGeAs**: 〈4p_Ge_|5p_As_〉 = 0.01; in the consistent geometries used in Fig. 4). This effectively polarizes the HOMO−1**CGeX** away from the central germanium atom and results in a well-defined np lone pair orbital lobe on the Group 15 ligand, for **CGeP** and **CGeAs**, respectively (see blue lobe on the right side of Fig. 5b), which in turn leads to less orbital amplitude pointing towards the incoming H_2_. The Group 15 ligand of the **CGeN** gemylene, on the other hand, deforms only little over the course of the reaction and retains the hyperconjugation interaction (**CGeN**: 〈4p_Ge_|2p_N_〉 = 0.15), which leads to a large HOMO−1**CGeN** orbital amplitude on Ge and, consequently, a larger orbital overlap with H_2_ (Fig. 5b). In summary, the loss of hyperconjugation interaction upon pyramidalization of the Group 15 ligand results in less 〈HOMO−1**CGeX**|HOMO_H_2__〉 overlap and ultimately, to a less destabilizing Pauli repulsion and a lower reaction barrier, for **CGeP** and **CGeAs** compared to **CGeN**.

### Validating the model metallylene species

**Fig. 6:**
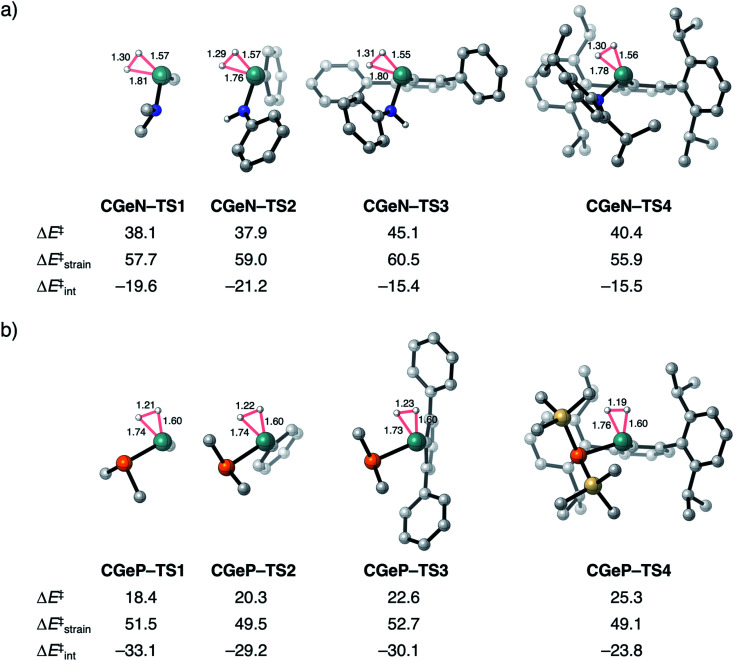
Transition structures with key geometrical information (in Å), computed electronic reaction barriers (Δ*E*^‡^), strain (Δ*E*^‡^_strain_), and interaction energies (Δ*E*^‡^_int_) (in kcal mol^−1^) for the H_2_ activation by the synthesizable germylene species^6*b*^ (a) **CGeN–4** and (b) **CGeP–4** and the, in three steps, simplified analogs, computed at ZORA-BP86/TZ2P. Hydrogen atoms are not shown.

To validate our model metallylenes and check whether our computed reactivity trends are a good representation of the experimentally used metallylenes, we take a retrosynthesis approach and build in the molecular complexity of the metallylene step-by-step. Two experimentally viable germylene species (**CGeN–4** and **CGeP–4**) were selected to evaluate the trend in reactivity upon changing the Group 15 ligand.^6*b*^ These two germylene species are chosen for their resemblance to the model germylene, having a germanium central metallylene atom, one carbon and one nitrogen or phosphorus bearing ligand. In Fig. 6, we show the transition state structures of the H_2_ activation using the model germylene of the first part of this study (**CGeX–TS1**), two intermediate germylenes (**CGeX–TS3** and **CGeX–TS2**), and the complete germylene species (**CGeX–TS4**). Because the position of the transition state, along the reaction coordinate, remains relatively constant upon increasing the molecular complexity of the germylene's ligands, we perform an ASA on the transition states. The reaction barrier for both germylenes, **CGeN** and **CGeP**, slightly increases when going from the model germylene **CGeX–1** to the realistic germylene **CGeX–4**. This effect can predominantly be ascribed to a less stabilizing interaction energy, which, in turn, is caused by a more destabilizing Pauli repulsion between the increasingly bulkier ligands and H_2_ (Table S13†). The electrostatic and orbital interaction, on the other hand, become more stabilizing when the molecular complexity of the metallylene increases. In addition, for a few instances, an enhancement of destabilizing activation strain also contributes to the increase in reaction barrier, due to the increased rigidity of the ligands.

The analysis performed here also shows that the reactivity trends observed for our model metallylenes agree with, and therefore are a faithful representation of. the realistic, synthesizable metallylenes. All trends in reactivity, going from the model germylene **CGeX–1** to the realistic germylene **CGeX–4**, are identical, namely, the reaction barrier of **CGeN** is always higher in energy than the **CGeP** analog. In line with our detailed analysis displayed in Fig. 4, we find that the more destabilizing Pauli repulsion and, therefore, less favorable interaction energy is the main actor behind the higher reaction barrier of **CGeN** compared to **CGeP** (Table S13†).

### Expanding the substrate scope

**Fig. 7:**
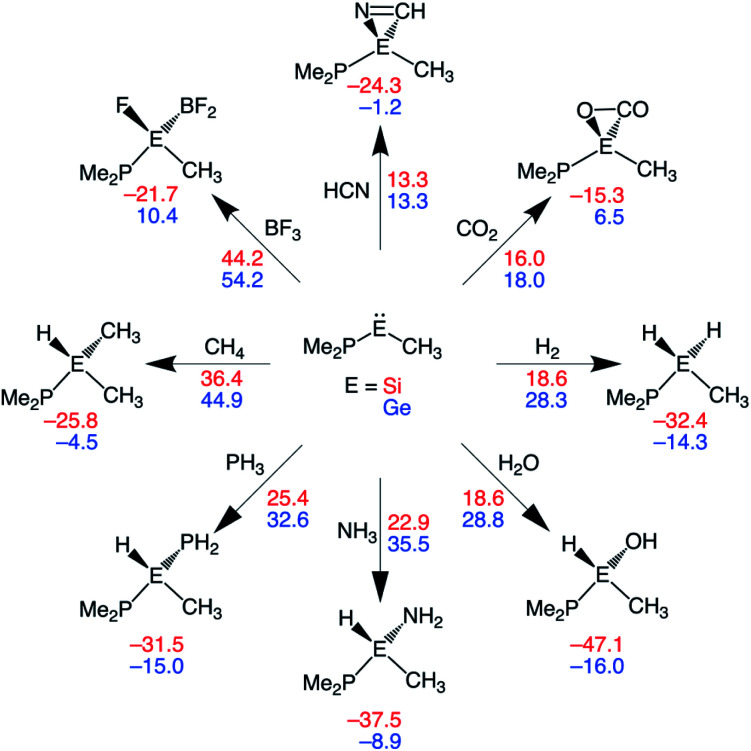
Gibbs free energy reaction barriers (Δ*G*^‡^; next to arrow) and reaction energies (Δ*G*_rxn_; below product) (in kcal mol^−1^) of the activation of various small molecules by **CEP** metallylenes, where E = Si, Ge (red, blue values). All data computed at ZORA-BP86/TZ2P.

In this section, we assess the ability of our rationally designed metallylenes to activate other small molecules, such as HCN, CO_2_, H_2_O, NH_3_, PH_3_, CH_4_, and BF_3_. **CSiP** and **CGeP** are selected, where the former (**CSiP**) exhibits a low reaction barrier for activation of H_2_ and a not too exergonic reaction energy, while the latter (**CGeP**) metallylene, as we prior showed, closely resembles an experimentally feasible metallylene.^6*b*^ In Fig. 7, we show the Gibbs free reaction barriers and reaction energies for the activation of the aforementioned small molecules. Note that, starting with HCN activation, the processes systematically increase in reaction barrier when following the processes in the wind-rose scheme in a clockwise direction. Interestingly, we find that both HCN and CO_2_ have a lower reaction barrier than our model substrate H_2_. This likely originates from the fact that for the former two substrates only a π-bond is broken, but for the latter, a strong σ-bond is dissociated, which goes with a high activation strain. The activation of H_2_O, NH_3_, and PH_3_ go with reaction barriers that are in the same range as the one for H_2_, making them, as shown in the literature,^5*a*,6*a*–*c*,7*b*^ possible candidates to be activated by metallylenes. In contrast, the reaction barriers for the activation of CH_4_ and BF_3_ are relatively high and, therefore, require further optimization to become suitable targets for activation by metallylenes. Furthermore, in analogy with the activation of H_2_, the activation using **CSiP** goes with a lower reaction barrier than and more stable product compared to **CGeP**. One *exception* is the activation of HCN, for which the reaction barriers of **CSiP** and **CGeP** are identical. These results highlight that the herein used tailor-made metallylenes can be extrapolated to the activation of a wide range of different small molecules.

### Hydrogenation of unsaturated bonds

In the last section, we study the viability of the H_2_ activated metallylene species to react in subsequent transformations. As discussed above, several studies solely focus on the activation of H_2_ and other small molecules by metallylenes and do not consider any follow up reactions. Recently, Bertrand *et al.*^4*d*^ showed that activated carbene species can hydrogenate terminal alkynes to alkenes. However, up to our knowledge, very little is actually known concerning the hydrogenation of unsaturated bonds by H_2_ activated metallylenes.

**Fig. 8:**
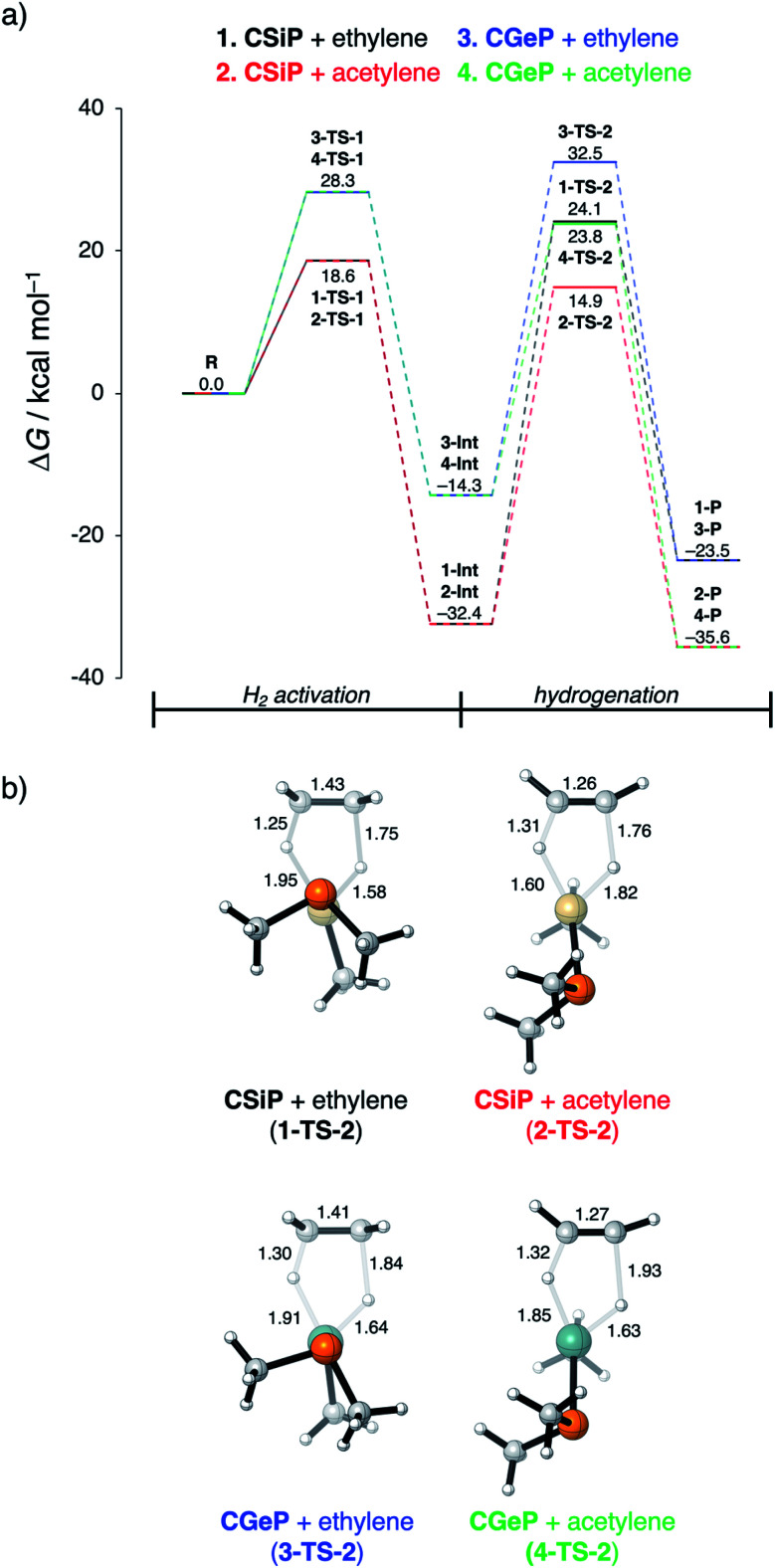
(a) Gibbs free energy profile (in kcal mol^−1^) for H_2_ activation and subsequent hydrogenation of ethylene or acetylene by **CEP** metallylenes (E = Si, Ge) computed at ZORA-BP86/TZ2P. (b) Transition state structures with key bond lengths (in Å) for the hydrogenation of ethylene or acetylene.

In Fig. 8, we show the reaction profiles of the activation of H_2_ by **CSiP** or **CGeP** and the subsequent hydrogenation of ethylene or acetylene to ethane and ethylene, respectively. As previously discussed in detail, the activation of H_2_ by **CSiP** goes with a significantly lower reaction barrier (**TS-1**) than the activation by **CGeP** (ΔΔ*G*^‡^ = 9.7 kcal mol^−1^). Furthermore, the former reaction is also more exergonic compared to the latter, resulting in a more stable intermediate (ΔΔ*G***Int** = 18.1 kcal mol^−1^), which is, as we will show later, of great importance for the follow-up hydrogenation reaction. As shown in Fig. 8b, all hydrogenation reactions occur in a concerted asynchronous fashion, where one newly formed C–H bond forms ahead of the other. The reaction barrier of the hydrogenation step (**TS-2**), relative to the intermediate **Int**, is for all metallylenes higher in energy than the H_2_ activation step 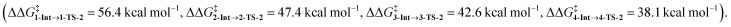 In addition, it can be seen that, in contrast with the H_2_ activation barriers, the hydrogenation reaction barriers are higher for **CSiP** than for **CGeP**.

These results indicate that metallylenes, which can efficiently activate H_2_ with a low reaction barrier, are not by definition good metallylenes for hydrogenation. A highly exergonic H_2_ activation reaction, in this mechanism, goes hand in hand with strong E–H bonds in **CEXH2**. Breaking these strong E–H bonds, in a subsequent hydrogenation step, will give rise to a high activation strain and hence a high hydrogenation reaction barrier. Thus, one must consider both the H_2_ activation barrier along with the associated reaction energies and ensure that the latter are only moderately exergonic so that the follow-up hydrogenation barrier is not prohibitively high.

## Conclusion

Our quantum chemical exploration, based on the activation strain model and Kohn–Sham molecular orbital theory, highlight the factors that determine the trends in reactivity of the H_2_ activation by various metallylenes H_3_C–E–X (**CEX**: E = C, Si, Ge, Sn, and X = NMe_2_, PMe_2_, AsMe_2_). Upon changing the central metallylene atom down in Group 14, from carbon to tin, while keeping the ligand consistent, systematically increases the H_2_ activation barrier. In contrast, varying the ligand X, from NMe_2_ to PMe_2_ to AsMe_2_, while keeping the central atom E constant results in a significant lowering of the reaction barrier.

We found that the increasing reaction barrier, on going from C to Sn, is caused by a reduced metallylene–H_2_ interaction that was traced back to less stabilizing orbital interactions. Along this series, the back-donation interaction between filled lone-pair orbital of the metallylene and the σ*-orbital of H_2_, *i.e.*, HOMO**CEX**–LUMO_H_2__, becomes progressively weaker, due to both an increased orbital energy gap, as the **CEX** HOMO goes up in energy (destabilized), as well as a reduced orbital overlap. Furthermore, the destabilization of the reaction barrier, as a response to changing the ligand down in Group 15, can be ascribed to two factors, namely, (i) reduced Pauli repulsive occupied–occupied orbital overlap between the reactants, because the ligand becomes more pyramidal and, therefore, polarizes the HOMO amplitude away from the incoming H_2_; and (ii) less activation strain, as the degree of pyramidalization of the ligand, upon reacting, becomes increasingly smaller.

At last, we extended our work to demonstrate, for the first time, that H_2_ activated metallylenes might be utilized in subsequent reactions. We exhibited that the rationally designed metallylenes are able, after the activation of H_2_, to hydrogenate ethylene and acetylene in one concerted asynchronous reaction step to ethane and ethylene, respectively. The reaction barrier corresponding to this reaction step is, however, higher in energy than the H_2_ activation step and additional tuning of the metallylene is necessary. Thus, to realize the full potential of metallylenes, one must carefully tune the exergonicity of the bond activation step, to reduce the strength of the E–H bonds, and also the reaction barrier of the subsequent reaction step, such as the hydrogenation reaction of unsaturated bonds.

## Conflicts of interest

The authors declare no conflict of interest.

## Supplementary Material

SC-012-D0SC05987K-s001
