# Supplementary material for: How metallylenes activate small molecules
Source: Chem Sci. 2021 Feb 18;12(12):4526–35. doi: 10.1039/d0sc05987k (PMC8179460; doi:10.1039/d0sc05987k)
Supplement: SC-012-D0SC05987K-s001 [file SC-012-D0SC05987K-s001.pdf]

Electronic Supplementary Material (ESI) for Chemical Science.
This journal is © The Royal Society of Chemistry 2021

Contents

Computational Detail

Table S1. Electronic reaction barriers ($\Delta E^{\ddagger}$), Gibbs free energy barriers ($\Delta G^{\ddagger}$), electronic reaction energies (ΔE_{rxn}), and Gibbs free reaction energies (ΔG_{rxn}) (in kcal mol^{-1}) of the activation of H_2 by H_3C–E–X metallylenes, computed at COSMO(toluene)-ZORA-BP86/TZ2P.

Table S2. Electronic reaction barriers ($\Delta E^{\ddagger}$), Gibbs free energy barriers ($\Delta G^{\ddagger}$), electronic reaction energies (ΔE_{rxn}), and Gibbs free reaction energies (ΔG_{rxn}) (in kcal mol^{-1}) of the activation of H_2 by H_3C–E–X metallylenes, computed at COSMO(water)-ZORA-BP86/TZ2P.

Table S3. Electronic reaction barriers ($\Delta E^{\ddagger}$), Gibbs free energy barriers ($\Delta G^{\ddagger}$), electronic reaction energies (ΔE_{rxn}), and Gibbs free reaction energies (ΔG_{rxn}) (in kcal mol^{-1}) of the activation of H_2 by H_3C–E–X metallylenes, computed at ZORA-BP86-D3(BJ)/TZ2P.

Table S4. Electronic reaction barriers ($\Delta E^{\ddagger}$) and reaction energies (ΔE_{rxn}) (in kcal mol^{-1}) of the activation of H_2 by H_3C–E–X metallylene catalysts, computed at ZORA-M06-2X/TZ2P// ZORA-BP86-D3(BJ)/TZ2P.

Table S5. Electronic reaction barriers ($\Delta E^{\ddagger}$) and reaction energies (ΔE_{rxn}) (in kcal mol^{-1}) of the activation of H_2 by H_3C–E–X metallylenes, computed at ZORA-M06-2X/TZ2P//ZORA-BP86/TZ2P.

Table S6. Electronic reaction barriers ($\Delta E^{\ddagger}$) and reaction energies (ΔE_{rxn}) (in kcal mol^{-1}) of the activation of H_2 by H_3C–E–X metallylenes, computed at DLPNO-CCSD(T)/def2-QZVPP//ZORA-BP86/TZ2P.

Table S7. Statistical analysis of the used XC functionals (in kcal mol^{-1}): mean absolute deviation $\Delta E^{\ddagger}_{MAD}$ and standard deviation mean deviation $\Delta E^{\ddagger}_{SD}$ relative to DLPNO-CCSD(T)/def2-QZVPP//ZORA-BP86/TZ2P computed electronic reaction barriers of the activation of H_2 by H_3C–E–X metallylenes.

Table S8. Highest occupied molecular orbital energy (ε_{HOMO}), lowest unoccupied molecular orbital energy (ε_{LUMO}), and HOMO–LUMO energy gap ($\varepsilon_{H–L}$) (in eV) of the H_3C–E–X metallylenes, computed at ZORA-BP86/TZ2P.

Table S9. Highest occupied molecular orbital energy (ε_{HOMO}), lowest unoccupied molecular orbital energy (ε_{LUMO}), and HOMO–LUMO energy gap ($\varepsilon_{H–L}$) (in eV) of the H_3C–E–X metallylenes, computed at ZORA-M06-2X/TZ2P//ZORA-BP86/TZ2P

Table S10. Highest occupied molecular orbital energy (ε_{HOMO}), lowest unoccupied molecular orbital energy (ε_{LUMO}), and HOMO–LUMO energy gap ($\varepsilon_{H–L}$) (in eV) of the H_3C–E–X metallylenes, computed at SAOP/TZ2P//ZORA-BP86/TZ2P.

Table S11. Statistical analysis of the used XC functionals (in kcal mol^{-1}): mean absolute deviation $\Delta E^{\ddagger}_{MAD}$, standard deviation mean deviation $\Delta E^{\ddagger}_{SD}$, maximum negative $\Delta E^{\ddagger}_{max(-)}$ and positive negative $\Delta E^{\ddagger}_{max(+)}$ error relative to SAOP/TZ2P//ZORA-BP86/TZ2P computed HOMO–LUMO orbital energy gaps of the H_3C–E–X metallylenes.

Fig. S1. Transition state structures for the activation of H_2 by H_3C–E–X (**CEX**) metallylenes, computed at ZORA-BP86/TZ2P.

Fig. S2. a) Activation strain analysis and b) energy decomposition analysis of the H_2 bond activation by metallylenes **CEP** with variating Group 14 central atom (E = C, Si, Ge, Sn), energies are projected onto the H•••H bond stretch of H_2, computed at ZORA-BP86/TZ2P.

Fig. S3. a) Activation strain analysis and b) energy decomposition analysis of the H_2 bond activation by metallylenes **CEAs** with variating Group 14 central atom (E = C, Si, Ge, Sn), energies are projected onto the H•••H bond stretch of H_2, computed at ZORA-BP86/TZ2P.

Fig. S4 (a) Activation strain analysis and (b) energy decomposition analysis of the H_2 bond activation by metallylenes **CEN** with variating Group 14 central atom (E = C, Si, Ge, Sn), where the transition states are indicated with a dot and the energies are projected onto the H•••H bond stretch of H_2, computed at ZORA-M06-2X/TZ2P//ZORA-BP86/TZ2P.

Table S12. Activation strain and energy decomposition analyses (in kcal mol^{-1}) of the H_2 bond activation by metallylenes **CEN** with variating Group 14 central atom (E = C, Si, Ge, Sn).

Fig. S5. a) Activation strain analysis and b) energy decomposition analysis of the H_2 bond activation by metallylenes **CCX** with variating Group 14 central atom (X = NMe_2, PMe_2, $AsMe_2$), where the energies are projected onto the H•••H bond stretch of H_2, computed at ZORA-BP86/TZ2P.

Fig. S6. a) Activation strain analysis and b) energy decomposition analysis of the H_2 bond activation by metallylenes **CSiX** with variating Group 14 central atom (X = NMe_2, PMe_2, $AsMe_2$) where the energies are projected onto the H•••H bond stretch of H_2, computed at ZORA-BP86/TZ2P.

Fig. S7. a) Activation strain analysis and b) energy decomposition analysis of the H_2 bond activation by metallylene **CSnX** with variating Group 14 central atom (X = NMe_2, PMe_2, $AsMe_2$) where the energies are projected onto the H•••H bond stretch of H_2, computed at ZORA-BP86/TZ2P.

Fig. S8 (a) Activation strain analysis and (b) energy decomposition analysis of the H_2 bond activation by germylenes **CGeX** with variating Group 15 ligands (X = NMe_2, PMe_2, $AsMe_2$), where the transition states are indicated with a dot and the energies are projected onto the H•••H bond stretch of H_2, computed at ZORA-M06-2X/TZ2P//ZORA-BP86/TZ2P.

Fig. S9. Representations of the HOMO–1 orbital of the various **CGeX** metallylenes (X = N, P, As) in their equilibrium geometry (isovalue = 0.03 Bohr$^{-3/2}$) and the orbital overlap of the hyperconjugation interaction between the 4p atomic orbital of germanium and the np atomic orbital of the ligand X, computed using ZORA-BP86/TZ2P.

Table S13. Activation strain and energy decomposition analyses (in kcal mol^{-1}) of for the H_2 activation by the synthesizable metallylene species **CGeX–4** (where X = N, P) and the three stages of simplified analogs.

Table S14. Cartesian coordinates (in Å), energies (in kcal mol^{-1}), and number of imaginary frequencies of all stationary points, computed at ZORA-BP86/TZ2P.

Table S15. Cartesian coordinates (in Å), energies (in kcal mol^{-1}), and number of imaginary frequencies of all stationary points, computed at ZORA-BP86-D3(BJ)/TZ2P.

Table S16. Cartesian coordinates (in Å), energies (in kcal mol^{-1}), and number of imaginary frequencies of all stationary points, computed at COSMO(toluene)ZORA-BP86/TZ2P.

Table S17. Cartesian coordinates (in Å), energies (in kcal mol^{-1}), and number of imaginary frequencies of all stationary points, computed at COSMO(water)ZORA-BP86/TZ2P.

Computational Details

All calculations were performed using the Amsterdam Density Functional (ADF2018.104) software package.[1] The GGA exchange-correlation functional BP86[2] was used for the optimization of all stationary points and analyses along the reaction coordinate using the activation strain model (ASM)[3] of reactivity together with a matching energy decomposition analysis (EDA)[4] scheme. scheme. Scalar relativistic effects are accounted for using the zeroth-order regular approximation (ZORA).[5] The basis set used, denoted TZ2P,[6] is of triple-ζ quality for all atoms and has been improved by two sets of polarization functions. Additionally, stationary points were re-optimized using the dispersion-corrected ZORA-BP86-D3(BJ)[7]/TZ2 P to assess the effects of dispersion-corrections on the computed reactivity trends. Furthermore, single-point energies were computed at M06-2X[8]/TZ2P on fully optimized ZORA-BP86-D3(BJ)/TZ2P geometries to identify the effect of meta-hybrid exchange-correlation functionals on the computed reactivity trends. In addition, stationary points were re-optimized at COSMO[9](toluene)-ZORA-BP86/TZ2P and COSMO(water)-ZORA-BP86/TZ2P in order to assess the effect of solvation on the computed reactivity trends. The Domain Based Local Pair-Natural Coupled-Cluster (DLPNO-CCSD(T))[10] calculations, with NormalPNO, were performed using Orca 4.0.1[11] using the def2-QZVPP[12] basis set on ZORA-BP86/TZ2P

---

1 a) G. te Velde, F. M. Bickelhaupt, E. J. Baerends, C. Fonseca Guerra, S. J. A. van Gisbergen, J. G. Snijders and T. Ziegler, *J. Comput. Chem.* 2001, **22**, 931; b) C. Fonseca Guerra, J. G. Snijders, G. te Velde and E. J. Baerends, *Theor. Chem. Acc.* 1998, **99**, 391; c) ADF2018.104, SCM Theoretical Chemistry, Vrije Universiteit: Amsterdam (Netherlands). http:// www.scm.com.

2 a) A. D. Becke, *Phys. Rev. A*, 1988, **38**, 3098; b) J. P. Perdew, *Phys. Rev. B: Condens. Matter Mater. Phys.* 1986, **33**, 8822.

3 For a step-by-step protocol, see: a) P. Vermeeren, S. C. C. van der Lubbe, C. Fonseca Guerra, F. M. Bickelhaupt and T. A. Hamlin, *Nat. Protoc.* 2020, **15**, 649. For reviews, see: b) F. M. Bickelhaupt, *J. Comp. Chem.* 1999, **20**, 114; c) I. Fernández and F. M. Bickelhaupt, *Chem. Soc. Rev.* 2014, **43**, 4953; d) F. M. Bickelhaupt and K. N. Houk, *Angew. Chem. Int. Ed.* 2017, **56**, 10070; *Angew. Chem.* 2017, **129**, 10204.

4 a) F. M. Bickelhaupt and E. J. Baerends, in *Reviews in Computational Chemistry* (Eds.: K. B. Lipkowitz, D. B. Boyd), Wiley, Hoboken, 2000, pp. 1–86; b) R. van Meer, O. V. Gritsenko and E. J. Baerends, *J. Chem. Theory Comput.* 2014, **10**, 4432.

5 a) E. van Lenthe, E. J. Baerends and J. G. Snijders, *J. Chem. Phys.* 1993, **99**, 4597; b) E. van Lenthe, E. J. Baerends and J. G. Snijders, *J. Chem. Phys.* 1994, **101**, 9783.

6 E. van Lenthe and E. J. Baerends, *J. Comput. Chem.* 2003, **24**, 1142.

7 a) S. Grimme, J. Antony, S. Ehrlich and H. A. Krieg, *J. Chem. Phys.* 2010, **132**, 154104; b) S. Grimme, S. Ehrlich and L. Goerigk. *J. Comput. Chem*. 2011, **32**, 1456.

8 a) Y. Zhao and D. G. Truhlar, *J. Chem. Phys*. 2006, **125**, 194101; b) Y. Zhao and D. G. Truhlar, *Theor. Chem. Acc.* 2008, **120**, 215.

9 a) A. Klamt and G. Schüürmann, *J. Chem. Soc. Perkin Trans. 2* 1993, 799; b) A. Klamt, *J. Phys. Chem.* 1995, **99**, 2224; c) A. Klamt and V. Jonas, *J. Chem. Phys.* 1996, **105**, 9972; d) C. C. Pye and T. Ziegler, *Theor. Chem. Acc.* 1999, **101**, 396.

10 a) F. Neese, *WIREs Comput. Mol. Sci.* 2018, **8**, e1327; b) C. Riplinger, B. Sandhoefer, A. Hansen, F. Neese, *J. Chem. Phys*. 2013, **139**, 134101.

11 F. Neese, *WIREs Comput. Mol. Sci*. 2018, **8**, e1327.

12 a) F. Weigend, R. Ahlrichs, *Phys. Chem. Chem. Phys.* 2005, **7**, 3297; b) F. Weigend, *Phys. Chem. Chem. Phys.* 2006, **8**, 1057.

geometries. These data all show the same trends in reactivity for the investigated reactions and displayed that all ZORA-BP86/TZ2P and ZORA-M06-2X/TZ2P//ZORA-BP86/TZ2P perform equally good (Tables S1-S7). The accuracy of the HOMO–LUMO energy gaps are quantified by comparing the ZORA-BP86/TZ2P and ZORA-M06-2X/TZ2P//ZORA-BP86/TZ2P HOMO–LUMO energy gaps with the statistical average of orbital dependent potentials (SAOP),[13] which is a good approximation of the exact KS potential with eigenvalues close to the true KS spectrum,[14] computed at SAOP/TZ2P//ZORA-BP86/TZ2P. These results show that ZORA-BP86/TZ2P significantly outperforms ZORA-M06-2X/TZ2P//ZORA-BP86/TZ2P in determining accurate HOMO–LUMO energy gaps (Tables S8-S11). The accuracies of the fit scheme and the integration grid, Zlm fit and Becke grid, respectively,[15] were set to VERYGOOD. Geometries were optimized without any symmetry constraint. All calculated stationary points have been verified, through vibrational analysis,[16] to be energy minima (zero imaginary frequencies) or transition states (one imaginary frequency). The character of the normal mode associated with the imaginary frequency of the transition state has been analyzed to ensure it is associated with the reaction of interest. The potential energy surfaces of the studied dihydrogen activation reactions using metallylenes were obtained by performing intrinsic reaction coordinate (IRC) calculations.[17] The obtained potential energy surfaces are analyzed using the PyFrag program.[18] All chemical structures were illustrated using CYLview.[19]

13 a) O. V. Gritsenko, P. R. T. Schipper and E. J. Baerends, *Chem. Phys. Lett.*, 1999, **302**, 199; b) P. R. T. Schipper, O. V. Gritsenko, S. J. A. van Gisbergen and E. J. Baerends, *J. Chem. Phys.*, 2000, **112**, 1344.

14 a) D. P. Chong, O. V. Gritsenko and E. J. Baerends, *J. Chem. Phys.*, 2002, **116**, 1760; b) M. Grüning, O. Gritsenko, S. J. A. van Gisbergen and E. J. Baerends, *J. Chem. Phys.*, 2002, **116**, 9591.

15 a) M. Franchini, P. H. T. Philipsen, E. van Lenthe and L. Visscher, *J. Chem. Theory Comput.* 2014, **10**, 1994; b) M. Franchini, P. H. T. Philipsen and L. Visscher, *J. Comput. Chem.* 2013, **34**, 1819.

16 a) A. Bérces, R. M. Dickson, L. Fan, H. Jacobsen, D. P. Swerhone and T. Ziegler, *Comput. Phys. Commun.* 1997, **100**, 247; b) H. Jacobsen, A. Bérces, D. P. Swerhone and T. Ziegler, *Comput. Phys. Commun.* 1997, **100**, 263; c) S. K. Wolff, *Int. J. Quantum Chem.* 2005, **104,** 645.

17 a) K. Fukui, *Acc. Chem. Res.* 1981, **14**, 363; b) L. Deng, T. Ziegler and L. A. Fan, *J. Chem. Phys.* 1993, **99**, 3823; c) L. Deng and T. Ziegler, *Int. J. Quantum Chem.* 1994, **52**, 731.

18 X. Sun, T. M. Soini, J. Poater, T. A. Hamlin and F. M. Bickelhaupt, *J. Comp. Chem.* 2019, **40**, 2227.

19 Legault, C. Y., CYLview, 1.0b; Université de Sherbrooke, Canada, Sherbrooke, QC, 2009, http://www.cylview.org.

Table S1. Electronic reaction barriers ($\Delta E^{\ddagger}$), Gibbs free energy barriers ($\Delta G^{\ddagger}$), electronic reaction energies (ΔE_{rxn}), and Gibbs free reaction energies (ΔG_{rxn}) (in kcal mol^{-1}) of the activation of H_2 by H_3C–E–X metallylenes, computed at COSMO(toluene)-ZORA-BP86/TZ2P.

E	X	$\Delta E^{\ddagger}$	$\Delta G^{\ddagger}$	ΔE_{rxn}	ΔG_{rxn}
C	NMe_2	12.9	20.8	–55.6	–38.9
Si	NMe_2	24.7	33.1	–35.1	–24.4
Ge	NMe_2	37.8	45.4	–13.0	–2.7
Ge	PMe_2	18.6	28.3	–26.0	–14.4
Ge	$AsMe_2$	13.7	23.1	–30.0	–18.6
Sn	NMe_2	48.2	55.5	1.2	9.9

Table S2. Electronic reaction barriers ($\Delta E^{\ddagger}$), Gibbs free energy barriers ($\Delta G^{\ddagger}$), electronic reaction energies (ΔE_{rxn}), and Gibbs free reaction energies (ΔG_{rxn}) (in kcal mol^{-1}) of the activation of H_2 by H_3C–E–X metallylenes, computed at COSMO(water)-ZORA-BP86/TZ2P.

E	X	$\Delta E^{\ddagger}$	$\Delta G^{\ddagger}$	ΔE_{rxn}	ΔG_{rxn}
C	NMe_2	11.2	18.8	–52.5	–35.1
Si	NMe_2	25.3	33.5	–34.5	–23.8
Ge	NMe_2	37.5	44.9	–13.3	–3.0
Ge	PMe_2	18.6	28.3	–25.9	–14.5
Ge	$AsMe_2$	14.4	23.7	–29.2	–16.7
Sn	NMe_2	47.5	54.7	0.6	9.3

Table S3. Electronic reaction barriers ($\Delta E^{\ddagger}$), Gibbs free energy barriers ($\Delta G^{\ddagger}$), electronic reaction energies (ΔE_{rxn}), and Gibbs free reaction energies (ΔG_{rxn}) (in kcal mol^{-1}) of the activation of H_2 by H_3C–E–X metallylenes, computed at ZORA-BP86-D3(BJ)/TZ2P.

E	X	$\Delta E^{\ddagger}$	$\Delta G^{\ddagger}$	ΔE_{rxn}	ΔG_{rxn}
C	NMe_2	10.0	19.4	–60.1	–43.3
Si	NMe_2	23.3	31.4	–36.4	–25.8
Ge	NMe_2	37.2	44.6	–13.7	–3.4
Ge	PMe_2	17.0	25.8	–27.9	–17.0
Ge	$AsMe_2$	12.2	21.2	–31.7	–20.5
Sn	NMe_2	48.0	55.1	1.0	9.5

Table S4. Electronic reaction barriers ($\Delta E^{\ddagger}$) and reaction energies (ΔE_{rxn}) (in kcal mol^{-1}) of the activation of H_2 by H_3C–E–X metallylenes, computed at ZORA-M06-2X/TZ2P//ZORA-BP86-D3(BJ)/TZ2P.

E	X	$\Delta E^{\ddagger}$	ΔE_{rxn}
C	NMe_2	19.5	–63.9
Si	NMe_2	29.4	–38.8
Ge	NMe_2	41.2	–19.0
Ge	PMe_2	20.1	–33.5
Ge	$AsMe_2$	18.9	–33.5
Sn	NMe_2	55.1	–3.9

Table S5. Electronic reaction barriers ($\Delta E^{\ddagger}$) and reaction energies (ΔE_{rxn}) (in kcal mol^{-1}) of the activation of H_2 by H_3C–E–X metallylenes, computed at ZORA-M06-2X/TZ2P//ZORA-BP86/TZ2P.

E	X	$\Delta E^{\ddagger}$	ΔE_{rxn}
C	NMe_2	19.8	–64.1
Si	NMe_2	29.2	–38.8
Ge	NMe_2	41.1	–19.3
Ge	PMe_2	19.7	–33.6
Ge	$AsMe_2$	18.4	–34.0
Sn	NMe_2	54.8	–4.0

Table S6. Electronic reaction barriers ($\Delta E^{\ddagger}$) and reaction energies (ΔE_{rxn}) (in kcal mol^{-1}) of the activation of H_2 by H_3C–E–X metallylenes, computed at DLPNO-CCSD(T)/def2-QZVPP//ZORA-BP86/TZ2P.

E	X	$\Delta E^{\ddagger}$	ΔE_{rxn}
C	NMe_2	19.0	–63.7
Si	NMe_2	27.8	–40.7
Ge	NMe_2	36.9	–23.4
Ge	PMe_2	17.1	–37.5
Ge	$AsMe_2$	14.3	–40.1
Sn	NMe_2	50.7	–5.1

Table S7. Statistical analysis of the used XC functionals (in kcal mol^{-1}): mean absolute deviation $\Delta E^{\ddagger}_{MAD}$ and standard deviation mean deviation $\Delta E^{\ddagger}_{SD}$ relative to DLPNO-CCSD(T)/def2-QZVPP//ZORA-BP86/TZ2P computed electronic reaction barriers of the activation of H_2 by H_3C–E–X metallylene.

	ZORA-BP86/TZ2P	ZORA-M06-2X/TZ2P//ZORA-BP86/TZ2P	ZORA-BP86-D3(BJ)/TZ2P	ZORA-M06-2X/TZ2P//ZORA-BP86-D3(BJ)/TZ2P
$\Delta E^{\ddagger}_{MAD}$	2.8	2.9	3.1	3.1
$\Delta E^{\ddagger}_{SD}$	3.2	1.5	3.4	1.7

Table S8. Highest occupied molecular orbital energy (ε_{HOMO}), lowest unoccupied molecular orbital energy (ε_{LUMO}), and HOMO–LUMO energy gap ($\varepsilon_{H–L}$) (in eV) of the $H_3C–E–X$ metallylenes, computed at ZORA-BP86/TZ2P.

E	X	ε_{HOMO}	ε_{LUMO}	$\varepsilon_{H–L}$
C	NMe_2	–4.1	–1.0	3.1
C	PMe_2	–4.3	–1.5	2.8
C	$AsMe_2$	–4.5	–2.8	1.7
Si	NMe_2	–5.0	–2.1	2.9
Si	PMe_2	–5.2	–2.0	3.2
Si	$AsMe_2$	–4.8	–3.1	1.7
Ge	NMe_2	–5.3	–2.3	3.0
Ge	PMe_2	–5.0	–2.1	2.9
Ge	$AsMe_2$	–4.8	–3.3	1.5
Sn	NMe_2	–5.3	–2.6	2.7
Sn	PMe_2	–4.8	–2.7	2.1
Sn	$AsMe_2$	–4.7	–3.4	1.3

Table S9. Highest occupied molecular orbital energy (ε_{HOMO}), lowest unoccupied molecular orbital energy (ε_{LUMO}), and HOMO–LUMO energy gap ($\varepsilon_{H–L}$) (in eV) of the $H_3C–E–X$ metallylenes, computed at ZORA-M06-2X/TZ2P//ZORA-BP86/TZ2P

E	X	ε_{HOMO}	ε_{LUMO}	$\varepsilon_{HOMO–LUMO}$
C	NMe_2	–6.5	0.8	7.3
C	PMe_2	–6.5	0.3	6.8
C	$AsMe_2$	–6.8	–1.0	5.8
Si	NMe_2	–7.0	–0.5	6.5
Si	PMe_2	–6.9	–0.5	6.4
Si	$AsMe_2$	–6.6	–1.5	5.1
Ge	NMe_2	–7.3	–0.7	6.6
Ge	PMe_2	–6.7	–0.6	6.1
Ge	$AsMe_2$	–6.6	–1.6	5.0
Sn	NMe_2	–7.2	–1.1	6.1
Sn	PMe_2	–6.4	–1.1	5.3
Sn	$AsMe_2$	–6.4	–1.9	4.5

Table S10. Highest occupied molecular orbital energy (ε_{HOMO}), lowest unoccupied molecular orbital energy (ε_{LUMO}), and HOMO–LUMO energy gap ($\varepsilon_{H\text{–}L}$) (in eV) of the H_3C–E–X metallylenes, computed at SAOP/TZ2P//ZORA-BP86/TZ2P.

E	X	ε_{HOMO}	ε_{LUMO}	$\varepsilon_{HOMO\text{–}LUMO}$
C	NMe_2	–8.0	–4.8	3.2
C	PMe_2	–8.1	–5.1	3.0
C	$AsMe_2$	–8.3	–6.5	1.8
Si	NMe_2	–8.5	–5.6	2.9
Si	PMe_2	–8.7	–5.5	3.2
Si	$AsMe_2$	–8.3	–6.5	1.8
Ge	NMe_2	–9.0	–6.0	3.0
Ge	PMe_2	–8.5	–5.7	2.8
Ge	$AsMe_2$	–8.4	–6.8	1.6
Sn	NMe_2	–8.8	–6.2	2.6
Sn	PMe_2	–8.2	–6.1	2.1
Sn	$AsMe_2$	–8.2	–6.8	1.4

Table S11. Statistical analysis of the used XC functionals (in eV): mean absolute deviation $\Delta E^{\ddagger}_{MAD}$, standard deviation mean deviation $\Delta E^{\ddagger}_{SD}$, maximum negative $\Delta E^{\ddagger}_{max(-)}$ and positive negative $\Delta E^{\ddagger}_{max(+)}$ error relative to SAOP/TZ2P//ZORA-BP86/TZ2P computed HOMO–LUMO orbital energy gaps of the H_3C–E–X metallylenes.

	ZORA-BP86/TZ2P	ZORA-M06-2X/TZ2P// ZORA-BP86/TZ2P
$\Delta\varepsilon_{H\text{–}L,MAD}$	0.1	3.5
$\Delta\varepsilon_{H\text{–}L,SD}$	0.1	0.3
$\Delta\varepsilon_{H\text{–}L,max(-)}$	0.2	[a]
$\Delta\varepsilon_{H\text{–}L,max(+)}$	0.1	4.1

[a] No computed reaction barrier lower than the SAOP/TZ2P//ZORA-BP86/TZ2P value.

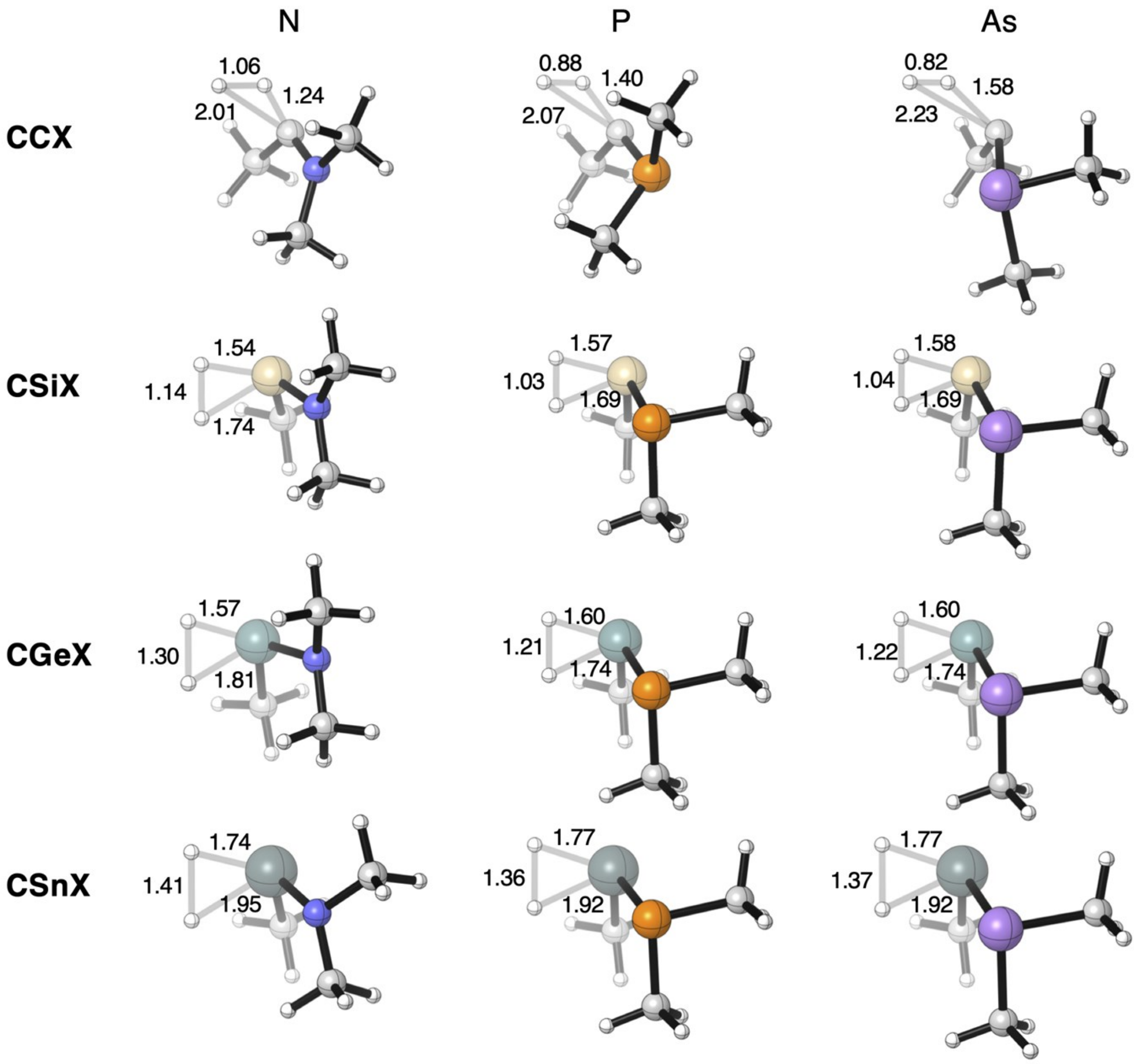

Fig. S1. Transition state structures for the activation of H_2 by H_3C–E–X (**CEX**) metallylenes, computed at ZORA-BP86/TZ2P.

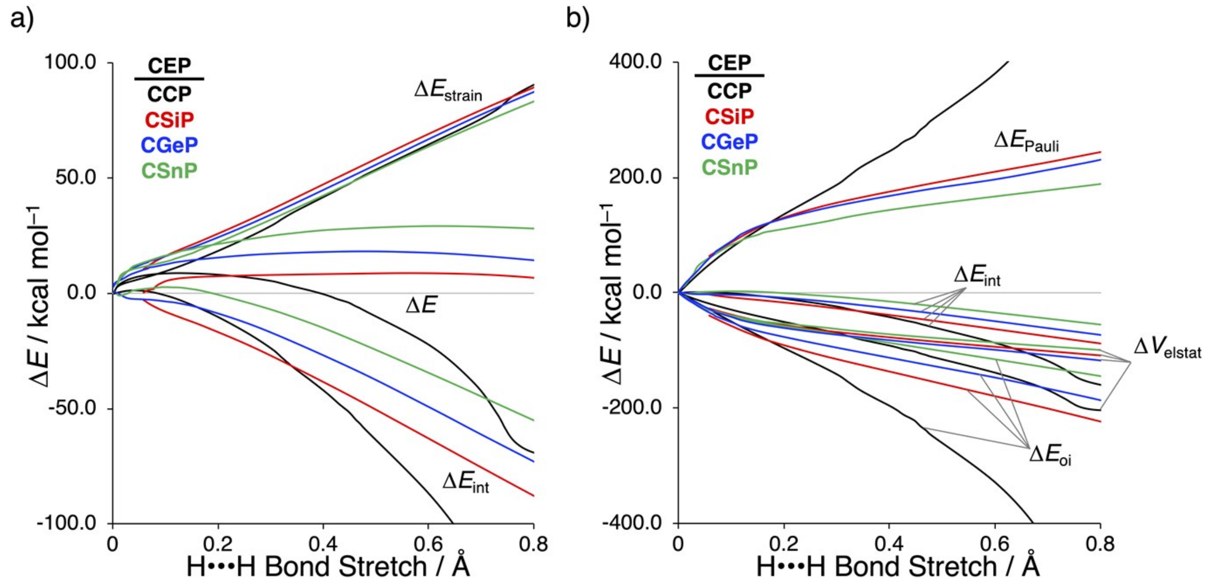

Fig. S2. (a) Activation strain analysis and (b) energy decomposition analysis of the H_2 bond activation by metallylenes **CEP** with variating Group 14 central atom (E = C, Si, Ge, Sn), energies are projected onto the H•••H bond stretch of H_2, computed at ZORA-BP86/TZ2P.

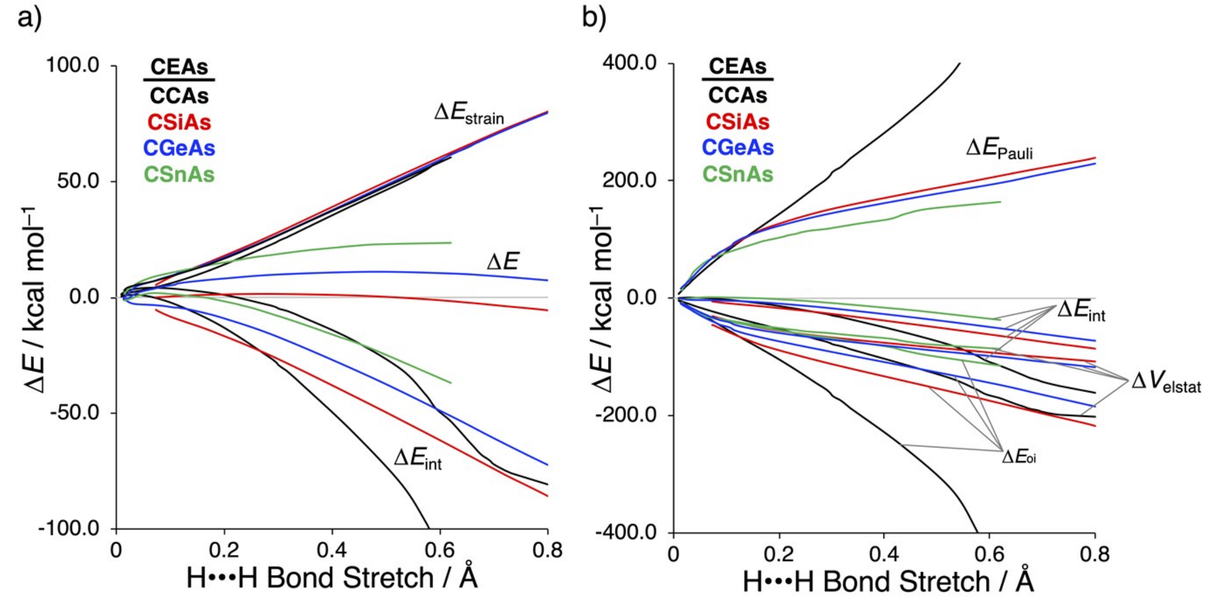

Fig. S3. (a) Activation strain analysis and (b) energy decomposition analysis of the H_2 bond activation by metallylenes **CEAs** with variating Group 14 central atom (E = C, Si, Ge, Sn), energies are projected onto the H•••H bond stretch of H_2, computed at ZORA-BP86/TZ2P.

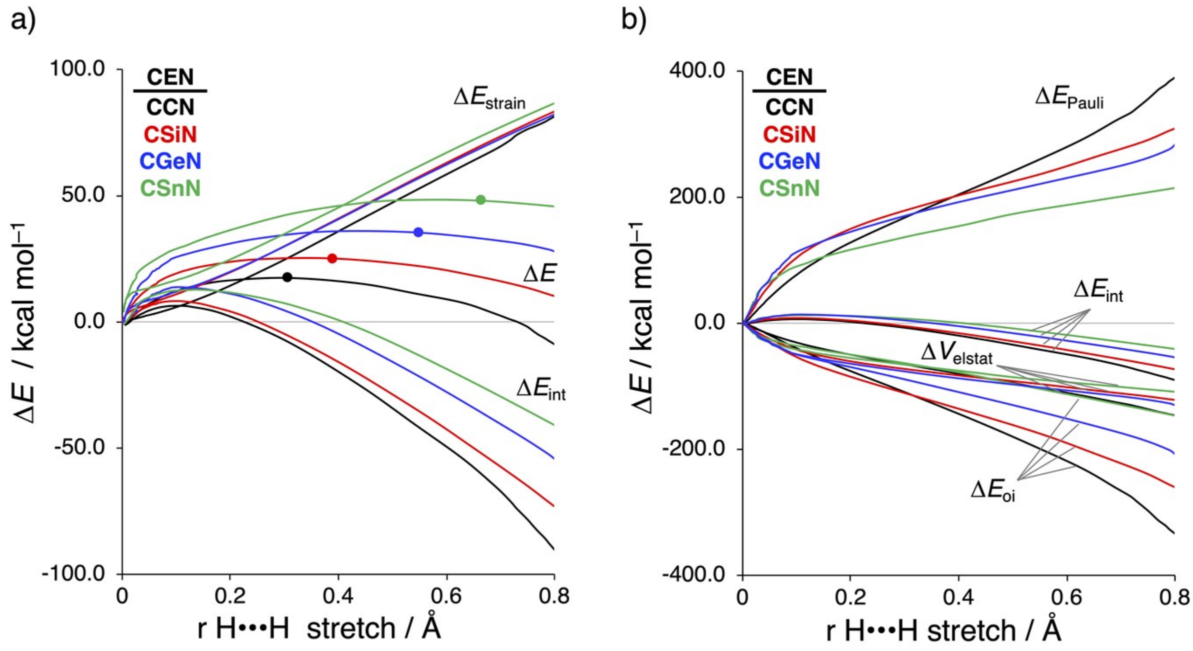

Fig. S4 (a) Activation strain analysis and (b) energy decomposition analysis of the H_2 bond activation by metallylenes **CEN** with variating Group 14 central atom (E = C, Si, Ge, Sn), where the transition states are indicated with a dot and the energies are projected onto the H•••H bond stretch of H_2, computed at ZORA-M06-2X/TZ2P//ZORA-BP86/TZ2P.

Table S12. Activation strain and energy decomposition analyses (in kcal mol^{-1}) of the H_2 bond activation by metallylenes **CEN** with variating Group 14 central atom (E = C, Si, Ge, Sn).[a]

CEN	ΔE^*	$\Delta E_{\mathrm{strain}}$	ΔE_{int}	$\Delta V_{\mathrm{elstat}}$	$\Delta E_{\mathrm{Pauli}}$	ΔE_{oi}
**CCN**	10.3	43.8	–33.4	–88.4	232.4	–177.4
**CSiN**	23.5	48.7	–25.2	–87.5	222.9	–160.6
**CGeN**	36.4	48.2	–11.8	–90.1	205.2	–127.0
**CSnN**	45.9	53.0	–7.0	–79.7	171.3	–98.6

[a] Analyses at consistent TS-like geometries, with a H•••H bond stretch of 0.47Å computed at ZORA-BP86/TZ2P level.

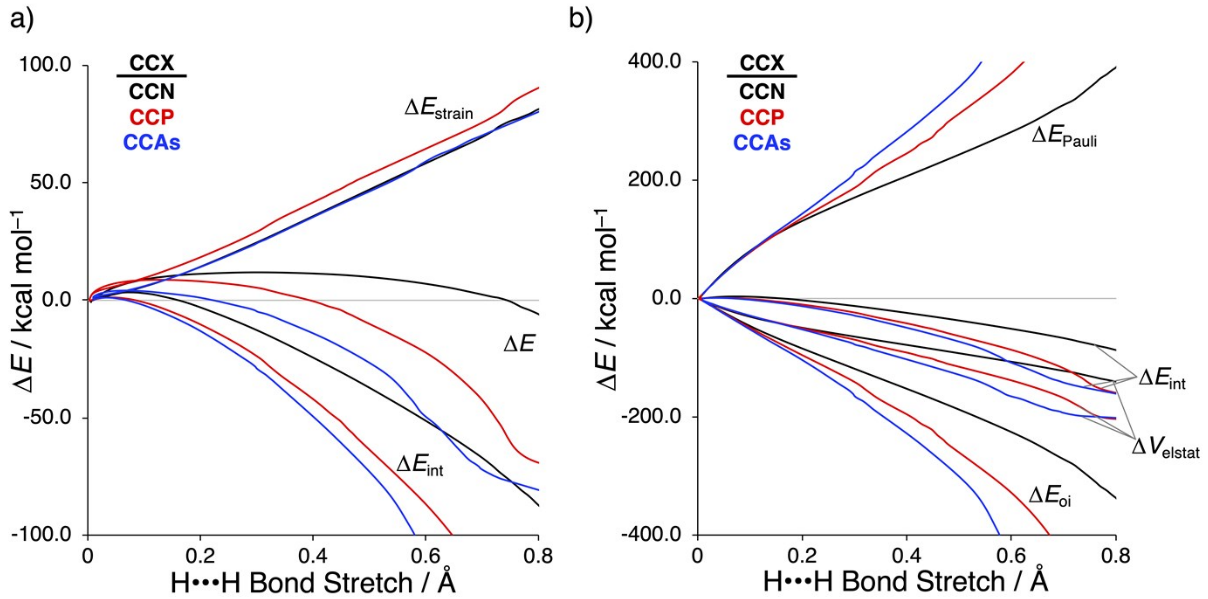

Fig. S5. (a) Activation strain analysis and (b) energy decomposition analysis of the H_2 bond activation by metallylenes **CCX** with variating Group 14 central atom (X = NMe_2, PMe_2, $AsMe_2$), where the energies are projected onto the H•••H bond stretch of H_2, computed at ZORA-BP86/TZ2P.

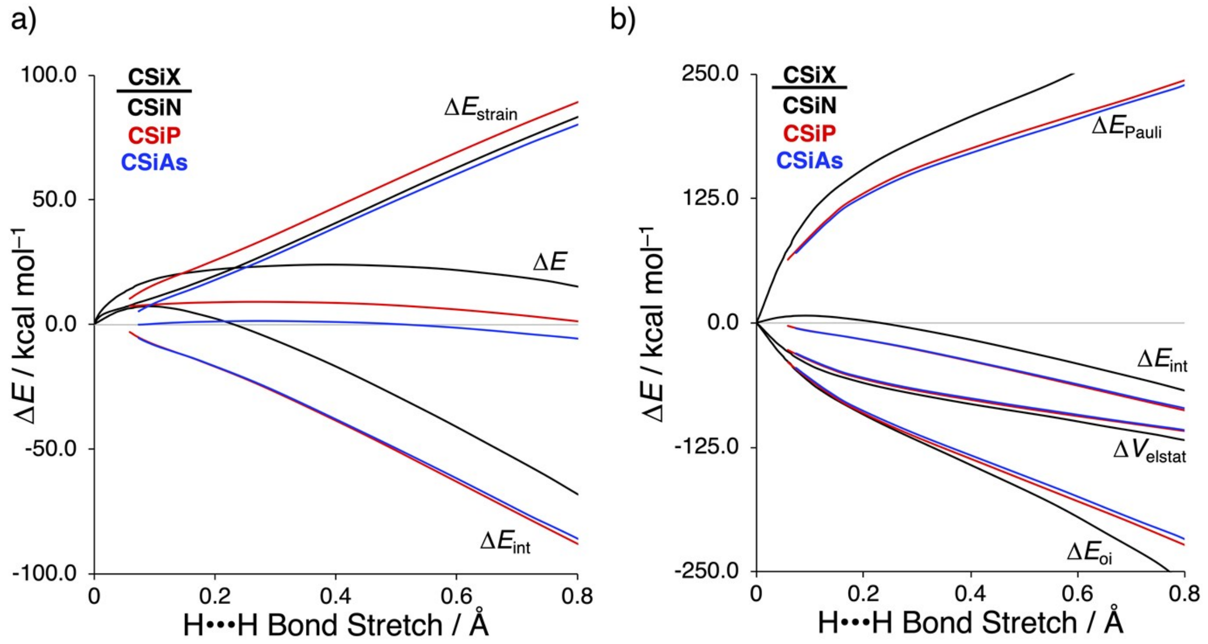

Fig. S6. (a) Activation strain analysis and (b) energy decomposition analysis of the H_2 bond activation by metallylenes **CSiX** with variating Group 14 central atom (X = NMe_2, PMe_2, $AsMe_2$) where the energies are projected onto the H•••H bond stretch of H_2, computed at ZORA-BP86/TZ2P.

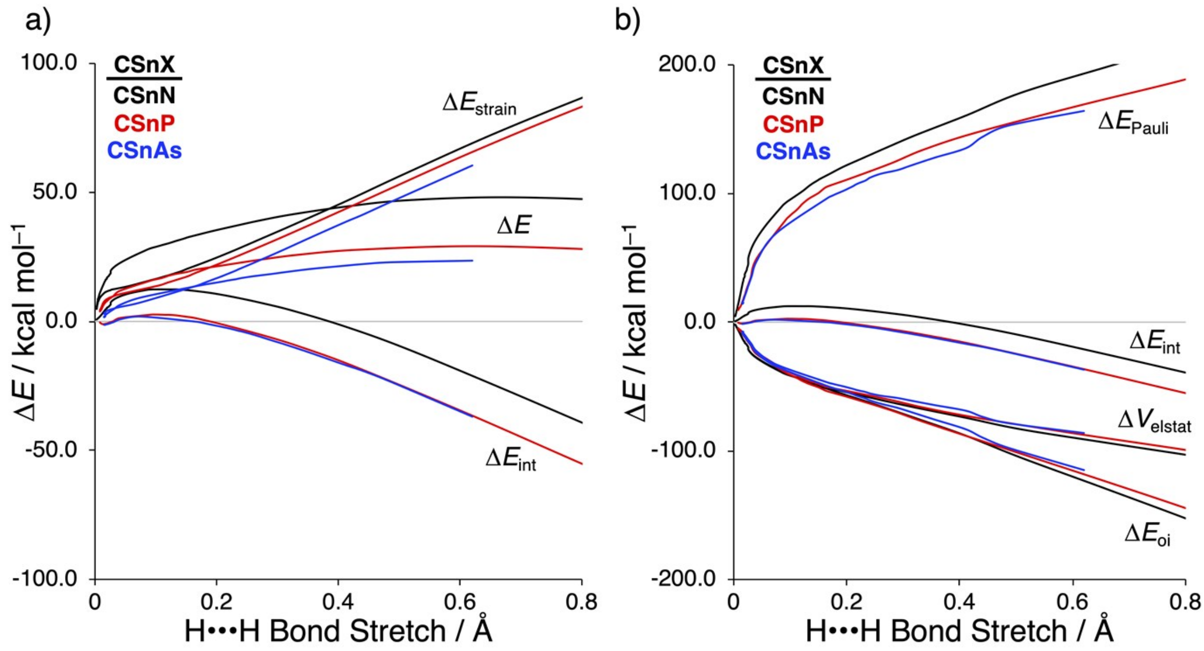

Fig. S7. (a) Activation strain analysis and (b) energy decomposition analysis of the H_2 bond activation by metallylene **CSnX** with variating Group 14 central atom (X = NMe_2, PMe_2, $AsMe_2$) where the energies are projected onto the H•••H bond stretch of H_2, computed at ZORA-BP86/TZ2P.

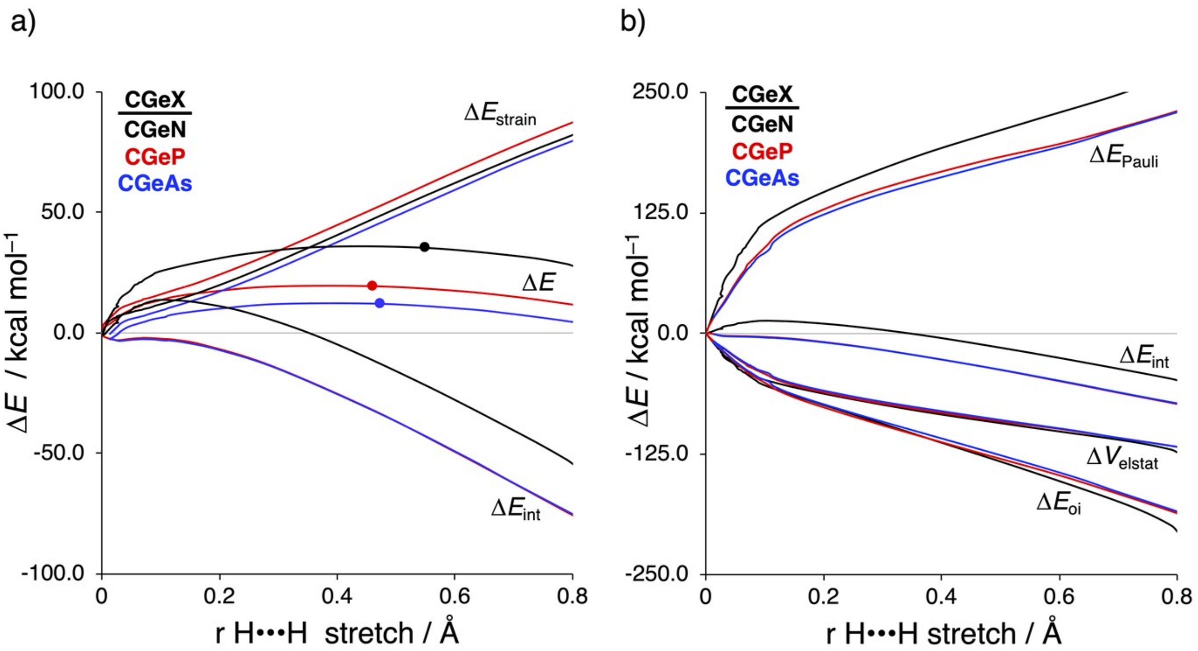

Fig. S8 (a) Activation strain analysis and (b) energy decomposition analysis of the H_2 bond activation by germylenes **CGeX** with variating Group 15 ligands (X = NMe_2, PMe_2, $AsMe_2$), where the transition states are indicated with a dot and the energies are projected onto the H•••H bond stretch of H_2, computed at ZORA-M06-2X/TZ2P//ZORA-BP86/TZ2P.

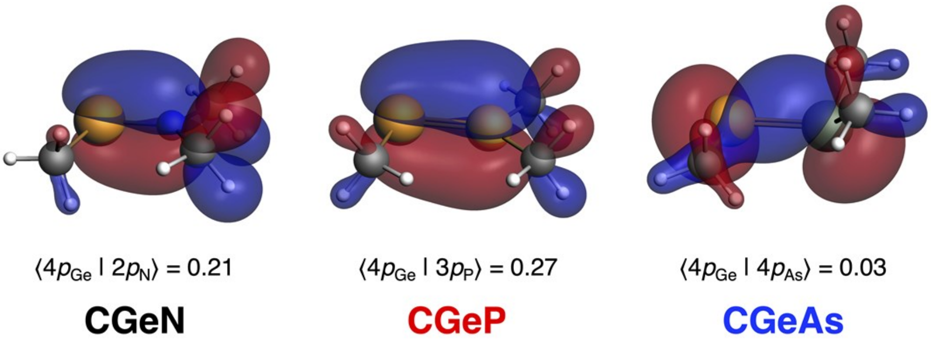

Fig. S9. Representations of the HOMO–1 orbital of the various **CGeX** metallylenes (X = N, P, As) in their equilibrium geometry (isovalue = 0.03 Bohr$^{-3/2}$) and the orbital overlap of the hyperconjugation interaction between the 4*p* atomic orbital of germanium and the *n*p atomic orbital of the ligand X, computed using ZORA-BP86/TZ2P.

Table S13. Activation strain and energy decomposition analyses (in kcal mol^{-1}) of for the H_2 activation by the synthesizable metallylene species **CGeX–4** (where X = N, P) and the three stages of simplified analogs.[a,b]

	$\Delta E^{\ddagger}$	$\Delta E^{\ddagger}_{strain}$	$\Delta E^{\ddagger}_{int}$	$\Delta V^{\ddagger}_{elstat}$	$\Delta E^{\ddagger}_{Pauli}$	$\Delta E^{\ddagger}_{oi}$
**CGeN–1**	38.1	57.7	–19.6	–97.3	219.1	–141.4
**CGeN–2**	37.9	59.0	–21.2	–99.7	221.6	–143.1
**CGeN–3**	45.1	60.5	–15.4	–106.6	247.2	–156.0
**CGeN–4**	40.4	55.9	–15.5	–107.1	246.0	–154.4
**CGeP–1**	18.4	51.5	–33.1	–87.7	176.0	–121.4
**CGeP–2**	20.3	49.5	–29.2	–90.8	187.2	–125.7
**CGeP–3**	22.6	52.7	–30.1	–93.8	192.2	–128.6
**CGeP–4**	25.3	49.1	–23.8	–90.4	191.1	–124.5

[a] Analyses on transition state structures. [b] Computed at ZORA-BP86/TZ2P.

Table S14. Cartesian coordinates (in Å), energies (in kcal mol^{-1}), and number of imaginary frequencies of all stationary points, computed at ZORA-BP86/TZ2P.

H_2
E (ZORA-BP86/TZ2P) = −155.28
E (ZORA-M06-2x/TZ2P//ZORA-BP86/TZ2P) = −210.93
H = −147.05
G = −156.34
N_{imag} = 0

1.H	0.000000	0.000000	-0.374806
2.H	0.000000	0.000000	0.374806

HCN
E = −451.10
H = −438.02
G = −453.39
N_{imag} = 0

1.C	-2.354095	2.736579	1.278860
2.N	-2.107630	2.682526	2.407137
3.H	-2.583143	2.786812	0.230320

CO_2
E = −529.71
H = −520.28
G = −353.64
N_{imag} = 0

1.C	-0.000000	0.000000	0.000000
2.O	-0.000000	0.000000	-1.170759
3.O	0.000000	-0.000000	1.170759

H_2O
E = −326.15
H = −310.82
G = −324.28
N_{imag} = 0

1.O	0.000000	0.000000	0.593104
2.H	0.000000	0.766340	-0.003468
3.H	0.000000	-0.766340	-0.003468

NH_3
E = −446.13
H = −422.86
G = −436.59
N_{imag} = 0

1.N	-0.884672	2.207237	1.049935
2.H	-1.473997	2.414402	1.858608
3.H	-1.454393	1.644247	0.415406
4.H	-0.138875	1.595223	1.386679

PH_3
E = −335.25
H = −338.27
G = −353.26
N_{imag} = 0

1.P	0.000000	0.000000	-0.592732
2.H	-0.595907	1.032141	0.197577
3.H	-0.595907	-1.032141	0.197577
4.H	1.191814	0.000000	0.197577

CH_4
E = –548.53
H = –518.89
G = –532.16
N_{imag} = 0

1.C	-0.000000	-0.000000	-0.000000
2.H	0.632203	-0.632203	-0.632203
3.H	-0.632203	0.632203	-0.632203
4.H	0.632203	0.632203	0.632203
5.H	-0.632203	-0.632203	0.632203

BF_3
E = –534.00
H = –523.70
G = –541.91
N_{imag} = 0

1.B	0.000000	0.000000	0.000000
2.F	0.661937	-1.146508	0.000000
3.F	0.661937	1.146508	0.000000
4.F	-1.323873	0.000000	0.000000

ethane
E = –922.58
H = –874.36
G = –890.63
N_{imag} = 0

1.C	0.000000	0.000000	0.765033
2.C	0.000000	0.000000	-0.765033
3.H	-0.885551	0.511273	1.165706
4.H	0.885551	0.511273	1.165706
5.H	0.000000	-1.022546	1.165706
6.H	0.000000	1.022546	-1.165706
7.H	-0.885551	-0.511273	-1.165706
8.H	0.885551	-0.511273	-1.165706

ethylene
E = –728.79
H = –695.22
G = –710.84
N_{imag} = 0

1.C	-0.000000	-0.000000	0.665633
2.C	-0.000000	0.000000	-0.665633
3.H	-0.000000	0.927834	-1.238514
4.H	-0.000000	0.927833	1.238514
5.H	-0.000000	-0.927834	1.238514
6.H	-0.000000	-0.927833	-1.238514

acetylene
E = –523.31
H = –504.56
G = –519.28
N_{imag} = 0

1.C	-0.000000	-0.468206	0.602373
2.C	-0.000000	-0.468501	-0.602377
3.H	-0.000000	-0.467944	1.672064
4.H	0.000000	-0.468763	-1.672067

H_3C–C–NMe_2 (CCN)
E (ZORA-BP86/TZ2P) = −1714.89
E (ZORA-M06-2x/TZ2P//ZORA-BP86/TZ2P) = −2370.30
H = −1634.95
G = −1658.94
N_{imag} = 0

1.C	-0.067111	0.507264	0.574390
2.N	-1.306614	0.589408	0.150617
3.H	0.765881	0.331836	-1.525202
4.H	-1.915646	0.739040	2.131706
5.H	-2.543872	-0.276938	-1.343093
6.C	-1.847202	0.565089	-1.235789
7.H	-2.395607	1.496015	-1.432002
8.H	-1.041733	0.461172	-1.964551
9.C	-2.382068	0.729178	1.144013
10.H	-2.936711	1.663744	0.972516
11.H	-3.086185	-0.112072	1.060714
12.C	1.015768	0.366506	-0.446627
13.H	1.727733	1.190421	-0.284383
14.H	1.586669	-0.540855	-0.196106

H_3C–C–PMe_2 (CCP)
E (ZORA-BP86/TZ2P)= −1631.86
H = −1555.21
G = −1581.71
N_{imag} = 0

1.C	-0.821473	-0.206748	-0.179582
2.P	-1.594353	0.721774	0.901770
3.H	-0.506536	-2.262793	-0.581641
4.H	0.194404	2.298764	0.928769
5.H	-3.055725	0.596966	2.889325
6.C	-3.220641	0.637548	1.804545
7.H	-3.836193	1.515678	1.568827
8.H	-3.749996	-0.267820	1.487519
9.C	-0.761101	2.241590	1.458990
10.H	-1.367464	3.126648	1.227312
11.H	-0.582939	2.214546	2.541702
12.C	-1.266215	-1.492182	-0.791538
13.H	-2.250272	-1.898889	-0.499521
14.H	-1.256619	-1.383901	-1.888329

H_3C–C–$AsMe_2$ (CCAs)
E = −1588.19
H = −1512.85
G = −1540.54
N_{imag} = 0

1.C	-0.795235	-0.158208	-0.389197
2.As	-1.485918	0.622031	1.088395
3.H	-0.737114	-2.202050	-1.030367
4.H	0.118310	2.510821	0.728895
5.H	-3.550087	0.944773	2.608497
6.C	-3.444086	0.702919	1.545945
7.H	-3.928582	1.468078	0.930477
8.H	-3.884063	-0.278790	1.345071
9.C	-0.882535	2.489691	1.169488
10.H	-1.561443	3.128296	0.594398
11.H	-0.847641	2.823173	2.211006
12.C	-1.454006	-1.367049	-0.960896
13.H	-2.401376	-1.739625	-0.531400
14.H	-1.650353	-1.100965	-2.017800

H_3C–Si–NMe_2 (CSiN)
E (ZORA-BP86/TZ2P) = –1656.37
E (ZORA-M06-2x/TZ2P//ZORA-BP86/TZ2P) = –2292.85
H = –1578.80
G = –1603.72
N_{imag} = 0

1.Si	0.105851	0.505679	0.822442
2.N	-1.488208	0.603658	0.137644
3.H	1.099774	1.189666	-1.432125
4.H	-2.269042	0.776045	2.084807
5.H	-2.604134	-0.280055	-1.431265
6.C	-1.915352	0.563887	-1.257753
7.H	-2.453037	1.488403	-1.527776
8.H	-1.056522	0.454036	-1.925813
9.C	-2.625903	0.749889	1.047467
10.H	-3.178953	1.681348	0.841356
11.H	-3.329490	-0.092081	0.937901
12.C	1.191935	0.324892	-0.756071
13.H	2.246450	0.250020	-0.457009
14.H	0.946547	-0.580196	-1.334149

H_3C–Si–PMe_2 (CSiP)
E = –1571.76
H = –1497.06
G = –1525.38
N_{imag} = 0

1.Si	-0.452357	-0.261511	-0.433582
2.P	-1.607499	0.841623	0.994233
3.H	-1.130593	-2.671231	-0.488370
4.H	0.033772	2.594400	1.227149
5.H	-3.154159	0.487540	2.869925
6.C	-3.255736	0.569800	1.780107
7.H	-3.936627	1.397500	1.543273
8.H	-3.686646	-0.361448	1.395481
9.C	-0.947695	2.411732	1.680432
10.H	-1.608333	3.255748	1.443933
11.H	-0.827204	2.348240	2.769560
12.C	-1.668717	-1.741478	-0.727795
13.H	-2.617414	-1.743371	-0.178205
14.H	-1.894553	-1.782171	-1.804181

H_3C–Si–$AsMe_2$ (CSiAs)
E = –1541.95
H = –1468.04
G = –1497.69
N_{imag} = 0

1.Si	0.405376	0.528055	0.818837
2.As	-1.666990	-0.313258	0.145511
3.H	1.228340	1.334849	-1.377155
4.H	-2.729482	0.514262	2.309636
5.H	-3.148981	-0.345704	-1.948909
6.C	-2.242417	0.212141	-1.691966
7.H	-2.439545	1.288063	-1.737383
8.H	-1.446571	-0.049139	-2.396441
9.C	-3.065050	0.545835	1.267691
10.H	-3.225368	1.585863	0.965337
11.H	-3.998834	-0.017681	1.171489
12.C	1.332439	0.363825	-0.860412
13.H	2.406639	0.202999	-0.698104
14.H	0.958449	-0.409388	-1.544630

H_3C–Ge–NMe_2 (CGeN; CGeN-1)

E (ZORA-BP86/TZ2P)= −1632.40
E (ZORA-M06-2x/TZ2P//ZORA-BP86/TZ2P) = −2272.93
H = −1555.35
G = −1581.34
N_{imag} = 0

1.Ge	0.138978	0.522727	0.848850
2.N	-1.564988	0.591851	0.111412
3.H	1.094414	1.182133	-1.522750
4.H	-2.359964	0.778210	2.050325
5.H	-2.640038	-0.331737	-1.462241
6.C	-1.966387	0.525566	-1.284838
7.H	-2.515004	1.437263	-1.581608
8.H	-1.094306	0.420064	-1.937069
9.C	-2.704894	0.733454	1.009173
10.H	-3.274587	1.653734	0.790637
11.H	-3.400726	-0.118033	0.909000
12.C	1.225052	0.320605	-0.852056
13.H	2.286575	0.254150	-0.580877
14.H	0.952277	-0.591179	-1.402758

H_3C–Ge–PMe_2 (CGeP; CGeP-1)

E (ZORA-BP86/TZ2P)= −1556.56
E (ZORA-M06-2x/TZ2P//ZORA-BP86/TZ2P) = −2154.03
H = −1482.17
G = −1511.92
N_{imag} = 0

1.Ge	-1.904179	2.090232	0.017401
2.P	-0.981226	0.075616	0.239923
3.H	-1.581316	2.875084	-2.431158
4.H	-2.284163	-0.527653	2.184727
5.H	1.158504	-1.093007	-0.070665
6.C	0.304166	-0.844248	-0.713834
7.H	-0.112489	-1.770105	-1.131664
8.H	0.656832	-0.214379	-1.538294
9.C	-1.515647	-1.044153	1.597446
10.H	-1.942963	-1.973981	1.199651
11.H	-0.676028	-1.293782	2.259365
12.C	-0.854184	2.672958	-1.632584
13.H	-0.356284	3.621238	-1.387985
14.H	-0.106270	1.965578	-2.004885

H_3C–Ge–$AsMe_2$ (CGeAs)

E (ZORA-BP86/TZ2P)= −1528.95
E (ZORA-M06-2x/TZ2P//ZORA-BP86/TZ2P) = −2130.72
H = −1455.31
G = −1486.00
N_{imag} = 0

1.Ge	-1.922375	2.078134	-0.029030
2.As	-0.477345	0.222616	0.629735
3.H	-1.171187	1.949446	-2.503425
4.H	-2.274771	-0.572320	2.268710
5.H	0.781618	-1.615710	-0.643428
6.C	0.108021	-0.817404	-0.973488
7.H	-0.751361	-1.249854	-1.496109
8.H	0.650927	-0.145508	-1.645809
9.C	-1.701933	-1.094064	1.494445
10.H	-2.389488	-1.537540	0.766828
11.H	-1.101466	-1.881412	1.962641
12.C	-0.871152	2.614267	-1.677638
13.H	-1.109770	3.646131	-1.961199

14.H	0.214311	2.515096	-1.548514

H_3C–Sn–NMe_2 (CSnN)

E (ZORA-BP86/TZ2P)= –1610.76

E (ZORA-M06-2x/TZ2P//ZORA-BP86/TZ2P) = –2246.31

H = –1532.22

G = –1561.26

N_{imag} = 0

1.Sn	-1.936124	0.498771	1.122555
2.N	-1.368005	1.076200	-0.779544
3.H	0.432915	-1.021997	1.136783
4.H	-2.669171	2.732899	-0.697095
5.H	-0.886288	0.121676	-2.610889
6.C	-0.411546	0.428860	-1.660125
7.H	0.418573	1.111704	-1.921528
8.H	0.014608	-0.462243	-1.187121
9.C	-1.951092	2.264739	-1.383823
10.H	-1.176219	3.013332	-1.632854
11.H	-2.482136	2.022222	-2.322975
12.C	-0.625626	-1.298655	1.239458
13.H	-0.765543	-1.784369	2.213602
14.H	-0.875799	-2.021556	0.450529

H_3C–Sn–PMe_2 (CSnP)

E = –1538.80

H = –1464.74

G = –1495.59

N_{imag} = 0

1.Sn	-1.981609	0.402826	1.362029
2.P	-1.758331	0.803350	-1.057744
3.H	0.468994	-0.955286	1.423368
4.H	-3.101505	2.828321	-1.098935
5.H	-0.706825	0.077510	-3.157718
6.C	-0.352284	0.274094	-2.137166
7.H	0.432463	1.041145	-2.172660
8.H	0.076789	-0.650001	-1.733408
9.C	-2.318411	2.421686	-1.750015
10.H	-1.494847	3.145739	-1.804136
11.H	-2.739498	2.286353	-2.754956
12.C	-0.561461	-1.327955	1.351148
13.H	-0.770391	-1.960131	2.222191
14.H	-0.658842	-1.932059	0.441614

H_3C–Sn–$AsMe_2$ (CSnAs)

E = –1514.06

H = –1440.67

G = –1472.32

N_{imag} = 0

1.Sn	-2.080918	0.319468	1.341314
2.As	-2.133205	0.490418	-1.318462
3.H	0.362510	-1.003282	1.096982
4.H	-3.375664	2.734312	-1.163483
5.H	-0.219965	0.429928	-3.027666
6.C	-0.232064	0.345746	-1.935120
7.H	0.396642	1.129598	-1.500403
8.H	0.153289	-0.638199	-1.648888
9.C	-2.434644	2.443127	-1.642923
10.H	-1.618347	3.055140	-1.245563
11.H	-2.521392	2.605762	-2.723109
12.C	-0.638200	-1.377689	1.357346

13.H	-0.597857	-1.840145	2.349902
14.H	-0.919600	-2.135455	0.614143

CGeN-2

E = −3633.48
H = −3505.04
G = −3540.38
N_{imag} = 0

1.Ge	0.104160	-0.105316	1.464780
2.N	-1.390550	0.965294	1.107178
3.H	-5.164997	3.402414	1.998760
4.H	-3.220208	2.818844	0.586220
5.H	-1.396154	1.482465	0.227444
6.H	2.861881	0.564790	-3.727313
7.H	-5.556163	2.210726	4.156659
8.H	-3.974517	0.416250	4.863987
9.C	-2.487273	1.280576	1.917313
10.C	-3.388216	2.289098	1.525891
11.C	-4.481435	2.618177	2.324523
12.C	-4.701602	1.953726	3.532285
13.C	-3.812494	0.948797	3.926625
14.C	-2.722406	0.608620	3.131945
15.H	-2.044016	-0.185124	3.446094
16.C	0.936319	0.153396	-0.345952
17.C	0.245842	0.367163	-1.552525
18.C	0.930367	0.513051	-2.760034
19.C	2.326679	0.449655	-2.784614
20.C	3.032404	0.226005	-1.600882
21.C	2.339199	0.067390	-0.398807
22.H	4.121333	0.168759	-1.616418
23.H	0.376089	0.670714	-3.686122
24.H	-0.846647	0.399866	-1.563560
25.H	2.905866	-0.121761	0.517399

CGeN-3

E = −6753.59
H = −6522.23
G = −6568.09
N_{imag} = 0

1.Ge	-0.411040	-0.389501	0.913066
2.N	-1.333984	1.252416	0.910256
3.H	-4.120531	4.545133	2.449894
4.C	-0.217512	-3.001865	-2.455579
5.H	-1.048156	1.955371	0.230164
6.H	2.973970	0.728850	-3.855348
7.H	-5.098626	3.054525	4.198059
8.H	-4.288259	0.701580	4.383582
9.C	-2.322341	1.714709	1.782647
10.C	-2.789774	3.040370	1.686744
11.C	-3.777943	3.514533	2.546833
12.C	-4.326109	2.683617	3.525848
13.C	-3.869187	1.365810	3.627154
14.C	-2.885351	0.882102	2.770116
15.H	-3.804548	-2.216012	-1.202523
16.C	0.732856	0.078045	-0.716046
17.C	0.550940	-0.672757	-1.899612
18.C	1.366553	-0.439389	-3.019300
19.C	2.353160	0.542183	-2.979035
20.C	2.554287	1.277976	-1.812716
21.C	1.767653	1.040508	-0.673556

22.H	3.351116	2.021265	-1.767439
23.H	1.202263	-1.012437	-3.932573
24.C	2.067416	1.784492	0.579377
25.C	2.239452	1.098018	1.794424
26.C	2.511586	1.787034	2.977139
27.C	2.625471	3.177768	2.966347
28.C	2.473840	3.871719	1.763922
29.C	2.201360	3.182159	0.582273
30.H	-0.951042	-4.985804	-2.854914
31.H	2.646168	1.233683	3.906323
32.H	2.835391	3.718194	3.888907
33.H	2.560606	4.958250	1.747176
34.H	-3.254033	-4.496344	-2.042931
35.C	-0.504646	-1.715975	-1.965778
36.C	-1.813592	-1.451770	-1.519586
37.C	-2.796048	-2.446368	-1.545094
38.C	-2.490313	-3.719341	-2.022755
39.C	-1.198180	-3.991492	-2.482571
40.H	0.794174	-3.229331	-2.791819
41.H	-2.364723	3.700513	0.928253
42.H	-2.545165	-0.150218	2.858901
43.H	2.207381	0.006783	1.802386
44.H	2.068317	3.732057	-0.349920
45.H	-2.081831	-0.439519	-1.214251

CGeN-4

E = −13482.33
H = −12927.34
G = −13008.72
N_{imag} = 0

1.Ge	-0.182114	-0.007834	1.096424
2.N	-1.697976	1.091465	0.969822
3.H	-4.237999	4.087875	3.347745
4.H	3.568187	-0.245104	4.005507
5.H	-1.684798	1.786592	0.227178
6.H	3.329882	0.019642	-3.694483
7.H	-5.335450	2.305185	4.695789
8.H	-4.805726	-0.063167	4.208258
9.C	-2.698991	1.370755	1.939521
10.C	-3.043934	2.736344	2.188671
11.C	-3.991659	3.039895	3.169242
12.C	-4.607117	2.049857	3.926659
13.C	-4.295435	0.727474	3.655318
14.C	-3.378253	0.344857	2.661320
15.H	3.011523	-1.747554	3.261869
16.C	0.819071	0.293568	-0.687122
17.C	0.544190	-0.549355	-1.792293
18.C	1.437063	-0.615274	-2.874908
19.C	2.632217	0.096324	-2.860369
20.C	2.934389	0.895888	-1.762231
21.C	2.043253	1.013846	-0.684586
22.H	3.875938	1.444610	-1.733412
23.H	1.193597	-1.258167	-3.721697
24.C	2.430649	1.924858	0.447922
25.C	2.869322	1.382799	1.687499
26.C	3.182207	2.258995	2.735216
27.C	3.117894	3.638273	2.565556
28.C	2.768448	4.163674	1.326608
29.C	2.426416	3.331963	0.252755
30.H	-1.507965	-4.725990	-1.357309
31.H	3.505707	1.855514	3.693716

32.H	3.365569	4.303433	3.393154
33.H	2.763141	5.245166	1.188785
34.H	-3.600766	-3.944771	-2.417921
35.C	-0.653102	-1.457488	-1.870645
36.C	-1.854061	-1.008409	-2.473683
37.C	-2.899816	-1.920086	-2.658522
38.C	-2.776441	-3.248488	-2.261281
39.C	-1.598442	-3.683744	-1.664593
40.C	-0.522620	-2.809392	-1.462222
41.H	-3.825985	-1.588477	-3.127851
42.C	0.767242	-3.353820	-0.854674
43.H	1.432277	-2.500187	-0.665727
44.C	0.532315	-4.061448	0.490730
45.H	0.043114	-3.394414	1.212277
46.H	1.489704	-4.385854	0.921175
47.H	-0.096599	-4.955064	0.376107
48.C	1.491550	-4.287260	-1.843090
49.H	1.718953	-3.771694	-2.784891
50.H	2.436985	-4.646050	-1.412791
51.H	0.873775	-5.164662	-2.080978
52.C	-2.006022	0.421209	-2.980632
53.H	-1.211365	1.019096	-2.512226
54.C	-1.791843	0.487096	-4.505489
55.H	-0.801727	0.107079	-4.786884
56.H	-1.874561	1.522948	-4.863705
57.H	-2.546719	-0.115513	-5.030251
58.C	-3.353942	1.052934	-2.597735
59.H	-3.533173	0.990966	-1.516942
60.H	-3.372569	2.111411	-2.890975
61.H	-4.194366	0.561606	-3.106995
62.C	2.149827	3.971143	-1.107724
63.H	1.748286	3.193056	-1.769301
64.C	1.119776	5.110461	-1.063738
65.H	0.161118	4.770740	-0.658477
66.H	0.944516	5.495529	-2.077825
67.H	1.466539	5.953443	-0.450053
68.C	3.463868	4.483001	-1.733044
69.H	4.214091	3.686842	-1.815720
70.H	3.280351	4.886241	-2.738795
71.H	3.899118	5.285501	-1.120814
72.C	3.166142	-0.107326	1.850330
73.H	2.531834	-0.660832	1.143697
74.C	4.630533	-0.385824	1.450456
75.H	4.830575	-0.067584	0.419681
76.H	4.852820	-1.459425	1.526955
77.H	5.321068	0.154684	2.113112
78.C	2.880260	-0.657336	3.254559
79.H	1.852434	-0.438183	3.570969
80.C	-3.300278	-1.176854	2.469289
81.H	-4.276940	-1.520292	2.845560
82.C	-3.236991	-1.696029	1.029614
83.H	-3.941799	-1.163186	0.379698
84.H	-3.502175	-2.761763	1.017664
85.H	-2.243880	-1.612520	0.572837
86.C	-2.256469	-1.852747	3.379882
87.H	-2.382920	-1.538767	4.424589
88.H	-2.367177	-2.945883	3.334081
89.H	-1.232623	-1.600497	3.073323
90.C	-2.401385	3.944230	1.505718
91.H	-2.971644	4.802468	1.892798
92.C	-0.948014	4.164263	1.958998

93.H	-0.888006	4.183453	3.054544
94.H	-0.568461	5.123588	1.582719
95.H	-0.275546	3.375628	1.601807
96.C	-2.570435	4.023261	-0.024491
97.H	-3.595671	3.771607	-0.323989
98.H	-2.360223	5.045962	-0.367031
99.H	-1.885223	3.370452	-0.584904

CGeP-2
E = −1737.68
H = −2628.30
G = −2662.84
N_{imag} = 0

1.Ge	-0.053122	0.247029	1.114028
2.P	-1.671666	1.575293	0.356102
3.C	3.292587	1.022711	-1.453297
4.C	2.406128	0.948892	-0.375884
5.H	4.294903	1.427006	-1.305886
6.H	3.590388	0.623927	-3.556290
7.H	1.303694	-0.311609	-3.872388
8.H	-4.085060	1.517436	0.821977
9.H	-3.307424	2.928836	1.592511
10.H	-0.957592	2.421491	-1.798552
11.H	-1.982190	3.619980	-0.957127
12.H	-2.731722	2.213250	-1.768501
13.C	-1.861072	2.554662	-1.193423
14.H	-3.098256	1.301562	2.291339
15.C	-3.189429	1.863597	1.354349
16.C	1.104669	0.444159	-0.537893
17.C	0.729513	-0.014130	-1.812440
18.C	1.615854	0.049430	-2.891381
19.C	2.898490	0.573125	-2.715443
20.H	2.739297	1.296579	0.605138
21.H	-0.267396	-0.431017	-1.967884

CGeP-3
E = −5857.57
H = −5644.11
G = −5692.62
N_{imag} = 0

1.Ge	-0.329327	-0.037330	0.759524
2.P	-1.473542	1.945645	0.447027
3.C	-1.300014	-0.737426	-3.349039
4.C	-0.756192	-2.067789	-1.411038
5.H	-3.098339	-1.288418	-4.397285
6.H	3.432631	1.156148	-3.702491
7.C	-2.441839	-1.510030	-3.555582
8.C	-2.753169	-2.558463	-2.685008
9.H	3.398300	3.009915	4.387571
10.H	3.369189	4.539962	2.422409
11.H	-3.647519	-3.159308	-2.848016
12.C	-0.440213	-0.995114	-2.267084
13.C	-1.607055	2.613858	-1.277998
14.C	-1.905092	-2.837192	-1.613913
15.C	-3.238215	1.705530	0.971752
16.C	1.033863	0.325885	-0.727083
17.C	0.768512	-0.165427	-2.024465
18.C	1.629811	0.142184	-3.089385
19.C	2.759849	0.928761	-2.875687
20.C	3.037619	1.407507	-1.596923
21.C	2.190579	1.107128	-0.516514

22.H	3.932136	2.006492	-1.421702
23.H	1.418393	-0.253722	-4.083565
24.C	2.525511	1.633485	0.834753
25.C	2.552905	0.780488	1.952186
26.C	2.865191	1.271658	3.220961
27.C	3.157775	2.624640	3.396938
28.C	3.144665	3.481002	2.293616
29.C	2.835170	2.989601	1.025761
30.H	-2.124175	-3.666912	-0.942149
31.H	2.888440	0.591271	4.072163
32.H	-1.075057	0.091216	-4.020652
33.H	-0.058280	-2.343091	-0.618580
34.H	-0.602154	2.663190	-1.712201
35.H	-2.025001	3.628883	-1.249937
36.H	-2.239489	1.980637	-1.913044
37.H	-3.250971	1.157559	1.921974
38.H	-3.820773	1.142927	0.231214
39.H	-3.715349	2.680759	1.134045
40.H	2.362303	-0.284215	1.816570
41.H	2.807354	3.667456	0.172456

CGeP-4
E = −12457.90
H = −11930.70
G = −12015.98
N_{imag} = 0

1.Ge	-0.391406	0.312008	0.480989
2.P	-1.687083	2.213920	0.449537
3.C	3.001528	-0.692961	1.710266
4.H	4.110498	5.078121	-0.104181
5.H	3.404335	5.143280	-1.735808
6.H	4.128770	0.042882	-3.331328
7.H	2.548501	-1.007033	0.760360
8.C	4.505253	-1.024070	1.628087
9.H	4.982407	-0.505195	0.787061
10.H	4.656369	-2.104408	1.493990
11.H	5.021564	-0.719391	2.549402
12.C	2.329005	-1.500007	2.831646
13.H	1.258725	-1.266739	2.901320
14.H	2.433640	-2.575823	2.635532
15.H	2.787107	-1.304049	3.810775
16.C	1.036215	0.423532	-0.973596
17.C	0.940286	-0.350280	-2.157562
18.C	2.062185	-0.473287	-2.991406
19.C	3.267686	0.147797	-2.671199
20.C	3.378958	0.866787	-1.483309
21.C	2.282544	0.992633	-0.617409
22.H	4.334776	1.310100	-1.201887
23.H	1.993685	-1.082803	-3.892043
24.C	2.494960	1.638743	0.728171
25.C	2.802254	0.813917	1.844537
26.C	3.021742	1.413433	3.091710
27.C	2.964544	2.793158	3.246693
28.C	2.696945	3.599259	2.144875
29.C	2.462272	3.047814	0.879972
30.H	-2.178207	-3.755240	-1.380219
31.H	3.253837	0.788511	3.953689
32.H	3.138420	3.242745	4.224733
33.H	2.670182	4.680772	2.273068
34.H	-3.665679	-2.991155	-3.199564
35.C	-0.320802	-1.102879	-2.493090

36.C	-1.133313	-0.726580	-3.596287
37.C	-2.329097	-1.419845	-3.825641
38.C	-2.723398	-2.475720	-3.011945
39.C	-1.891230	-2.892502	-1.979528
40.C	-0.679002	-2.241338	-1.714627
41.H	-2.964062	-1.128462	-4.661844
42.C	0.293493	-2.877112	-0.721543
43.H	0.954425	-2.088663	-0.337601
44.C	-0.381157	-3.539589	0.487587
45.H	-1.048349	-2.840183	1.007722
46.H	0.381944	-3.873482	1.202857
47.H	-0.963311	-4.426341	0.201343
48.C	1.187612	-3.893560	-1.462907
49.H	1.737702	-3.416885	-2.283805
50.H	1.918714	-4.338306	-0.773295
51.H	0.579660	-4.705175	-1.887094
52.C	-0.716200	0.348900	-4.596210
53.H	0.056807	0.968513	-4.120457
54.C	-0.092968	-0.297578	-5.851997
55.H	0.759307	-0.941618	-5.606213
56.H	0.253626	0.476062	-6.551470
57.H	-0.837323	-0.917105	-6.372055
58.C	-1.866998	1.271704	-5.029639
59.H	-2.390762	1.704268	-4.170827
60.H	-1.475770	2.095674	-5.641575
61.H	-2.606031	0.737618	-5.642363
62.C	2.233015	3.977106	-0.305811
63.H	1.748552	3.384913	-1.095351
64.C	1.311799	5.159896	0.030810
65.H	0.364793	4.814078	0.464498
66.H	1.091828	5.739978	-0.876093
67.H	1.779833	5.851973	0.744072
68.C	3.573697	4.493957	-0.865167
69.H	4.225841	3.669660	-1.178166
70.Si	-3.378678	1.663541	1.862905
71.C	-4.450846	3.182450	2.206801
72.H	-5.207054	2.930316	2.965212
73.H	-3.853456	4.019143	2.591381
74.H	-4.985186	3.525918	1.311419
75.C	-4.472156	0.286257	1.173950
76.H	-4.940349	0.578355	0.225121
77.H	-5.272536	0.037036	1.886766
78.H	-3.883470	-0.621820	0.988995
79.C	-2.593707	1.091304	3.479789
80.H	-1.999483	1.893066	3.936821
81.H	-3.375632	0.791078	4.193062
82.H	-1.930515	0.231386	3.319027
83.Si	-2.428273	3.536511	-1.243182
84.C	-2.838584	5.257076	-0.578691
85.H	-3.177786	5.900242	-1.404995
86.H	-3.635653	5.229124	0.174308
87.H	-1.958320	5.728288	-0.123450
88.C	-1.050596	3.695905	-2.515873
89.H	-0.233931	4.308876	-2.116880
90.H	-1.429277	4.185799	-3.424324
91.H	-0.635744	2.721426	-2.796695
92.C	-3.992093	2.800279	-2.007633
93.H	-4.817954	2.797544	-1.283714
94.H	-4.311320	3.398998	-2.873919
95.H	-3.834097	1.766773	-2.339310

TS: CCN + H_2
E (ZORA-BP86/TZ2P)= -1857.99
E (ZORA-M06-2x/TZ2P//ZORA-BP86/TZ2P) = -2561.47
H = -1769.6
G = -1793.99
N_{imag} = 1,-905.513i cm^{-1}

1.C	0.272688	0.656304	-0.065180
2.N	-0.553095	0.818095	0.973757
3.H	1.011637	-0.619497	-1.594029
4.H	0.100318	2.781382	1.246347
5.H	-0.780724	-0.735149	2.399453
6.C	-1.352442	-0.245214	1.591878
7.H	-2.269024	0.181380	2.018166
8.H	-1.628659	-0.995968	0.847669
9.C	-0.307167	1.962033	1.844986
10.H	-1.238852	2.283903	2.326913
11.H	0.423778	1.692432	2.627983
12.H	1.455801	0.935302	0.157628
13.H	2.108982	0.270203	0.653536
14.C	0.113553	-0.527720	-0.971742
15.H	-0.061687	-1.503167	-0.491443
16.H	-0.742047	-0.333411	-1.637669

PC: CCN + H_2
E (ZORA-BP86/TZ2P)= -1928.48
E (ZORA-M06-2x/TZ2P//ZORA-BP86/TZ2P) = -2645.36
H = -1833.03
G = -1857.03
N_{imag} = 0

1.C	-0.395579	-0.412719	1.130879
2.N	-0.915704	0.879749	0.709849
3.H	0.249520	-0.282627	2.010483
4.H	-1.128866	0.353891	-1.305862
5.H	-1.764517	2.646686	1.392032
6.C	-1.546707	1.643488	1.790200
7.H	-2.647805	0.077366	2.871837
8.H	-0.788099	1.777163	2.576977
9.C	-1.732285	0.776526	-0.490477
10.H	-2.631743	0.134341	-0.375052
11.H	-2.065471	1.777166	-0.797879
12.H	0.213910	-0.840504	0.322725
13.H	-1.179743	-1.157565	1.385956
14.C	-2.826334	1.060570	2.416880
15.H	-3.190911	1.729698	3.208119
16.H	-3.631379	0.953148	1.678017

TS: CCP + H_2
E = -1778.64
H = -1693.98
G = -1720.49
N_{imag} = 1,-626.643i cm^{-1}

1.C	0.335678	0.596510	-0.270110
2.P	-0.848429	0.913209	0.914764
3.H	-0.407692	-0.299087	-2.021728
4.H	0.161722	3.034534	1.484732
5.H	-0.547543	-0.782352	2.685163
6.C	-1.373126	-0.482741	2.024719
7.H	-2.236652	-0.182623	2.632059
8.H	-1.668382	-1.338318	1.406040
9.C	-0.129011	2.150384	2.061391
10.H	-0.866891	2.443397	2.817336

11.H	0.759045	1.738230	2.562605
12.H	1.644406	0.765733	0.180818
13.H	2.149150	0.227168	0.655901
14.C	0.267240	-0.580236	-1.196766
15.H	1.249768	-0.760781	-1.654952
16.H	-0.104698	-1.533383	-0.784020

PC: CCP + H_2
E = −1867.05
H = −1693.98
G = −1720.49
N_{imag} = 0

1.C	0.945505	-0.672762	0.792126
2.P	-0.115252	0.532416	1.729827
3.H	0.923710	-0.524816	-0.296341
4.H	1.474329	2.333751	1.298341
5.H	-2.441289	1.035304	1.364213
6.C	-1.778024	0.286906	0.903337
7.H	-2.918205	0.190093	-0.949267
8.H	-2.139099	-0.687183	1.268388
9.C	0.457278	2.118288	0.946149
10.H	0.466980	2.088723	-0.152097
11.H	-0.188924	2.941756	1.276975
12.H	1.981960	-0.567306	1.137807
13.H	0.622750	-1.696696	1.021305
14.C	-1.881354	0.353647	-0.622683
15.H	-1.568443	1.331787	-1.010464
16.H	-1.262740	-0.412720	-1.107467

TS: CCAs + H_2
E = −1737.98
H = −1654.19
G = −1682.20
N_{imag} = 1,−468.259i cm^{-1}

1.C	1.227233	-0.815060	1.212627
2.As	-0.150421	0.544345	1.682639
3.H	1.438655	-0.810830	0.138600
4.H	1.510369	2.437755	1.232183
5.H	-3.233941	-0.338388	1.979508
6.C	-1.541947	-0.142949	0.534601
7.H	-3.220880	0.504571	-0.667823
8.H	-2.683268	-0.680217	1.479846
9.C	0.596821	2.126917	0.713442
10.H	0.829441	1.885362	-0.329484
11.H	-0.125187	2.949466	0.752937
12.H	2.138328	-0.592225	1.778457
13.H	0.837041	-1.793731	1.508228
14.C	-2.212399	0.827695	-0.368288
15.H	-2.227613	1.900413	-0.107944
16.H	-1.582674	0.749034	-1.279785

PC: CCAs + H_2
E = −1839.05
H = −1748.28
G = −1775.58
N_{imag} = 0

1.C	0.999901	-0.734274	0.710811
2.As	-0.108986	0.530498	1.783449
3.H	0.931175	-0.535372	-0.364661
4.H	1.508608	2.423693	1.199225
5.H	-2.534929	1.027590	1.295613

6.C	-1.871709	0.273746	0.847374
7.H	-2.928798	0.194135	-1.052282
8.H	-2.231745	-0.703759	1.199420
9.C	0.485768	2.202829	0.872598
10.H	0.468287	2.108249	-0.219040
11.H	-0.164779	3.029283	1.181816
12.H	2.042965	-0.629119	1.031185
13.H	0.673628	-1.760185	0.917931
14.C	-1.907043	0.352206	-0.675771
15.H	-1.575174	1.332380	-1.042378
16.H	-1.269577	-0.412005	-1.139322

TS: CSiN + H_2
E (ZORA-BP86/TZ2P) = −1787.59
E (ZORA-M06-2x/TZ2P//ZORA-BP86/TZ2P) = −2474.53
H = −1701.52
G = −1727.50
N_{imag} = 1,−1110.322i cm^{-1}

1.Si	0.519185	0.655168	-0.129052
2.N	-0.587394	0.852072	1.185468
3.H	0.857311	-0.995864	-1.940435
4.H	-0.155644	2.906258	1.349621
5.H	-0.774967	-0.490468	2.829574
6.C	-1.265861	-0.240823	1.869695
7.H	-2.314184	0.026163	2.087922
8.H	-1.269809	-1.142964	1.247941
9.C	-0.653405	2.111118	1.918541
10.H	-1.702774	2.409191	2.086932
11.H	-0.163789	2.042819	2.907836
12.H	1.994277	0.485215	0.269482
13.H	1.587072	-0.541805	0.550897
14.C	-0.029937	-0.639662	-1.399339
15.H	-0.541400	-1.508690	-0.969602
16.H	-0.702481	-0.167532	-2.126543

PC: CSiN + H_2
E (ZORA-BP86/TZ2P) = −1847.44
E (ZORA-M06-2x/TZ2P//ZORA-BP86/TZ2P) = −2542.57
H = −1758.90
G = −1727.50
N_{imag} = 0

1.C	-0.181225	-0.496582	0.909748
2.N	-0.944934	0.704414	0.608344
3.H	0.284106	-0.414245	1.900718
4.H	-0.736104	0.586330	-1.489785
5.H	-1.767176	3.086873	1.209173
6.Si	-1.516111	1.762004	1.856120
7.H	-2.961267	0.283383	3.274724
8.H	-0.413210	1.870064	2.860452
9.C	-1.523988	0.708736	-0.726265
10.H	-2.259645	-0.104621	-0.877550
11.H	-2.030670	1.663374	-0.919377
12.H	0.626284	-0.636762	0.170178
13.H	-0.801524	-1.413500	0.899198
14.C	-3.086597	1.248209	2.764685
15.H	-3.341861	1.999907	3.526416
16.H	-3.940492	1.162877	2.078869

TS: CSiP + H_2
E = −1718.52
H = −1635.40
G = −1663.13
N_{imag} = 1,−714.649 cm^{-1}

1.C	-0.133824	-0.655278	1.105084
2.P	-0.326843	1.086879	0.461954
3.H	0.439347	-0.632273	2.039832
4.H	-0.769650	0.208288	-1.781557
5.H	-0.980334	2.997861	2.692736
6.Si	-1.887046	2.077192	1.795129
7.H	-2.406213	-0.031222	3.186427
8.H	-0.548301	2.072447	2.823584
9.C	-1.386365	0.733750	-1.039918
10.H	-2.271355	0.119376	-0.826886
11.H	-1.710122	1.684754	-1.480044
12.H	0.435971	-1.237914	0.368036
13.H	-1.089438	-1.165060	1.285808
14.C	-2.860066	0.950155	3.007274
15.H	-2.970187	1.467874	3.970098
16.H	-3.870038	0.796528	2.605207

PC: CSiP + H_2
E = −1771.56
H = −1686.11
G = −1663.13
N_{imag} = 0

1.C	-0.088257	-0.644448	1.048239
2.P	-0.331682	1.088582	0.388844
3.H	0.564875	-0.606904	1.929066
4.H	-1.224289	0.239068	-1.729452
5.H	-1.979017	3.352534	1.607121
6.Si	-1.569995	1.987562	2.065636
7.H	-2.836523	0.028200	2.987790
8.H	-0.646825	2.158285	3.231510
9.C	-1.668418	0.776464	-0.881286
10.H	-2.512991	0.188502	-0.498303
11.H	-2.042198	1.737806	-1.255398
12.H	0.422021	-1.240864	0.280574
13.H	-1.024928	-1.149758	1.318421
14.C	-3.101216	1.025792	2.614319
15.H	-3.608954	1.565170	3.425752
16.H	-3.816901	0.906253	1.790748

TS: CSiAs + H_2
E = −1694.82
H = −1612.43
G = −1641.58
N_{imag} = 1,−747.128i cm^{-1}

1.C	0.355580	-0.522032	0.894614
2.As	-0.188257	1.343579	0.391831
3.H	1.031122	-0.469163	1.755290
4.H	-1.094067	0.326991	-1.777460
5.H	-1.789209	3.145558	1.443915
6.Si	-1.470053	1.783073	2.388289
7.H	-3.925287	2.069264	2.811855
8.H	-1.469231	3.358897	2.409717
9.C	-1.594704	0.812607	-0.930917
10.H	-2.327585	0.121081	-0.501316
11.H	-2.103860	1.715562	-1.284953
12.H	0.894338	-0.953939	0.042724

13.H	-0.502205	-1.158368	1.138350
14.C	-3.324765	1.282078	2.335653
15.H	-3.723484	1.094880	1.332244
16.H	-3.452656	0.370406	2.934015

PC: CSiAs + H_2

E = −1747.24
H = −1662.42
G = −1691.79
N_{imag} = 0

1.C	0.094927	-0.572082	1.029250
2.As	-0.149114	1.315957	0.396881
3.H	0.712412	-0.565787	1.934304
4.H	-1.096879	0.445873	-1.819652
5.H	-2.072254	3.412452	1.793236
6.Si	-1.594489	2.040720	2.157291
7.H	-3.659171	1.365264	3.369628
8.H	-0.748708	2.176607	3.385601
9.C	-1.572289	0.944448	-0.967175
10.H	-2.370714	0.306502	-0.573212
11.H	-1.994290	1.896475	-1.307864
12.H	0.627223	-1.121681	0.244445
13.H	-0.858944	-1.069018	1.236348
14.C	-3.081454	0.939193	2.537837
15.H	-3.749585	0.855854	1.671157
16.H	-2.767232	-0.071373	2.828656

TS: CGeN + H_2

E (ZORA-BP86/TZ2P)= −1749.64
E (ZORA-M06-2x/TZ2P//ZORA-BP86/TZ2P) = −2442.71
H = −1664.61
G = −1691.91
N_{imag} = 1,−1193.756i cm^{-1}

1.Ge	0.522288	0.680220	-0.159525
2.N	-0.772349	0.934537	1.158321
3.H	0.970415	-1.160697	-1.852571
4.H	-0.138871	2.912714	1.513142
5.H	-0.657888	-0.621668	2.630826
6.C	-1.328319	-0.226045	1.840380
7.H	-2.289168	0.042681	2.311679
8.H	-1.525951	-1.036027	1.128169
9.C	-0.539359	2.052000	2.064252
10.H	-1.490382	2.359879	2.530337
11.H	0.167257	1.809875	2.884712
12.H	2.008317	0.741440	0.341286
13.H	1.614264	-0.421986	0.764583
14.C	0.049034	-0.808024	-1.377276
15.H	-0.429426	-1.638666	-0.851714
16.H	-0.627290	-0.415908	-2.143163

PC: CGeN + H_2

E (ZORA-BP86/TZ2P)= −1800.60
E (ZORA-M06-2x/TZ2P//ZORA-BP86/TZ2P) = −2503.13
H = −1713.14
G = −1740.33
N_{imag} = 0

1.C	-0.162518	-0.444281	0.922896
2.N	-0.764269	0.829690	0.552850
3.H	0.403694	-0.340923	1.858477
4.H	-0.809801	0.456674	-1.516875
5.H	-1.879944	3.188312	1.309665

6.Ge	-1.556485	1.813827	1.926105
7.H	-2.982092	0.026970	3.110648
8.H	-0.477352	1.930781	3.019690
9.C	-1.494743	0.755425	-0.705279
10.H	-2.329890	0.023909	-0.697570
11.H	-1.906901	1.739832	-0.965338
12.H	0.545354	-0.763731	0.139334
13.H	-0.896605	-1.266791	1.054122
14.C	-3.197534	1.013637	2.684981
15.H	-3.584696	1.662321	3.480588
16.H	-3.967197	0.909100	1.911830

TS: CGeP + H_2
E (ZORA-BP86/TZ2P)= −1693.44
E (ZORA-M06-2x/TZ2P//ZORA-BP86/TZ2P) = −2345.22
H = −1611.06
G = −1639.93
N_{imag} = 1,-1102.001i cm^{-1}

1.C	-0.062228	-0.666378	1.051750
2.P	-0.195653	1.110178	0.500301
3.H	0.514394	-0.711301	1.983146
4.H	-0.697587	0.358745	-1.772678
5.H	-0.964154	3.005365	2.917472
6.Ge	-1.803111	2.041240	1.958352
7.H	-2.434559	-0.215907	3.162323
8.H	-0.451355	1.916899	3.041276
9.C	-1.289860	0.863153	-0.996634
10.H	-2.186685	0.261097	-0.799260
11.H	-1.594633	1.841992	-1.386345
12.H	0.483539	-1.226707	0.279411
13.H	-1.034191	-1.152086	1.209180
14.C	-2.873581	0.784720	3.110407
15.H	-2.935427	1.209552	4.118507
16.H	-3.883037	0.720329	2.690452

PC: CGeP + H_2
E (ZORA-BP86/TZ2P)= −1738.07
E (ZORA-M06-2x/TZ2P//ZORA-BP86/TZ2P) = −2398.52
H = −1653.39
G = −1682.60
N_{imag} = 0

1.C	-0.018166	-0.590838	1.021848
2.P	-0.279432	1.148590	0.390238
3.H	0.623842	-0.560995	1.911001
4.H	-1.124031	0.303817	-1.746551
5.H	-2.106903	3.393451	1.685413
6.Ge	-1.625604	1.988567	2.111757
7.H	-2.883965	-0.106917	2.929725
8.H	-0.733431	2.160714	3.361335
9.C	-1.592142	0.823653	-0.899794
10.H	-2.428379	0.213296	-0.534078
11.H	-1.981311	1.780297	-1.269989
12.H	0.509885	-1.164417	0.248211
13.H	-0.950731	-1.114656	1.269888
14.C	-3.198131	0.885851	2.589154
15.H	-3.747657	1.378187	3.400197
16.H	-3.866900	0.774838	1.728519

TS: CGeAs + H_2
E (ZORA-BP86/TZ2P)= −1671.17
E (ZORA-M06-2x/TZ2P//ZORA-BP86/TZ2P) = −2323.29
H = −1589.47
G = −1619.82
N_{imag} = 1,−1091.410i cm^{-1}

1.C	-0.031191	-0.741146	1.046927
2.As	-0.159025	1.155894	0.427873
3.H	0.570488	-0.772923	1.961349
4.H	-0.872758	0.273190	-1.868396
5.H	-1.027596	3.027700	2.964106
6.Ge	-1.863874	2.073004	1.985742
7.H	-2.381366	-0.235070	3.153832
8.H	-0.463015	1.943313	3.004165
9.C	-1.429024	0.800324	-1.083444
10.H	-2.284903	0.189347	-0.776801
11.H	-1.782934	1.758229	-1.479773
12.H	0.473563	-1.316486	0.260768
13.H	-1.016021	-1.180768	1.237816
14.C	-2.850055	0.753301	3.146658
15.H	-2.885832	1.148002	4.168217
16.H	-3.872519	0.669463	2.762726

PC: CGeAs + H_2
E (ZORA-BP86/TZ2P)= −1714.89
E (ZORA-M06-2x/TZ2P//ZORA-BP86/TZ2P) = −2375.67
H = −1630.82
G = −1661.48
N_{imag} = 0

1.C	-0.018527	-0.695082	1.051555
2.As	-0.215740	1.175143	0.358194
3.H	0.614425	-0.678388	1.945691
4.H	-1.224032	0.246691	-1.806148
5.H	-2.163165	3.416403	1.749934
6.Ge	-1.660033	2.011899	2.156860
7.H	-2.889523	-0.115430	2.929288
8.H	-0.791553	2.187058	3.424267
9.C	-1.670798	0.790866	-0.965709
10.H	-2.477374	0.189700	-0.532500
11.H	-2.074776	1.739531	-1.336172
12.H	0.482018	-1.288390	0.277285
13.H	-0.984438	-1.152636	1.290908
14.C	-3.218071	0.876097	2.599069
15.H	-3.786846	1.346970	3.409665
16.H	-3.872748	0.765456	1.727711

TS: CSnN + H_2
E (ZORA-BP86/TZ2P) = −1717.47
E (ZORA-M06-2x/TZ2P//ZORA-BP86/TZ2P) = −2402.45
H = −1633.56
G = −1661.84
N_{imag} = 1,−1175.515i cm^{-1}

1.C	-0.198100	-0.400389	0.958888
2.N	-0.608149	0.944616	0.572748
3.H	0.208452	-0.397748	1.978079
4.H	-0.199993	0.696835	-1.472319
5.H	-0.816827	3.298650	2.586188
6.Sn	-1.789363	2.015487	1.924073
7.H	-2.343135	-0.175030	3.525852
8.H	-0.332035	2.127648	3.212472
9.C	-1.041952	0.992060	-0.821384

10.H	-1.887828	0.314772	-1.060953
11.H	-1.335335	2.012940	-1.098970
12.H	0.599872	-0.744206	0.276211
13.H	-1.007004	-1.159089	0.909711
14.C	-2.869784	0.772173	3.385428
15.H	-2.909829	1.325844	4.328201
16.H	-3.879116	0.598566	3.003343

PC: CSnN + H_2
E (ZORA-BP86/TZ2P)= −1764.56
E (ZORA-M06-2x/TZ2P//ZORA-BP86/TZ2P) = −2461.22
H = −1678.78
G = −1707.47
N_{imag} = 0

1.C	0.004418	-0.414369	0.803536
2.N	-0.622115	0.852622	0.451949
3.H	0.561906	-0.320901	1.745941
4.H	-0.628109	0.503831	-1.623754
5.H	-1.897098	3.447078	1.279140
6.Sn	-1.526495	1.915644	1.978159
7.H	-3.090072	-0.013142	3.163311
8.H	-0.320834	2.026064	3.204879
9.C	-1.332405	0.776600	-0.817383
10.H	-2.149465	0.024225	-0.835749
11.H	-1.765161	1.752859	-1.076850
12.H	0.727948	-0.703333	0.020150
13.H	-0.708956	-1.259158	0.910114
14.C	-3.330487	0.974533	2.756090
15.H	-3.751374	1.599007	3.552436
16.H	-4.069552	0.867917	1.955073

TS: CSnP + H_2
E = −1665.12
H = −1583.74
G = −1613.91
N_{imag} = 1,−1154.080i cm^{-1}

1.C	-0.015557	-0.723152	0.973689
2.P	-0.110394	1.046069	0.385787
3.H	0.542282	-0.757934	1.916576
4.H	-0.607155	0.272301	-1.876804
5.H	-0.918572	3.183525	3.036343
6.Sn	-1.826747	2.081716	1.991797
7.H	-2.561008	-0.292908	3.266288
8.H	-0.352811	1.958650	3.212276
9.C	-1.201823	0.793595	-1.113312
10.H	-2.106112	0.203380	-0.917197
11.H	-1.492577	1.769438	-1.520596
12.H	0.539314	-1.301393	0.220736
13.H	-0.994866	-1.196682	1.119460
14.C	-3.007477	0.705074	3.254957
15.H	-3.025319	1.117359	4.268943
16.H	-4.024778	0.659597	2.855441

PC: CSnP + H_2
E = −1706.15
H = −1622.87
G = −1653.64
N_{imag} = 0

1.C	0.150204	-0.553705	0.913876
2.P	-0.120486	1.193189	0.298077
3.H	0.787094	-0.530378	1.806837

4.H	-0.933328	0.353999	-1.852627
5.H	-2.190082	3.639340	1.712629
6.Sn	-1.610765	2.073943	2.164243
7.H	-2.984016	-0.183716	2.950258
8.H	-0.654263	2.251579	3.593780
9.C	-1.419106	0.864013	-1.009401
10.H	-2.254361	0.242748	-0.661116
11.H	-1.812815	1.818595	-1.379968
12.H	0.688601	-1.111053	0.135018
13.H	-0.777212	-1.091782	1.149043
14.C	-3.322986	0.805852	2.626822
15.H	-3.896888	1.270608	3.436178
16.H	-3.965142	0.701584	1.746195

TS: CSnAs + H_2
E = −1644.33
H = −1563.56
G = −1595.19
N_{imag} = 1,-1142.060i cm^{-1}

1.C	-0.004364	-0.776343	0.995383
2.As	-0.089319	1.113635	0.342856
3.H	0.567903	-0.798993	1.928420
4.H	-0.772637	0.222189	-1.954892
5.H	-1.010462	3.221147	3.095196
6.Sn	-1.905429	2.116264	2.034387
7.H	-2.516812	-0.308085	3.284347
8.H	-0.376210	2.010359	3.186810
9.C	-1.341029	0.759046	-1.185065
10.H	-2.206441	0.155805	-0.891570
11.H	-1.680617	1.715412	-1.596999
12.H	0.520103	-1.365844	0.232716
13.H	-0.997715	-1.206107	1.161187
14.C	-2.990702	0.676810	3.314934
15.H	-2.978271	1.064557	4.338717
16.H	-4.022321	0.609693	2.956914

PC: CSnAs + H_2
E = −1684.57
H = −1601.86
G = −1634.01
N_{imag} = 0

1.C	0.206343	-0.584049	0.957065
2.As	-0.025716	1.302990	0.313326
3.H	0.818685	-0.578865	1.865448
4.H	-0.943143	0.402284	-1.901345
5.H	-2.298906	3.658674	1.828154
6.Sn	-1.667969	2.098454	2.233059
7.H	-2.980566	-0.233322	2.893482
8.H	-0.777718	2.265885	3.708043
9.C	-1.433303	0.912486	-1.063533
10.H	-2.236189	0.278111	-0.673654
11.H	-1.852022	1.858250	-1.424619
12.H	0.741716	-1.135926	0.175204
13.H	-0.749559	-1.078614	1.158782
14.C	-3.345730	0.754491	2.594248
15.H	-3.961529	1.169098	3.400092
16.H	-3.954913	0.659577	1.689522

TS: CGeN-2 + H_2
E = −3750.93
H = −3615.44
G = −3649.93
N_{imag} = 1,−1172.063i cm^{-1}

1.Ge	0.507018	-0.696381	0.513246
2.N	-1.039487	-0.532370	1.549803
3.H	-3.334706	-0.546115	5.504719
4.H	-0.215828	-1.937869	-0.502570
5.H	-1.934075	-0.741754	1.120473
6.H	0.401119	3.113001	-3.934591
7.H	-1.263013	-0.151609	6.843935
8.H	0.927264	0.042719	5.661127
9.C	-1.076460	-0.454152	2.941759
10.C	-2.306784	-0.566669	3.620575
11.C	-2.368566	-0.455773	5.007337
12.C	-1.212121	-0.235309	5.759438
13.C	0.011506	-0.127371	5.094311
14.C	0.083745	-0.235350	3.707451
15.H	0.856377	-2.188427	0.168033
16.C	0.418679	0.534574	-1.021871
17.C	-0.465395	1.617951	-0.994460
18.C	-0.469497	2.543243	-2.042552
19.C	0.406758	2.391781	-3.117642
20.C	1.296317	1.314219	-3.140571
21.C	1.310046	0.393011	-2.092452
22.H	1.986405	1.191999	-3.975703
23.H	-1.162351	3.384621	-2.015532
24.H	1.051239	-0.153716	3.207374
25.H	-3.219412	-0.740281	3.047024
26.H	-1.152182	1.741460	-0.156400
27.H	2.016907	-0.438353	-2.119274

PC: CGeN-2 + H_2
E = −3802.57
H = −3664.50
G = −3699.39
N_{imag} = 0

1.Ge	-0.182428	-0.574744	0.986549
2.N	-1.309928	0.906136	1.044031
3.H	-4.685168	3.542290	2.648930
4.H	-2.871365	3.059694	1.046496
5.H	-1.169635	1.612133	0.328404
6.H	3.301170	0.354483	-3.618917
7.H	-5.209145	1.939832	4.492534
8.H	-3.872301	-0.158628	4.692599
9.C	-2.326935	1.168606	1.949485
10.C	-3.090569	2.351373	1.847802
11.C	-4.112396	2.620159	2.752916
12.C	-4.408590	1.725806	3.786123
13.C	-3.658685	0.553248	3.894732
14.C	-2.632573	0.272945	2.994147
15.H	-2.062548	-0.651209	3.102512
16.C	0.985097	-0.275888	-0.546479
17.C	0.623693	-0.741722	-1.819757
18.C	1.452307	-0.514770	-2.920823
19.C	2.653261	0.179035	-2.760113
20.C	3.025210	0.644368	-1.497315
21.C	2.196104	0.416864	-0.396725
22.H	3.963935	1.183309	-1.367963
23.H	1.160786	-0.882321	-3.904859

24.H	-0.309714	-1.289853	-1.957882
25.H	2.501077	0.780867	0.585759
26.H	0.618005	-0.648510	2.301461
27.H	-1.033033	-1.847354	0.808450

TS: CGeP-2 + H_2

E = -2872.72
H = -2755.76
G = -2790.50
N_{imag} = 1,-1134.914i cm^{-1}

1.Ge	-1.105165	0.634377	-0.826098
2.P	-1.273386	2.464971	0.683303
3.H	3.921462	1.542166	-1.244956
4.H	2.545744	-2.074181	-3.139763
5.H	-1.980122	1.089000	2.590966
6.H	4.396498	-0.501523	-2.586131
7.H	-3.306655	1.171394	1.394456
8.C	-2.504351	1.741338	1.882934
9.C	-2.437513	3.531635	-0.318092
10.H	-0.856151	-0.248833	0.646442
11.H	-1.705612	-0.675964	-0.123917
12.H	-2.959501	2.569720	2.442860
13.H	-1.913580	3.910695	-1.204331
14.H	-2.721583	4.395999	0.298492
15.H	-3.345953	3.006094	-0.638684
16.C	0.771957	0.229046	-1.347372
17.C	1.044900	-0.910255	-2.116777
18.C	2.345376	-1.178594	-2.550747
19.C	3.382971	-0.296698	-2.241299
20.C	3.115419	0.850195	-1.491330
21.C	1.817410	1.111040	-1.044244
22.H	0.240481	-1.599090	-2.381964
23.H	1.624846	2.000775	-0.442678

PC: CGeP-2 + H_2

E = -2918.10
H = -2798.68
G = -2833.50
N_{imag} = 0

1.Ge	-0.257710	0.563511	0.625226
2.P	-2.290778	1.239181	-0.316827
3.C	3.263503	1.138109	-1.681729
4.C	2.278068	1.226233	-0.694927
5.H	4.150457	1.769426	-1.620811
6.H	3.881310	0.172525	-3.512028
7.H	1.851528	-1.263643	-3.639003
8.H	-4.224921	1.946978	1.006996
9.H	-2.748115	2.119094	1.994939
10.H	-1.115917	3.031128	-1.519428
11.H	-1.437440	3.554116	0.159839
12.H	-2.751940	3.529630	-1.049112
13.C	-1.847651	3.011596	-0.702482
14.H	-3.469845	0.507438	1.714899
15.C	-3.261675	1.484093	1.260673
16.C	1.131557	0.420247	-0.752855
17.C	0.990530	-0.473597	-1.825996
18.C	1.974291	-0.562990	-2.812624
19.C	3.113015	0.242883	-2.741969
20.H	2.409749	1.928346	0.130100
21.H	0.105834	-1.108474	-1.898018
22.H	0.238843	1.564243	1.699111

23.H	-0.478302	-0.809400	1.297380

TS: CGeN-3 + H_2
E = −6863.81
H = −6624.72
G = −6671.24
N_{imag} = 1,−1218.665i cm^{-1}

1.Ge	0.658271	0.138766	0.710474
2.N	-0.883609	0.578130	1.741775
3.H	-2.450864	1.123026	6.015086
4.H	0.039016	-1.467898	0.197561
5.H	-1.083147	1.574013	1.646739
6.H	-0.181965	2.602219	-4.574000
7.H	-1.864935	-1.223044	6.639744
8.H	-0.792049	-2.702639	4.941674
9.C	-1.101331	0.117827	3.039649
10.C	-1.704494	0.945522	4.010313
11.C	-1.980951	0.462792	5.285437
12.C	-1.653504	-0.850378	5.638526
13.C	-1.050688	-1.674550	4.686445
14.C	-0.778321	-1.205116	3.402403
15.H	1.129275	-1.324466	0.909958
16.C	0.320326	0.835961	-1.124093
17.C	-0.559385	0.276254	-2.087043
18.C	-0.707645	0.927350	-3.325808
19.C	-0.038509	2.112312	-3.610866
20.C	0.810229	2.670054	-2.661945
21.C	1.009306	2.037716	-1.424521
22.H	1.349248	3.592364	-2.876994
23.H	-1.388436	0.499013	-4.060675
24.C	1.958249	2.687967	-0.474000
25.C	3.154906	2.060090	-0.089280
26.C	4.051487	2.699923	0.766783
27.C	3.766311	3.975391	1.257219
28.C	2.584196	4.613162	0.877360
29.C	1.691373	3.977550	0.013437
30.H	-1.935686	-3.948883	-3.466678
31.H	4.977535	2.199792	1.049416
32.H	4.464908	4.472154	1.930020
33.H	2.354577	5.609546	1.254861
34.H	-3.573363	-4.169929	-1.601276
35.C	-1.362092	-0.964481	-1.901194
36.C	-2.282798	-1.104667	-0.848863
37.C	-3.075470	-2.247668	-0.748296
38.C	-2.956178	-3.275586	-1.685856
39.C	-2.041719	-3.149826	-2.732796
40.C	-1.259668	-1.999829	-2.844810
41.H	-3.788312	-2.335017	0.071467
42.H	-1.957323	1.975454	3.750219
43.H	-0.335324	-1.873589	2.664194
44.H	3.391214	1.072406	-0.484037
45.H	0.768857	4.478057	-0.282354
46.H	-2.374702	-0.313771	-0.104791
47.H	-0.543625	-1.907938	-3.661921

PC: CGeN-3 + H_2

E = −6919.00
H = −6677.30
G = −6724.00
N_{imag} = 0

1.Ge	-0.289377	0.005415	0.980068
2.N	-1.552564	1.378230	0.841847
3.H	-5.421297	3.582369	1.891010
4.C	-0.560944	-2.876178	-2.103354
5.H	-1.553837	1.918383	-0.017056
6.H	3.541240	-0.191558	-3.455441
7.H	-5.713318	2.289963	4.009308
8.H	-4.022485	0.556509	4.615325
9.C	-2.641629	1.602941	1.670741
10.C	-3.605691	2.580112	1.340109
11.C	-4.693526	2.820889	2.173527
12.C	-4.860400	2.099677	3.359634
13.C	-3.912481	1.130740	3.694880
14.C	-2.818241	0.881157	2.868873
15.H	-4.005315	-1.098408	-2.089478
16.C	0.901579	0.099873	-0.586241
17.C	0.641448	-0.708519	-1.721313
18.C	1.603839	-0.806708	-2.739446
19.C	2.807812	-0.115419	-2.652601
20.C	3.065020	0.687671	-1.546556
21.C	2.127056	0.808451	-0.507715
22.H	3.996983	1.249015	-1.482339
23.H	1.386442	-1.422013	-3.612362
24.C	2.491306	1.679919	0.644048
25.C	3.701687	1.470381	1.326977
26.C	4.074036	2.291674	2.390277
27.C	3.247855	3.345834	2.785739
28.C	2.048370	3.571504	2.109422
29.C	1.673989	2.746775	1.048243
30.H	-1.663213	-4.695408	-2.441681
31.H	5.010743	2.104396	2.915345
32.H	3.538324	3.988524	3.616488
33.H	1.399459	4.395322	2.405746
34.H	-3.882367	-3.562663	-2.432020
35.C	-0.616790	-1.488073	-1.892597
36.C	-1.873313	-0.863425	-1.895915
37.C	-3.039904	-1.603818	-2.088926
38.C	-2.970993	-2.983721	-2.284541
39.C	-1.726686	-3.617511	-2.292407
40.H	0.407619	-3.376632	-2.098459
41.H	-3.489557	3.152236	0.417225
42.H	-2.090875	0.121153	3.157440
43.H	4.343649	0.640723	1.030048
44.H	0.740752	2.939808	0.521253
45.H	-1.936584	0.215550	-1.762384
46.H	0.499891	0.211571	2.282188
47.H	-1.030604	-1.341047	1.032977

TS: CGeP-3 + H_2

E = −5990.25
H = −5769.00
G = −5817.25
N_{imag} = 1,−1128.554i cm^{-1}

1.Ge	-0.613306	0.503279	0.062691
2.P	-2.193068	1.909736	-1.040083
3.C	-0.245219	-0.792994	-2.796687

4.C	-0.827755	-0.332845	-3.989122
5.C	-1.964169	-0.949566	-4.512714
6.H	4.227636	0.301764	-3.318636
7.C	-2.537470	-2.041785	-3.857393
8.C	-2.703265	2.932035	0.435655
9.C	-3.648874	0.740388	-1.078276
10.H	-0.070807	2.031947	0.661037
11.H	-0.310342	1.152155	1.489182
12.C	-1.968603	-2.510659	-2.671752
13.H	-3.421642	-2.526804	-4.270574
14.C	-0.833880	-1.891236	-2.145621
15.H	-2.405657	-0.573544	-5.435670
16.C	1.107156	0.337699	-0.962930
17.C	1.011515	-0.188824	-2.270565
18.C	2.137272	-0.185423	-3.110549
19.C	3.358234	0.307391	-2.661460
20.C	3.469707	0.778290	-1.355745
21.C	2.361335	0.790994	-0.494596
22.H	4.430536	1.132919	-0.981730
23.H	2.047873	-0.599084	-4.115321
24.C	2.561576	1.287617	0.894024
25.C	2.306682	0.457049	1.997467
26.C	2.528680	0.910929	3.298115
27.C	3.007950	2.203942	3.519073
28.C	3.270534	3.037455	2.430324
29.C	3.052990	2.581553	1.129304
30.H	-2.401695	-3.369526	-2.159095
31.H	2.332857	0.249264	4.142002
32.H	3.180607	2.558243	4.535166
33.H	3.644788	4.048206	2.593582
34.H	-0.388293	0.524621	-4.498436
35.H	-1.973772	3.734709	0.594953
36.H	-2.796706	2.344407	1.360335
37.H	-3.680012	3.384502	0.215104
38.H	-3.446014	-0.069353	-1.789052
39.H	-4.522075	1.300781	-1.441699
40.H	-3.884960	0.307588	-0.097534
41.H	-0.374141	-2.281301	-1.237229
42.H	1.948776	-0.558541	1.828513
43.H	3.250735	3.237336	0.281120

PC: CGeP-3 + H_2
E = −6033.40
H = −5809.79
G = −5858.45
N_{imag} = 0

1.Ge	-0.519860	0.349957	0.468813
2.P	0.191126	2.033781	1.944643
3.C	-2.466334	0.662193	-3.317847
4.C	-2.088545	-1.335150	-2.019019
5.H	-4.479005	0.964601	-4.025062
6.H	2.474177	0.834923	-4.568966
7.C	-3.809091	0.313137	-3.463634
8.C	-4.295445	-0.863487	-2.890444
9.H	4.934038	0.333517	3.263949
10.H	5.097838	2.271818	1.708830
11.H	-5.343698	-1.137818	-3.007024
12.C	-1.587509	-0.151892	-2.585616
13.C	-0.256077	3.500245	0.874144
14.C	-3.428921	-1.688555	-2.170711
15.C	-1.332381	1.986224	3.029593

16.C	0.529132	0.263578	-1.220664
17.C	-0.144676	0.194452	-2.465482
18.C	0.566560	0.414384	-3.657276
19.C	1.932705	0.674547	-3.636598
20.C	2.611676	0.686265	-2.422388
21.C	1.929858	0.475923	-1.212435
22.H	3.691394	0.834414	-2.400504
23.H	0.035955	0.348674	-4.607248
24.C	2.735746	0.452711	0.037999
25.C	2.660238	-0.637582	0.919616
26.C	3.445205	-0.680098	2.071390
27.C	4.323266	0.365200	2.362001
28.C	4.416995	1.449734	1.488079
29.C	3.634531	1.490599	0.335119
30.H	-3.796793	-2.614772	-1.729196
31.H	3.376166	-1.537811	2.740501
32.H	-2.093609	1.586956	-3.759001
33.H	-1.411837	-1.991289	-1.471032
34.H	0.461524	3.580614	0.048458
35.H	-0.172571	4.411280	1.482207
36.H	-1.272333	3.442795	0.460790
37.H	-1.312017	1.079678	3.647351
38.H	-2.274538	2.014186	2.464669
39.H	-1.300691	2.851585	3.705731
40.H	1.994835	-1.468063	0.685209
41.H	3.702712	2.345739	-0.337787
42.H	-0.527490	-1.000857	1.220373
43.H	-1.984055	0.674529	0.094649

TS: CGeN-4 + H_2
E = −13597.23
H = −13034.44
G = −13115.29
N_{imag} = 1,−1195.839i cm^{-1}

1.Ge	0.455484	0.186822	0.746863
2.N	-1.143508	0.030659	1.809121
3.H	-1.287680	1.221577	6.276522
4.H	5.377190	0.556537	0.768944
5.H	-1.620586	0.922527	1.680109
6.H	0.319470	2.289306	-4.785710
7.H	-1.002459	-1.129440	7.034319
8.H	-0.862761	-2.931691	5.337361
9.C	-1.085909	-0.274977	3.201603
10.C	-1.211909	0.757141	4.182888
11.C	-1.189516	0.421644	5.540504
12.C	-1.029783	-0.890594	5.971452
13.C	-0.939449	-1.891222	5.015906
14.C	-0.989786	-1.629650	3.637488
15.H	4.712664	-0.890379	0.004939
16.C	0.213944	0.908141	-1.124577
17.C	-0.595871	0.323534	-2.133133
18.C	-0.555887	0.854937	-3.434462
19.C	0.285399	1.911279	-3.763757
20.C	1.081554	2.479885	-2.778071
21.C	1.041926	2.014949	-1.453078
22.H	1.744627	3.309263	-3.021740
23.H	-1.186827	0.403103	-4.199363
24.C	1.885417	2.761820	-0.458578
25.C	3.125229	2.230501	-0.015517
26.C	3.892692	2.973988	0.887983
27.C	3.472557	4.223851	1.333722

28.C	2.282165	4.761228	0.858342
29.C	1.475702	4.056576	-0.045100
30.H	-1.612611	-4.205372	-2.650851
31.H	4.841138	2.573499	1.243504
32.H	4.083404	4.785696	2.040764
33.H	1.973673	5.752617	1.190945
34.H	-3.969115	-3.876323	-1.975855
35.C	-1.505108	-0.860825	-1.965386
36.C	-2.854420	-0.669865	-1.582107
37.C	-3.720106	-1.770912	-1.589545
38.C	-3.278442	-3.032734	-1.972458
39.C	-1.952092	-3.213718	-2.351205
40.C	-1.048719	-2.144864	-2.359782
41.H	-4.760982	-1.637460	-1.296168
42.C	0.382106	-2.388694	-2.830707
43.H	0.968812	-1.493012	-2.584169
44.C	1.044304	-3.581186	-2.119496
45.H	0.994369	-3.470098	-1.028932
46.H	2.100753	-3.658074	-2.411486
47.H	0.561794	-4.532045	-2.384154
48.C	0.439979	-2.575728	-4.359078
49.H	0.035395	-1.703016	-4.886966
50.H	1.477162	-2.724354	-4.691052
51.H	-0.143429	-3.454730	-4.667266
52.C	-3.403143	0.708727	-1.230688
53.H	-2.544934	1.353589	-0.992576
54.C	-4.124693	1.335573	-2.440418
55.H	-3.454688	1.427495	-3.304476
56.H	-4.500099	2.338105	-2.190862
57.H	-4.981734	0.716915	-2.741897
58.C	-4.328399	0.692592	-0.003505
59.H	-3.844599	0.204743	0.851771
60.H	-4.595301	1.719232	0.282511
61.H	-5.268294	0.162216	-0.209054
62.C	0.224055	4.740236	-0.594469
63.H	-0.345226	3.994885	-1.164683
64.C	-0.707246	5.285192	0.500332
65.H	-1.025223	4.494411	1.188417
66.H	-1.605833	5.723840	0.044937
67.H	-0.223118	6.073152	1.093607
68.C	0.609023	5.873066	-1.567265
69.H	1.235640	5.507116	-2.390141
70.H	-0.291117	6.330827	-2.000767
71.H	1.171580	6.660532	-1.046355
72.C	3.687382	0.930921	-0.583489
73.H	2.841156	0.332324	-0.949717
74.C	4.586556	1.232937	-1.800274
75.H	4.037447	1.777583	-2.578757
76.H	4.967687	0.300262	-2.239254
77.H	5.447851	1.846620	-1.501070
78.C	4.442165	0.078190	0.446711
79.H	3.827816	-0.109626	1.336282
80.C	-0.991121	-2.890417	2.766026
81.H	-1.430033	-3.655758	3.426798
82.C	-1.887618	-2.866446	1.522793
83.H	-2.894202	-2.507907	1.772427
84.H	-1.981246	-3.887076	1.125773
85.H	-1.494315	-2.228070	0.725167
86.C	0.432509	-3.399425	2.462010
87.H	1.000543	-3.548659	3.389783
88.H	0.389839	-4.358821	1.926858

89.H	1.000117	-2.694137	1.842459
90.C	-1.327479	2.253705	3.884910
91.H	-1.512374	2.706581	4.871061
92.C	-0.002235	2.857458	3.390516
93.H	0.813281	2.613517	4.082586
94.H	-0.075671	3.951296	3.322789
95.H	0.288527	2.483933	2.399212
96.C	-2.534181	2.685298	3.027803
97.H	-3.449378	2.165567	3.338388
98.H	-2.702360	3.765131	3.143518
99.H	-2.399729	2.514092	1.949711
100.H	1.248040	-1.153445	0.736624
101.H	0.249710	-1.391910	-0.057071

PC: CGeN-4 + H_2
E = −13649.06
H = −13083.38
G = −13163.98
N_{imag} = 0

1.Ge	-0.271078	0.207489	1.004559
2.N	-1.970448	1.033766	0.827866
3.H	-3.846984	3.956411	3.871811
4.H	3.806938	0.200447	4.118764
5.H	-1.835040	1.892179	0.297366
6.H	3.481781	-0.134218	-3.529845
7.H	-4.955179	2.127232	5.144050
8.H	-4.772654	-0.196719	4.300830
9.C	-2.779964	1.310095	1.972741
10.C	-2.948057	2.658969	2.419256
11.C	-3.734264	2.921026	3.545608
12.C	-4.358859	1.904970	4.259576
13.C	-4.238224	0.606749	3.790092
14.C	-3.494564	0.275106	2.645315
15.H	3.133718	-1.337845	3.570671
16.C	0.899370	0.281884	-0.606781
17.C	0.630425	-0.591629	-1.693783
18.C	1.557479	-0.709103	-2.742064
19.C	2.760578	-0.014166	-2.721224
20.C	3.033079	0.836206	-1.657174
21.C	2.116025	1.018005	-0.608639
22.H	3.971976	1.388232	-1.628149
23.H	1.324113	-1.375584	-3.572158
24.C	2.516646	2.026775	0.433418
25.C	3.059394	1.607644	1.675214
26.C	3.466382	2.579168	2.596242
27.C	3.369354	3.936659	2.302835
28.C	2.882344	4.341008	1.065755
29.C	2.458413	3.407089	0.110588
30.H	-1.593844	-4.694351	-1.426862
31.H	3.876390	2.270738	3.557148
32.H	3.689286	4.678381	3.035106
33.H	2.836260	5.404868	0.831819
34.H	-3.566771	-3.835657	-2.644639
35.C	-0.594794	-1.454348	-1.836340
36.C	-1.726835	-0.958708	-2.527596
37.C	-2.784095	-1.834359	-2.805109
38.C	-2.735659	-3.168262	-2.415356
39.C	-1.623874	-3.647711	-1.729998
40.C	-0.540405	-2.812531	-1.433023
41.H	-3.658605	-1.466541	-3.341534
42.C	0.682573	-3.400261	-0.734751

43.H	1.312702	-2.561342	-0.407690
44.C	0.321168	-4.220256	0.515240
45.H	-0.289581	-3.637407	1.215783
46.H	1.234828	-4.536108	1.037219
47.H	-0.238155	-5.129955	0.257330
48.C	1.518820	-4.251675	-1.709948
49.H	1.848802	-3.663763	-2.575530
50.H	2.411554	-4.649726	-1.207433
51.H	0.932588	-5.102531	-2.084637
52.C	-1.797250	0.477497	-3.035410
53.H	-1.002202	1.044759	-2.531457
54.C	-1.515298	0.538213	-4.549361
55.H	-0.530466	0.118805	-4.791569
56.H	-1.541221	1.577540	-4.906058
57.H	-2.270837	-0.031223	-5.108864
58.C	-3.134122	1.160496	-2.704174
59.H	-3.351445	1.099962	-1.630610
60.H	-3.100426	2.219249	-2.996362
61.H	-3.971464	0.701967	-3.247970
62.C	2.022654	3.913718	-1.264418
63.H	1.596264	3.066128	-1.816517
64.C	0.944977	5.008218	-1.205345
65.H	0.048793	4.664245	-0.678036
66.H	0.651359	5.300329	-2.223041
67.H	1.308330	5.912466	-0.697799
68.C	3.239868	4.422582	-2.062962
69.H	4.010049	3.648594	-2.168715
70.H	2.934257	4.740079	-3.069720
71.H	3.701739	5.284921	-1.561953
72.C	3.304463	0.132904	1.982146
73.H	2.609943	-0.457328	1.367168
74.C	4.732309	-0.265531	1.554713
75.H	4.899572	-0.072525	0.487681
76.H	4.907298	-1.334488	1.740726
77.H	5.478274	0.306908	2.123728
78.C	3.060297	-0.248740	3.449734
79.H	2.065665	0.066409	3.789798
80.C	-3.630981	-1.199956	2.242238
81.H	-4.644157	-1.458053	2.591250
82.C	-3.637320	-1.515511	0.743466
83.H	-4.311923	-0.840121	0.202644
84.H	-3.996513	-2.543330	0.594705
85.H	-2.651609	-1.437931	0.278178
86.C	-2.688208	-2.124790	3.038556
87.H	-2.813573	-1.974536	4.119069
88.H	-2.909153	-3.177838	2.812161
89.H	-1.634080	-1.943468	2.797569
90.C	-2.310725	3.889714	1.771236
91.H	-2.736701	4.734971	2.333068
92.C	-0.791422	3.971727	1.993440
93.H	-0.551738	3.850487	3.057068
94.H	-0.407036	4.947895	1.668743
95.H	-0.236526	3.202124	1.443142
96.C	-2.718969	4.154144	0.307742
97.H	-3.804315	4.054791	0.179335
98.H	-2.434833	5.176606	0.022131
99.H	-2.239384	3.486010	-0.422324
100.H	0.436596	0.833431	2.218833
101.H	-0.518137	-1.280579	1.266218

TS: CGeP-4 + H_2

E = −12587.91

H = −12053.28

G = −12139.42

N_{imag} = 1,−1167.090i cm^{-1}

1.Ge	-0.996835	0.653467	-0.650960
2.P	-1.457827	2.643773	0.568947
3.C	2.392006	-0.552534	1.916792
4.H	4.118600	4.737461	-0.662960
5.H	3.528656	4.601794	-2.335660
6.H	4.386880	-0.618761	-2.794512
7.H	2.029002	-0.968748	0.967320
8.C	3.855784	-1.004718	2.090265
9.H	4.484413	-0.650276	1.263789
10.H	3.921482	-2.101417	2.123638
11.H	4.276894	-0.609933	3.025691
12.C	1.517281	-1.140316	3.034755
13.H	0.462868	-0.866064	2.900586
14.H	1.584501	-2.236980	3.029791
15.H	1.833519	-0.798319	4.029647
16.C	0.904149	0.261164	-1.237432
17.C	0.995301	-0.619478	-2.348117
18.C	2.254643	-0.931791	-2.885392
19.C	3.415882	-0.374175	-2.363426
20.C	3.328746	0.479093	-1.269245
21.C	2.091913	0.798772	-0.682831
22.H	4.235908	0.891822	-0.828067
23.H	2.309846	-1.631369	-3.719247
24.C	2.164881	1.629158	0.568365
25.C	2.314495	0.969279	1.813749
26.C	2.485631	1.741436	2.969415
27.C	2.529716	3.130549	2.906040
28.C	2.408643	3.770043	1.676670
29.C	2.227967	3.041306	0.494844
30.H	-2.036222	-4.170854	-2.653997
31.H	2.602589	1.247144	3.934080
32.H	2.667805	3.715623	3.815805
33.H	2.453436	4.857890	1.635778
34.H	-3.042150	-3.297746	-4.736745
35.C	-0.183232	-1.318321	-2.976742
36.C	-0.748607	-0.824965	-4.179491
37.C	-1.777766	-1.552753	-4.790438
38.C	-2.240485	-2.745717	-4.245438
39.C	-1.673443	-3.232107	-3.072233
40.C	-0.644755	-2.539214	-2.423356
41.H	-2.225629	-1.179907	-5.711015
42.C	-0.014848	-3.146794	-1.173688
43.H	0.552076	-2.353036	-0.667873
44.C	-1.052763	-3.680514	-0.172747
45.H	-1.804409	-2.917889	0.071857
46.H	-0.558097	-3.986818	0.758776
47.H	-1.584654	-4.559020	-0.562926
48.C	0.987671	-4.254134	-1.553654
49.H	1.779069	-3.867779	-2.208246
50.H	1.461563	-4.673662	-0.655072
51.H	0.479764	-5.071887	-2.084295
52.C	-0.249487	0.456188	-4.841442
53.H	0.262646	1.048479	-4.068796
54.C	0.779031	0.149120	-5.948688
55.H	1.651995	-0.384970	-5.556190
56.H	1.131366	1.079907	-6.415145

57.H	0.324447	-0.473373	-6.732386
58.C	-1.384755	1.317531	-5.420104
59.H	-2.190619	1.473718	-4.693012
60.H	-0.997186	2.300379	-5.719653
61.H	-1.825667	0.857422	-6.315168
62.C	2.164893	3.776359	-0.840683
63.H	1.683483	3.101259	-1.563359
64.C	1.334029	5.068114	-0.785947
65.H	0.343258	4.881727	-0.353281
66.H	1.206783	5.476043	-1.798246
67.H	1.828217	5.845135	-0.186316
68.C	3.581322	4.085725	-1.366464
69.H	4.173436	3.172401	-1.499966
70.Si	-2.651027	1.897776	2.372634
71.C	-3.860149	3.256055	2.895094
72.H	-4.288014	2.992349	3.873996
73.H	-3.360596	4.227844	3.000354
74.H	-4.693096	3.371845	2.190200
75.C	-3.640950	0.305953	2.124056
76.H	-4.345636	0.384154	1.286918
77.H	-4.220357	0.101019	3.036979
78.H	-2.996738	-0.564088	1.944177
79.C	-1.417554	1.661913	3.778333
80.H	-0.954417	2.619572	4.049013
81.H	-1.928233	1.262203	4.666886
82.H	-0.609692	0.971689	3.508542
83.Si	-3.037086	3.475884	-0.860896
84.C	-3.553249	5.183190	-0.230911
85.H	-4.200475	5.669959	-0.975526
86.H	-4.106993	5.136444	0.714290
87.H	-2.676577	5.826511	-0.077334
88.C	-2.156691	3.725134	-2.512708
89.H	-1.318547	4.426165	-2.407445
90.H	-2.852607	4.136230	-3.258549
91.H	-1.760721	2.783441	-2.913805
92.C	-4.571825	2.406432	-1.132145
93.H	-5.166387	2.304421	-0.214796
94.H	-5.217493	2.858646	-1.899673
95.H	-4.296876	1.399135	-1.472310
96.H	-0.660459	-0.084621	0.909064
97.H	-1.457501	-0.615835	0.202407

PC: CGeP-4 + H_2
E = −12634.16
H = −12096.21
G = −12183.91
N_{imag} = 0

1.Ge	-0.864724	0.512950	-0.235929
2.P	-1.444995	2.620200	0.611060
3.C	2.352818	-0.627095	1.832911
4.H	4.171857	4.823788	-0.422432
5.H	3.609622	4.791239	-2.109369
6.H	4.255714	-0.291024	-3.072838
7.H	1.889629	-0.994478	0.907086
8.C	3.818743	-1.106571	1.834732
9.H	4.363825	-0.729940	0.960302
10.H	3.867485	-2.204550	1.821081
11.H	4.339576	-0.752938	2.735792
12.C	1.587577	-1.254506	3.007701
13.H	0.545086	-0.913736	3.036729
14.H	1.584469	-2.348723	2.912404

15.H	2.049913	-1.015710	3.975174
16.C	0.869196	0.297620	-1.198989
17.C	0.918228	-0.530613	-2.352886
18.C	2.143676	-0.722861	-3.011619
19.C	3.312551	-0.127506	-2.551145
20.C	3.273948	0.646096	-1.396389
21.C	2.071954	0.863255	-0.704033
22.H	4.192731	1.074817	-0.997036
23.H	2.171590	-1.375708	-3.883759
24.C	2.177807	1.622823	0.591694
25.C	2.299007	0.898527	1.805835
26.C	2.473819	1.607351	3.001032
27.C	2.554188	2.995719	3.009192
28.C	2.467074	3.698165	1.812350
29.C	2.280175	3.036076	0.593132
30.H	-1.857054	-4.295354	-2.392552
31.H	2.564167	1.062786	3.940478
32.H	2.693521	3.530468	3.949217
33.H	2.539886	4.785131	1.827165
34.H	-3.098143	-3.522603	-4.386615
35.C	-0.247325	-1.322180	-2.890659
36.C	-0.941956	-0.890965	-4.048326
37.C	-1.963392	-1.698207	-4.565186
38.C	-2.298346	-2.909672	-3.970170
39.C	-1.601231	-3.337895	-2.846020
40.C	-0.570955	-2.567275	-2.293256
41.H	-2.507828	-1.372980	-5.451218
42.C	0.214589	-3.131753	-1.112937
43.H	0.794577	-2.310039	-0.672075
44.C	-0.684229	-3.704243	-0.004858
45.H	-1.417601	-2.963936	0.338891
46.H	-0.074224	-4.005758	0.857368
47.H	-1.233356	-4.594244	-0.341348
48.C	1.222348	-4.192881	-1.597389
49.H	1.915981	-3.774620	-2.337889
50.H	1.813104	-4.579644	-0.755174
51.H	0.700705	-5.040063	-2.064767
52.C	-0.593646	0.407816	-4.769182
53.H	-0.020410	1.031923	-4.067790
54.C	0.304826	0.142449	-5.993962
55.H	1.242038	-0.351341	-5.711607
56.H	0.556280	1.085791	-6.498833
57.H	-0.212189	-0.503368	-6.717750
58.C	-1.836178	1.203628	-5.203988
59.H	-2.536315	1.351550	-4.373057
60.H	-1.538424	2.190989	-5.581249
61.H	-2.377695	0.696689	-6.014240
62.C	2.234157	3.852443	-0.695167
63.H	1.769357	3.222790	-1.467601
64.C	1.391857	5.132217	-0.565401
65.H	0.398238	4.911906	-0.155991
66.H	1.273339	5.606225	-1.549623
67.H	1.873547	5.870984	0.089997
68.C	3.654503	4.212824	-1.175697
69.H	4.262919	3.319411	-1.359489
70.Si	-2.882170	1.921755	2.247760
71.C	-3.975486	3.379119	2.754232
72.H	-4.512543	3.124211	3.679956
73.H	-3.384240	4.283659	2.947314
74.H	-4.727503	3.617744	1.991252
75.C	-3.994500	0.458019	1.807090

76.H	-4.658696	0.685161	0.963991
77.H	-4.622455	0.201824	2.673742
78.H	-3.410930	-0.433819	1.544691
79.C	-1.803324	1.448060	3.721121
80.H	-1.182225	2.294106	4.042297
81.H	-2.431744	1.138197	4.569055
82.H	-1.130269	0.615936	3.478485
83.Si	-2.766019	3.472789	-1.041109
84.C	-3.333559	5.186457	-0.478900
85.H	-3.872016	5.684941	-1.298628
86.H	-4.006069	5.144335	0.386454
87.H	-2.475773	5.816235	-0.208226
88.C	-1.647753	3.704098	-2.543882
89.H	-0.832825	4.401658	-2.313336
90.H	-2.221518	4.116712	-3.386270
91.H	-1.198890	2.759852	-2.875303
92.C	-4.279479	2.450615	-1.527854
93.H	-5.016076	2.410465	-0.714651
94.H	-4.772123	2.905004	-2.400456
95.H	-4.009384	1.419754	-1.790904
96.H	-1.963897	-0.025655	-1.170130
97.H	-0.811756	-0.428157	0.990545

TS: CSiP + HCN
E = −2019.60
H = −1932.74
G = −1965.44
N_{imag} = 1,−147.281i cm^{-1}

1.C	-2.786290	0.139043	-1.005718
2.P	-2.194786	0.274291	0.749543
3.H	-3.670839	0.776319	-1.127177
4.H	-0.681627	-1.646133	0.571789
5.N	-3.592813	2.262143	3.864496
6.Si	-2.148527	2.440414	1.278769
7.H	0.236215	2.715374	1.848566
8.C	-4.126749	2.767238	2.954026
9.C	-0.538928	-0.569564	0.730812
10.H	0.121604	-0.177873	-0.052979
11.H	-0.056398	-0.432520	1.705328
12.H	-3.079486	-0.896573	-1.218230
13.H	-2.025985	0.451100	-1.732922
14.C	-0.654219	2.532828	2.473059
15.H	-0.760303	3.382263	3.159546
16.H	-0.468951	1.628567	3.064447
17.H	-4.860158	3.255690	2.335681

PC: CSiP + HCN
E = −2047.13
H = −1958.91
G = −1989.88
N_{imag} = 0

1.C	-3.508883	-0.047690	0.040868
2.P	-1.890268	0.016413	0.963098
3.H	-4.328764	-0.133492	0.763588
4.H	-0.577363	-0.417684	-1.055540
5.N	-3.801491	2.346787	2.650567
6.Si	-2.131756	2.015023	1.982047
7.H	0.213806	2.712163	2.488795
8.C	-3.516793	3.134501	1.676562
9.C	-0.714641	0.468623	-0.421995
10.H	-1.070891	1.300223	-1.042855

11.H	0.264537	0.727762	-0.000531
12.H	-3.511427	-0.948148	-0.586988
13.H	-3.681883	0.831612	-0.592857
14.C	-0.695330	2.525481	3.075179
15.H	-0.951162	3.444577	3.617277
16.H	-0.471511	1.737370	3.806836
17.H	-4.133374	3.936345	1.243020

TS: CGeP + HCN

E = −2004.77

H = −1918.25

G = −1952.06

N_{imag} = 1,-90.891i cm^{-1}

1.C	-2.757602	0.152526	-0.997199
2.P	-2.261593	0.162040	0.803793
3.H	-3.714484	0.678593	-1.105154
4.H	-0.542491	-1.545302	0.432887
5.N	-3.597771	2.146605	3.745348
6.Ge	-2.149703	2.472909	1.268768
7.H	0.336577	2.572356	1.853321
8.C	-4.147191	2.848606	2.991590
9.C	-0.512675	-0.483737	0.712745
10.H	0.112667	0.052192	-0.012894
11.H	-0.050107	-0.411639	1.703908
12.H	-2.906593	-0.885579	-1.322135
13.H	-2.019723	0.624730	-1.658702
14.C	-0.553030	2.525495	2.500426
15.H	-0.581453	3.432151	3.115276
16.H	-0.466599	1.646352	3.147080
17.H	-4.820987	3.500089	2.462819

PC: CGeP + HCN

E = −1862.88

H = −1775.51

G = −1807.43

N_{imag} = 0

1.C	-2.129778	0.081383	-0.959264
2.P	-2.410436	-0.054756	0.886168
3.H	-3.020049	0.512926	-1.433224
4.H	-0.509033	-1.576764	1.075036
5.N	-2.887159	1.759369	3.569476
6.Ge	-2.451239	2.248973	1.414255
7.H	-0.180425	3.318235	0.837613
8.C	-3.656941	2.531631	3.027115
9.C	-0.694073	-0.549647	1.419930
10.H	0.096028	0.099525	1.020836
11.H	-0.648719	-0.542787	2.515254
12.H	-1.995479	-0.933385	-1.357958
13.H	-1.251309	0.679420	-1.235621
14.C	-0.657795	3.054185	1.789101
15.H	-0.804936	3.967041	2.377076
16.H	-0.016554	2.360142	2.341694
17.H	-4.552895	3.120981	3.244029

TS: CSiP + CO_2

E = −2094.67

H = −2010.94

G = −2045.01

N_{imag} = 1,-197.142i cm^{-1}

1.C	-1.904995	0.581587	-1.339777
2.P	-2.505759	0.320522	0.399984

3.H	-2.268541	1.549105	-1.704440
4.H	-2.096680	-2.093484	0.446295
5.O	-3.099510	1.862923	4.787024
6.Si	-1.878617	1.943976	1.793070
7.H	-0.982899	3.556437	0.130027
8.O	-4.160791	2.574024	2.821063
9.C	-1.643202	-1.229416	0.947411
10.H	-0.567594	-1.214982	0.732642
11.H	-1.787446	-1.347252	2.028199
12.H	-2.324529	-0.202033	-1.983589
13.H	-0.810976	0.556718	-1.416328
14.C	-1.927243	3.504420	0.695350
15.H	-2.751600	3.504399	-0.029382
16.H	-2.000739	4.409702	1.310347
17.C	-3.442638	2.176835	3.701694

PC: CSiP + CO_2
E = −2116.74
H = −2031.22
G = −2064.61
N_{imag} = 0

1.C	-3.856853	0.025088	-0.090087
2.P	-2.260244	-0.026253	0.868717
3.H	-4.694179	0.028345	0.617833
4.H	-0.923731	-0.563493	-1.099541
5.H	-0.412242	1.658891	3.416747
6.Si	-2.342870	1.972053	1.901754
7.H	0.058062	2.616231	1.994452
8.O	-3.758588	2.120323	2.936263
9.C	-1.000464	0.330130	-0.467095
10.H	-1.256454	1.188384	-1.100188
11.H	-0.017358	0.495193	-0.009818
12.H	-3.925598	-0.891800	-0.689450
13.H	-3.942167	0.895755	-0.751176
14.C	-0.738360	2.455306	2.733125
15.H	-0.872642	3.380572	3.306712
16.C	-3.820999	3.082296	1.929607
17.O	-4.655816	3.911466	1.710624

TS: CGeP + CO_2
E = −2068.98
H = −1967.01
G = −1999.19
N_{imag} = 1,−134.155i cm^{-1}

1.C	-1.566590	0.640821	-1.236995
2.P	-2.608379	0.281619	0.271986
3.H	-1.874642	1.598653	-1.671908
4.H	-2.004228	-2.091056	0.287115
5.O	-2.994996	2.086911	4.854751
6.Ge	-1.926153	1.936546	1.811166
7.H	-2.818772	3.592403	-0.009216
8.O	-4.106629	2.330547	2.803752
9.C	-1.764186	-1.241693	0.940230
10.H	-0.673205	-1.146475	1.007995
11.H	-2.164136	-1.462223	1.937544
12.H	-1.762028	-0.143045	-1.980931
13.H	-0.488158	0.665542	-1.035320
14.C	-1.974257	3.598102	0.689457
15.H	-2.032521	4.481903	1.333917
16.H	-1.039490	3.646455	0.112648
17.C	-3.328472	2.172782	3.724380

PC: CGeP + CO_2
E = −2079.79
H = −1995.73
G = −2030.68
N_{imag} = 0

1.C	-1.013399	0.476380	-0.849723
2.P	-2.567841	0.313387	0.178092
3.H	-1.049334	1.407507	-1.428194
4.H	-2.177548	-2.056459	0.599233
5.O	-2.648125	1.935055	4.774606
6.Ge	-2.182792	2.067105	1.700486
7.H	-3.197060	3.764465	0.059015
8.O	-3.956766	2.020406	2.845694
9.C	-2.131564	-1.159308	1.231287
10.H	-1.136154	-1.095438	1.687428
11.H	-2.884002	-1.268322	2.021569
12.H	-1.000822	-0.356895	-1.564847
13.H	-0.085364	0.445550	-0.265297
14.C	-2.356291	3.805025	0.760817
15.H	-2.521206	4.597613	1.497900
16.H	-1.432090	4.014926	0.208270
17.C	-2.936174	1.988060	3.616073

TS: CSiP + H_2O
E = −1888.26
H = −1800.49
G = −1831.10
N_{imag} = 1,−1257.804i cm^{-1}

1.C	0.670684	0.336875	0.775343
2.P	-0.915739	1.109692	0.167955
3.H	1.299904	1.120025	1.215980
4.H	-1.418435	-0.901738	-1.134262
5.H	-1.657913	4.068922	2.713983
6.Si	-1.924879	1.649870	2.145007
7.H	-4.270823	1.397641	1.306189
8.H	-1.191699	2.465458	3.410566
9.C	-1.891936	-0.430124	-0.263325
10.H	-1.936693	-1.167553	0.549136
11.H	-2.912136	-0.149362	-0.552097
12.H	1.211044	-0.085046	-0.082458
13.H	0.509679	-0.451278	1.522231
14.C	-3.656209	2.250294	1.630550
15.H	-4.179366	2.720775	2.473966
16.H	-3.605355	2.963819	0.794609
17.O	-1.004598	3.357459	2.553677

PC: CSiP + H_2O
E = −1956.22
H = −1866.29
G = −1896.74
N_{imag} = 0

1.C	-0.248818	-0.620542	1.079491
2.P	-0.494207	1.085891	0.349486
3.H	0.449140	-0.557352	1.923901
4.H	-1.552583	0.193914	-1.673624
5.H	-2.366137	4.182409	2.179509
6.Si	-1.585807	2.036922	2.091413
7.H	-2.932852	0.154301	3.029111
8.H	-0.623612	2.071345	3.243922
9.C	-1.925343	0.757636	-0.808475
10.H	-2.745118	0.191328	-0.347083

11.H	-2.312970	1.716123	-1.174793
12.H	0.212168	-1.260397	0.315983
13.H	-1.178594	-1.096808	1.418250
14.C	-3.152801	1.173138	2.680818
15.H	-3.595382	1.719344	3.526687
16.H	-3.907857	1.111247	1.886225
17.O	-1.925380	3.585936	1.555781

TS: CGeP + H_2O
E = −1862.88
H = −1775.51
G = −1807.43
N_{imag} = 1,-1215.901i cm^{-1}

1.C	0.816383	0.408134	0.812594
2.P	-0.779020	1.039796	0.082972
3.H	1.411762	1.264256	1.152444
4.H	-1.236289	-1.135321	-0.937817
5.H	-1.402479	4.359220	2.437114
6.Ge	-1.833078	1.852901	2.028355
7.H	-4.258474	1.650511	1.169978
8.H	-1.084419	2.655344	3.255995
9.C	-1.710971	-0.570696	-0.124293
10.H	-1.717085	-1.199060	0.775841
11.H	-2.744730	-0.361529	-0.425910
12.H	1.383769	-0.106233	0.025141
13.H	0.668482	-0.281561	1.653586
14.C	-3.620409	2.503543	1.434792
15.H	-4.108244	3.065255	2.239576
16.H	-3.504439	3.142905	0.550498
17.O	-0.756709	3.638305	2.299292

PC: CGeP + H_2O
E = −1909.91
H = −1820.61
G = −1852.22
N_{imag} = 0

1.C	-0.114840	-0.660970	1.168727
2.P	-0.377901	0.983188	0.317135
3.H	0.567335	-0.526052	2.017357
4.H	-1.370840	-0.059517	-1.663027
5.H	-1.148080	4.212904	1.117193
6.Ge	-1.585272	2.081460	2.001134
7.H	-3.296637	0.393734	2.894536
8.H	-0.678692	2.188260	3.259884
9.C	-1.775727	0.544443	-0.840136
10.H	-2.585543	-0.017171	-0.356649
11.H	-2.186134	1.466129	-1.270611
12.H	0.369199	-1.344539	0.458582
13.H	-1.042632	-1.128125	1.524371
14.C	-3.366408	1.402903	2.473120
15.H	-3.806244	2.073587	3.220167
16.H	-4.010021	1.384435	1.587337
17.O	-1.958470	3.760712	1.406271

TS: CSiP + NH_3
E = −2004.08
H = −1908.58
G = −1938.12
N_{imag} = 1,-1404.439i cm^{-1}

1.C	0.631498	0.364026	0.662213
2.P	-0.985483	1.148874	0.150730

3.H	1.313730	1.142844	1.026581
4.H	-1.577644	-0.899544	-1.059893
5.H	-0.098213	3.589937	2.620240
6.Si	-1.849436	1.761407	2.189173
7.H	-4.270512	1.440031	1.600305
8.H	-1.354032	2.552813	3.563577
9.C	-1.993888	-0.392734	-0.179424
10.H	-1.997671	-1.099720	0.661115
11.H	-3.026984	-0.109361	-0.414634
12.H	1.097442	-0.097763	-0.218536
13.H	0.511824	-0.395338	1.446149
14.C	-3.631724	2.309360	1.806672
15.H	-4.064448	2.830273	2.671959
16.H	-3.674295	2.978433	0.933785
17.N	-1.111878	3.484397	2.602668
18.H	-1.581510	4.378917	2.421111

PC: CSiP + NH_3
E = −2066.20
H = −1968.65
G = −1999.45
N_{imag} = 0

1.C	-0.251733	-0.583980	1.037143
2.P	-0.551481	1.121876	0.329272
3.H	0.474091	-0.510739	1.856939
4.H	-1.673698	0.205423	-1.654012
5.H	-1.252492	4.424000	1.529233
6.Si	-1.577053	2.114915	2.108303
7.H	-2.960784	0.199287	2.926107
8.H	-0.536496	2.095710	3.185988
9.C	-2.016913	0.775021	-0.780547
10.H	-2.817946	0.209393	-0.285814
11.H	-2.424510	1.726002	-1.146916
12.H	0.189436	-1.219364	0.258153
13.H	-1.162644	-1.070922	1.410868
14.C	-3.145587	1.258061	2.702551
15.H	-3.511947	1.740458	3.618770
16.H	-3.943793	1.307642	1.949390
17.N	-1.986859	3.781777	1.816736
18.H	-2.841537	3.990319	1.306987

TS: CGeP + NH_3
E = −1975.61
H = −1880.85
G = −1912.92
N_{imag} = 1,−1318.314i cm^{-1}

1.C	0.784666	0.382784	0.747398
2.P	-0.799746	1.112258	0.079844
3.H	1.444933	1.193452	1.080518
4.H	-1.338727	-1.027124	-0.985938
5.H	0.012063	3.838431	2.441169
6.Ge	-1.796344	1.889844	2.091620
7.H	-4.278257	1.583591	1.431319
8.H	-1.288533	2.654495	3.475646
9.C	-1.786481	-0.459321	-0.159229
10.H	-1.810496	-1.102343	0.730660
11.H	-2.814013	-0.205643	-0.447214
12.H	1.297453	-0.150142	-0.064921
13.H	0.624469	-0.310946	1.583126
14.C	-3.638960	2.460647	1.588462
15.H	-4.068602	3.061653	2.398603

16.H	-3.612995	3.053220	0.665196
17.N	-1.000532	3.732448	2.479941
18.H	-1.479934	4.614627	2.284212

PC: CGeP + NH_3
E = −2022.31
H = −1925.41
G = −1957.43
N_{imag} = 0

1.C	-0.220921	-0.654563	1.102335
2.P	-0.416071	1.032701	0.318650
3.H	0.473580	-0.582621	1.948725
4.H	-1.459198	0.059045	-1.674574
5.H	-1.185328	4.441570	1.364217
6.Ge	-1.585715	2.123433	2.051739
7.H	-3.135958	0.255273	2.907030
8.H	-0.624391	2.204431	3.261661
9.C	-1.832539	0.680135	-0.849363
10.H	-2.675671	0.161085	-0.374413
11.H	-2.189370	1.623440	-1.282070
12.H	0.224407	-1.334207	0.363355
13.H	-1.166664	-1.090180	1.451278
14.C	-3.293998	1.288247	2.577422
15.H	-3.724672	1.865464	3.403621
16.H	-3.999442	1.288414	1.738511
17.N	-2.007621	3.904527	1.645362
18.H	-2.683406	3.976385	0.882573

TS: CSiP + PH_3
E = −1912.59
H = −1821.71
G = −1853.21
N_{imag} = 1,-907.901i cm^{-1}

1.C	0.775468	0.481872	0.634920
2.P	-0.869961	1.115496	0.024214
3.H	1.401069	1.333545	0.929992
4.H	-1.379798	-1.075296	-0.943424
5.H	0.247500	3.721579	3.154480
6.Si	-1.770879	1.966339	1.957416
7.H	-4.167881	1.394537	1.572133
8.H	-1.746781	3.154502	3.610916
9.C	-1.812726	-0.493211	-0.119162
10.H	-1.779668	-1.101868	0.794145
11.H	-2.858439	-0.281719	-0.373142
12.H	1.283237	-0.035485	-0.189872
13.H	0.684529	-0.203879	1.487144
14.C	-3.601661	2.335773	1.542149
15.H	-4.040649	3.009467	2.290565
16.H	-3.734955	2.782571	0.548660
17.P	-0.999481	3.998628	2.551084
18.H	-0.656486	5.338383	2.009733

PC: CSiP + PH_3
E = −1971.21
H = −1878.28
G = −1910.10
N_{imag} = 0

1.C	-0.321997	-0.515908	1.016782
2.P	-0.558483	1.231607	0.390846
3.H	0.395344	-0.508421	1.846858
4.H	-1.634736	0.423817	-1.654468

5.H	-1.055765	4.630701	1.067442
6.Si	-1.625163	2.152050	2.177379
7.H	-2.866249	0.183502	3.105229
8.H	-0.594839	2.220510	3.264080
9.C	-1.997386	0.968738	-0.773051
10.H	-2.826534	0.402909	-0.328038
11.H	-2.369757	1.942386	-1.114907
12.H	0.110618	-1.119055	0.207433
13.H	-1.252150	-0.994462	1.350616
14.C	-3.132726	1.212436	2.829620
15.H	-3.527717	1.710404	3.725496
16.H	-3.936865	1.170448	2.083553
17.P	-2.247740	4.313492	1.792139
18.H	-2.975300	4.001967	0.598671

TS: CGeP + PH_3
E = −1889.96
H = −1799.72
G = −1832.58
N_{imag} = 1,−881.858i cm^{-1}

1.C	0.776829	0.509736	0.533156
2.P	-0.914313	1.036453	-0.049549
3.H	1.377761	1.402608	0.745841
4.H	-1.375545	-1.232737	-0.842557
5.H	0.337592	3.737306	3.036744
6.Ge	-1.790083	1.983603	1.960561
7.H	-4.286159	1.482571	1.596157
8.H	-1.621597	3.038775	3.613073
9.C	-1.789692	-0.615407	-0.033708
10.H	-1.678291	-1.161354	0.912333
11.H	-2.856416	-0.466381	-0.240549
12.H	1.272280	-0.041922	-0.277343
13.H	0.749313	-0.123846	1.429114
14.C	-3.706470	2.412690	1.551418
15.H	-4.100516	3.109223	2.300644
16.H	-3.812016	2.852046	0.553503
17.P	-0.958501	4.094532	2.596721
18.H	-0.622163	5.444643	2.106102

PC: CGeP + PH_3
E = −1939.37
H = −1847.00
G = −1880.12
N_{imag} = 0

1.C	-0.291801	-0.604725	1.102389
2.P	-0.418854	1.112246	0.372774
3.H	0.378760	-0.581728	1.970308
4.H	-1.405403	0.226991	-1.683167
5.H	-0.952696	4.651736	0.898364
6.Ge	-1.624322	2.172304	2.088921
7.H	-3.036593	0.225222	3.013838
8.H	-0.670346	2.279345	3.305701
9.C	-1.798976	0.829811	-0.853741
10.H	-2.668396	0.312559	-0.426962
11.H	-2.120752	1.793802	-1.266786
12.H	0.158250	-1.266973	0.350152
13.H	-1.258657	-1.027478	1.405682
14.C	-3.274609	1.239037	2.672288
15.H	-3.725794	1.794933	3.502360
16.H	-3.996667	1.180872	1.850410
17.P	-2.204333	4.386317	1.538840

18.H	-2.828406	4.018720	0.303489

TS: CSiP + CH_4
E = −2092.64
H = −1990.16
G = −2021.19
N_{imag} = 1,-1123.167i cm^{-1}

1.C	0.556348	0.404148	0.556927
2.P	-1.157490	1.087821	0.247309
3.H	1.246131	1.229468	0.774491
4.H	-1.792369	-1.088279	-0.673938
5.H	-0.106576	3.679231	2.791213
6.Si	-1.672741	1.783587	2.371937
7.H	-4.544827	1.627998	1.511642
8.H	-3.140028	1.528458	2.750739
9.C	-2.120307	-0.512755	0.201955
10.H	-1.981211	-1.124774	1.103113
11.H	-3.187983	-0.295430	0.077375
12.H	0.905111	-0.085403	-0.362260
13.H	0.592238	-0.318378	1.382657
14.C	-3.775396	2.391637	1.661693
15.H	-4.135309	3.204064	2.298213
16.H	-3.415241	2.765171	0.696211
17.C	-1.178527	3.613652	2.559652
18.H	-1.371113	4.205518	1.654346
19.H	-1.719924	4.064866	3.402016

PC: CSiP + CH_4
E = −2156.67
H = −2052.56
G = −2083.33
N_{imag} = 0

1.C	-0.405644	-0.630679	1.120849
2.P	-0.642495	1.079323	0.402783
3.H	0.340869	-0.583341	1.923523
4.H	-1.809960	0.164397	-1.550580
5.H	-1.124433	4.416218	1.328186
6.Si	-1.622959	2.118255	2.174741
7.H	-2.950278	0.228503	3.164957
8.H	-0.571013	2.123761	3.244157
9.C	-2.127303	0.773009	-0.693581
10.H	-2.951774	0.256611	-0.183978
11.H	-2.493377	1.729849	-1.086651
12.H	-0.011248	-1.289974	0.336472
13.H	-1.329032	-1.072940	1.518196
14.C	-3.163203	1.261399	2.860022
15.H	-3.532102	1.800112	3.744441
16.H	-3.974984	1.238322	2.120580
17.C	-2.020024	3.900786	1.698375
18.H	-2.785674	3.944586	0.911798
19.H	-2.399631	4.458147	2.565851

TS: CGeP + CH_4
E = −2068.98
H = −1967.01
G = −1999.19
N_{imag} = 1,-1071.411i cm^{-1}

1.C	0.559288	0.368263	0.517062
2.P	-1.148414	1.062868	0.207247
3.H	1.255902	1.188829	0.730220
4.H	-1.802524	-1.106851	-0.710783

5.H	-0.130379	3.788646	2.877558
6.Ge	-1.667612	1.783361	2.402470
7.H	-4.539596	1.609561	1.456119
8.H	-3.144745	1.486326	2.844743
9.C	-2.116965	-0.532306	0.171191
10.H	-1.966815	-1.149037	1.067251
11.H	-3.185189	-0.309946	0.062998
12.H	0.901451	-0.127641	-0.401630
13.H	0.592575	-0.353332	1.343624
14.C	-3.814097	2.409931	1.624421
15.H	-4.208434	3.186174	2.284513
16.H	-3.456139	2.823147	0.675419
17.C	-1.186420	3.711719	2.593905
18.H	-1.348359	4.255103	1.655782
19.H	-1.794001	4.159548	3.388618

PC: CGeP + CH_4
E = −2120.04
H = −2016.48
G = −2048.61
N_{imag} = 0

1.C	-0.382359	-0.680065	1.059648
2.P	-0.602694	1.024311	0.325249
3.H	0.389148	-0.640639	1.838676
4.H	-1.836447	0.094320	-1.576661
5.H	-1.114514	4.482389	1.297348
6.Ge	-1.582615	2.108673	2.161331
7.H	-2.991968	0.189227	3.167290
8.H	-0.509128	2.122373	3.279420
9.C	-2.121859	0.722454	-0.722046
10.H	-2.937908	0.225971	-0.180347
11.H	-2.485653	1.677660	-1.120935
12.H	-0.027048	-1.357198	0.271227
13.H	-1.303291	-1.096300	1.489260
14.C	-3.206707	1.221960	2.870417
15.H	-3.560124	1.771313	3.751763
16.H	-4.004321	1.217740	2.118521
17.C	-2.009497	3.975039	1.674352
18.H	-2.783885	4.001464	0.898641
19.H	-2.378271	4.514523	2.555033

TS: CSiP + BF_3
E = −2074.69
H = −1989.72
G = −2023.08
N_{imag} = 1,−311.123i cm^{-1}

1.C	0.302180	1.133521	0.097395
2.P	-1.510830	0.804924	0.273102
3.H	0.480922	2.212189	0.041999
4.H	-1.040357	-1.454490	-0.487486
5.F	0.252057	1.937414	2.903779
6.Si	-2.728808	1.621583	1.895659
7.H	-4.124541	3.180778	0.628107
8.F	-1.999240	1.912958	3.638818
9.C	-1.492765	-1.039957	0.424601
10.H	-0.909469	-1.382006	1.287842
11.H	-2.521751	-1.402763	0.518044
12.H	0.601660	0.675278	-0.856735
13.H	0.901126	0.706069	0.907754
14.C	-3.751612	3.206462	1.660201
15.H	-4.616890	3.175522	2.332388

16.H	-3.182245	4.124668	1.827079
17.B	-0.932050	2.561418	2.697982
18.F	-0.933288	3.909137	2.822581

PC: CSiP + BF_3
E = −2139.30
H = −2053.27
G = −2089.01
N_{imag} = 0

1.C	-0.350220	-0.641014	1.036450
2.P	-0.512490	1.079410	0.323276
3.H	0.366853	-0.619352	1.866020
4.H	-1.664423	0.262213	-1.671960
5.F	-1.448531	5.110187	1.926176
6.Si	-1.544354	2.124706	2.062531
7.H	-2.772021	0.226218	3.123184
8.F	-0.419267	2.248532	3.238690
9.C	-1.989548	0.847741	-0.802296
10.H	-2.831655	0.328337	-0.326733
11.H	-2.331420	1.823306	-1.169211
12.H	0.058893	-1.297511	0.257311
13.H	-1.299327	-1.064467	1.389748
14.C	-3.030054	1.245649	2.807761
15.H	-3.394013	1.791555	3.688868
16.H	-3.852589	1.186379	2.082394
17.B	-2.045023	4.014875	1.462399
18.F	-3.000215	4.209112	0.545043

TS: CGeP + BF_3
E = −2049.61
H = −1964.91
G = −1999.60
N_{imag} = 1,-297.494i cm^{-1}

1.C	0.638320	1.023079	0.280062
2.P	-1.176497	0.812426	0.027547
3.H	0.895853	2.085406	0.257967
4.H	-0.757288	-1.475370	-0.656466
5.F	0.284084	2.483998	2.791902
6.Ge	-2.578807	1.734071	1.514618
7.H	-4.714584	3.021772	0.963799
8.F	-1.863438	1.978414	3.592340
9.C	-1.324917	-1.026380	0.171629
10.H	-0.919536	-1.403939	1.117812
11.H	-2.377769	-1.313185	0.084845
12.H	1.124537	0.511406	-0.564431
13.H	0.992309	0.585842	1.219397
14.C	-3.797451	3.306648	1.484124
15.H	-4.003132	3.534288	2.534068
16.H	-3.290631	4.151571	1.013264
17.B	-1.029453	2.847264	2.731921
18.F	-1.277615	4.172416	2.938792

PC: CGeP + BF_3
E = −2092.85
H = −2007.96
G = −2043.40
N_{imag} = 0

1.C	-0.313108	-0.692295	1.138196
2.P	-0.393613	0.952744	0.260812
3.H	0.327175	-0.593719	2.022857
4.H	-1.419943	-0.046840	-1.716655

5.F	-1.331903	5.171068	1.840517
6.Ge	-1.600483	2.154795	1.882806
7.H	-2.948820	0.250098	2.944665
8.F	-0.468172	2.255191	3.259807
9.C	-1.796156	0.625025	-0.933650
10.H	-2.675929	0.160249	-0.470091
11.H	-2.096290	1.561910	-1.419165
12.H	0.156661	-1.416884	0.459467
13.H	-1.293820	-1.080586	1.441691
14.C	-3.194379	1.273291	2.641162
15.H	-3.524121	1.838243	3.520320
16.H	-4.004116	1.251524	1.902552
17.B	-1.963675	4.136875	1.303902
18.F	-2.847317	4.399127	0.338364

TS: $CSiPH_2$ + ethylene
E = −2454.63
H = −2337.11
G = −2368.42
N_{imag} = 1,-709.037i cm^{-1}

1.H	1.337569	-1.045387	-2.446114
2.H	-1.096693	-1.453541	-2.538278
3.H	-1.317745	0.008112	-3.604050
4.H	1.123895	0.427711	-3.546007
5.C	1.293067	-0.900702	1.997282
6.P	0.045466	-1.142061	0.625155
7.H	2.292372	-0.776961	1.561082
8.H	-1.581158	-2.000939	2.250111
9.H	-1.131015	0.227042	-1.817522
10.Si	-0.147251	0.965543	-0.307752
11.H	1.394325	2.166391	1.269145
12.H	0.783838	0.896064	-1.585265
13.C	-1.533422	-1.093028	1.634260
14.H	-1.609140	-0.219851	2.297583
15.H	-2.397512	-1.100427	0.958225
16.H	1.311035	-1.807652	2.617056
17.H	1.078068	-0.039287	2.643538
18.C	0.324107	2.251621	1.022189
19.H	0.139220	3.274695	0.668782
20.H	-0.239804	2.103104	1.954710
21.C	-0.743275	-0.450560	-2.792431
22.C	0.674053	-0.289889	-2.861701

TS: $CSiPH_2$ + acetylene
E = −225.33
H = −2152.65
G = −2185.66
N_{imag} = 1,-945.114i cm^{-1}

1.H	-0.570123	1.926607	3.805122
2.H	1.428137	-0.393763	3.762426
3.C	-0.037922	1.177089	3.245897
4.C	0.776532	0.216056	3.145963
5.C	1.472688	1.278707	-2.059690
6.P	-0.243291	0.632932	-1.726561
7.H	1.648782	2.158025	-1.427760
8.H	-0.184874	-0.792991	-3.716970
9.H	-0.422802	1.304706	1.535546
10.Si	-0.207452	0.123569	0.480483
11.H	-1.383842	-2.080134	0.201366
12.H	0.885342	-0.179108	1.899188
13.C	-0.227786	-0.994717	-2.638806

14.H	0.620954	-1.635771	-2.368237
15.H	-1.162079	-1.530928	-2.435291
16.H	1.539503	1.597857	-3.107677
17.H	2.258036	0.538432	-1.861168
18.C	-1.625098	-1.124305	0.687256
19.H	-1.803537	-1.314321	1.753459
20.H	-2.557129	-0.748884	0.241705

TS: $CGePH_2$ + ethylene

E = −2431.36

H = −2315.36

G = −2346.62

N_{imag} = 1,-794.624i cm^{-1}

1.H	1.266151	-1.622460	-2.606588
2.H	-1.214519	-1.582458	-2.694398
3.H	-1.142547	-0.081820	-3.776649
4.H	1.339930	-0.136471	-3.664501
5.C	1.575704	-0.913913	1.781257
6.P	-0.128936	-1.136795	1.034853
7.H	2.337610	-1.115631	1.018098
8.H	-1.104039	-1.228009	3.281531
9.H	-1.034851	0.384520	-1.696758
10.Ge	-0.050965	0.748940	-0.436644
11.H	0.170123	2.473797	1.511359
12.H	0.987423	0.047227	-1.880622
13.C	-1.165382	-0.504642	2.456433
14.H	-0.850937	0.479141	2.828964
15.H	-2.215335	-0.447513	2.142959
16.H	1.702367	-1.652264	2.584531
17.H	1.748472	0.087228	2.200005
18.C	-0.480655	2.394135	0.631545
19.H	-0.355843	3.288733	0.010639
20.H	-1.523346	2.341398	0.974342
21.C	-0.631139	-0.753541	-3.089722
22.C	0.770713	-0.698542	-2.917614

TS: $CGePH_2$ + acetylene

E = −2230.70

H = −2128.61

G = −2163.33

N_{imag} = 1,-822.027i cm^{-1}

1.H	-0.274396	2.063176	3.846775
2.H	1.718348	-0.338204	3.612223
3.C	0.213268	1.246750	3.350153
4.C	0.966123	0.268163	3.117826
5.C	1.412010	1.310670	-1.949712
6.P	-0.314007	0.627471	-1.763864
7.H	1.498219	2.229661	-1.356890
8.H	0.010793	-0.864010	-3.677747
9.H	-0.566475	1.272689	1.576079
10.Ge	-0.366763	0.053374	0.516748
11.H	-1.678158	-2.149788	0.130877
12.H	0.837129	-0.201222	1.894331
13.C	-0.127467	-1.031520	-2.601307
14.H	0.721966	-1.617857	-2.227652
15.H	-1.049514	-1.609991	-2.469020
16.H	1.577904	1.573121	-3.003036
17.H	2.193690	0.609039	-1.631602
18.C	-1.895566	-1.219467	0.669496
19.H	-2.075965	-1.450870	1.725320
20.H	-2.801137	-0.771186	0.241003

Table S15. Cartesian coordinates (in Å), energies (in kcal mol[−1]), and number of imaginary frequencies of all stationary points, computed at ZORA-BP86-D3(BJ)/TZ2P.

H_2
E (ZORA-BP86-D3(BJ)/TZ2P) = −155.32
E (ZORA-M06-2x/TZ2P//ZORA-BP86-D3(BJ)/TZ2P) = −210.93
H = −147.11
G = −156.41
N_{imag} = 0

1.H	0.000000	0.000000	-0.374824
2.H	0.000000	0.000000	0.374824

$H_3C–C–NMe_2$ (CCN)
E (ZORA-BP86-D3(BJ)/TZ2P) = −1723.98
E (ZORA-M06-2x/TZ2P//ZORA-BP86-D3(BJ)/TZ2P) = −2370.38
H = −1643.98
G = −1668.10
N_{imag} = 0

1.C	-0.067013	0.507614	0.576333
2.N	-1.305666	0.589521	0.151963
3.H	0.758058	0.332026	-1.524344
4.H	-1.916259	0.739344	2.130710
5.H	-2.539955	-0.277017	-1.340888
6.C	-1.843825	0.565115	-1.233706
7.H	-2.391785	1.496074	-1.429546
8.H	-1.037579	0.461391	-1.961158
9.C	-2.381692	0.729000	1.142778
10.H	-2.936096	1.663117	0.969275
11.H	-3.084762	-0.112718	1.057858
12.C	1.012392	0.366635	-0.447053
13.H	1.724361	1.190759	-0.287545
14.H	1.583123	-0.541054	-0.198474

$H_3C–Si–NMe_2$ (CSiN)
E (ZORA-BP86-D3(BJ)/TZ2P) = −1668.24
E (ZORA-M06-2x/TZ2P//ZORA-BP86-D3(BJ)/TZ2P) = −2292.88
H = −1590.60
G = −1615.47
N_{imag} = 0

1.Si	0.106059	0.506449	0.826932
2.N	-1.484595	0.603732	0.140641
3.H	1.082162	1.190991	-1.430152
4.H	-2.270667	0.775810	2.083652
5.H	-2.592870	-0.281353	-1.429627
6.C	-1.905743	0.563251	-1.255122
7.H	-2.442610	1.487520	-1.526152
8.H	-1.044477	0.453869	-1.919374
9.C	-2.624401	0.749655	1.045439
10.H	-3.176044	1.681004	0.836594
11.H	-3.326658	-0.092632	0.932658
12.C	1.182069	0.325575	-0.756219
13.H	2.239122	0.249847	-0.467647
14.H	0.928568	-0.578526	-1.331967

H_3C-Ge-NMe_2 (CGeN)
E (ZORA-BP86-D3(BJ)/TZ2P) = −1644.34
E (ZORA-M06-2x/TZ2P//ZORA-BP86-D3(BJ)/TZ2P) = −2272.81
H = −1567.22
G = −1593.14
N_{imag} = 0

1.Ge	0.139539	0.523327	0.854543
2.N	-1.560310	0.591900	0.115100
3.H	1.072831	1.183102	-1.520647
4.H	-2.362477	0.778319	2.049159
5.H	-2.626986	-0.332284	-1.460662
6.C	-1.954652	0.525261	-1.281787
7.H	-2.501581	1.437065	-1.579936
8.H	-1.079836	0.419728	-1.929540
9.C	-2.703289	0.733003	1.006887
10.H	-3.271608	1.652860	0.784580
11.H	-3.397225	-0.119188	0.903047
12.C	1.213000	0.321320	-0.852481
13.H	2.277299	0.254468	-0.593822
14.H	0.931696	-0.590075	-1.399242

H_3C-Ge-PMe_2 (CGeP)
E (ZORA-BP86-D3(BJ)/TZ2P) = −1570.58
E (ZORA-M06-2x/TZ2P//ZORA-BP86-D3(BJ)/TZ2P) = −2153.62
H = −1496.73
G = −1524.79
N_{imag} = 0

2.P	-0.990207	0.087194	0.247210
3.H	-1.561798	2.852339	-2.428368
4.H	-2.283016	-0.533757	2.187368
5.H	1.150697	-1.063995	-0.085365
6.C	0.291186	-0.814911	-0.720997
7.H	-0.123684	-1.738885	-1.144024
8.H	0.633604	-0.174482	-1.541335
9.C	-1.514667	-1.044023	1.594713
10.H	-1.939486	-1.971660	1.189865
11.H	-0.671611	-1.295183	2.251240
12.C	-0.843776	2.643010	-1.623777
13.H	-0.325579	3.581566	-1.385044
14.H	-0.110249	1.914838	-1.984125

H_3C-Ge-$AsMe_2$ (CGeAs)
E (ZORA-BP86-D3(BJ)/TZ2P) = −1543.95
E (ZORA-M06-2x/TZ2P//ZORA-BP86-D3(BJ)/TZ2P) = −2131.00
H = −1470.28
G = −1500.55
N_{imag} = 0

1.Ge	-1.927950	2.040204	-0.019932
2.As	-0.428039	0.236988	0.678832
3.H	-1.084598	1.805908	-2.449721
4.H	-2.251419	-0.576608	2.280248
5.H	0.719568	-1.597571	-0.697215
6.C	0.084147	-0.751455	-0.979602
7.H	-0.801523	-1.115060	-1.510408
8.H	0.652151	-0.075424	-1.625989
9.C	-1.690326	-1.080741	1.486126
10.H	-2.387236	-1.472941	0.738523
11.H	-1.113145	-1.903830	1.920334
12.C	-0.861015	2.544890	-1.663609
13.H	-1.148855	3.537979	-2.026859
14.H	0.222269	2.519538	-1.487011

H_3C–Sn–NMe_2 (CSnN)
E (ZORA-BP86-D3(BJ)/TZ2P) = –1623.54
E (ZORA-M06-2x/TZ2P//ZORA-BP86-D3(BJ)/TZ2P) = –2246.35
H = –1546.92
G = –1573.86
N_{imag} = 0

1.Sn	-1.942631	0.503368	1.127155
2.N	-1.371668	1.075115	-0.771744
3.H	0.429752	-1.002050	1.114344
4.H	-2.667629	2.734037	-0.700787
5.H	-0.887351	0.111120	-2.594630
6.C	-0.414267	0.421411	-1.644519
7.H	0.417763	1.101591	-1.905055
8.H	0.007310	-0.467873	-1.165182
9.C	-1.948744	2.261434	-1.383274
10.H	-1.170212	3.005653	-1.632369
11.H	-2.476767	2.014606	-2.322578
12.C	-0.625364	-1.288411	1.224322
13.H	-0.753303	-1.786601	2.193464
14.H	-0.878342	-2.001815	0.427826

TS: CCN + H_2
E (ZORA-BP86-D3(BJ)/TZ2P) = –1869.26
E (ZORA-M06-2x/TZ2P//ZORA-BP86-D3(BJ)/TZ2P) = –2561.83
H = –1780.55
G = –1805.13
N_{imag} = 1,–890i cm^{-1}

1.C	0.266216	0.666483	-0.073552
2.N	-0.563756	0.827965	0.960167
3.H	1.013437	-0.626889	-1.584435
4.H	0.083251	2.790374	1.240942
5.H	-0.762737	-0.753604	2.354758
6.C	-1.355253	-0.240737	1.577263
7.H	-2.253803	0.184773	2.040040
8.H	-1.660372	-0.970882	0.824141
9.C	-0.302816	1.961099	1.839552
10.H	-1.221598	2.268418	2.353469
11.H	0.453823	1.682604	2.594822
12.H	1.470643	0.918128	0.188046
13.H	2.090417	0.280502	0.690789
14.C	0.115019	-0.530408	-0.963705
15.H	-0.059311	-1.499557	-0.470495
16.H	-0.740030	-0.345789	-1.632553

PC: CCN + H_2
E (ZORA-BP86-D3(BJ)/TZ2P) = –1939.39
E (ZORA-M06-2x/TZ2P//ZORA-BP86-D3(BJ)/TZ2P) = –2645.22
H = –1844.23
G = –1687.8
N_{imag} = 0

1.C	-0.400434	-0.409180	1.134093
2.N	-0.909270	0.885700	0.708906
3.H	0.241830	-0.281610	2.015706
4.H	-1.137571	0.352346	-1.302264
5.H	-1.761511	2.650228	1.391732
6.C	-1.541525	1.647502	1.788873
7.H	-2.635155	0.070782	2.855951
8.H	-0.784792	1.780355	2.577078
9.C	-1.734657	0.777753	-0.484190
10.H	-2.631584	0.134767	-0.356627
11.H	-2.071892	1.776852	-0.790949

12.H	0.207056	-0.843657	0.328538
13.H	-1.193039	-1.145039	1.388038
14.C	-2.819061	1.055497	2.407094
15.H	-3.191682	1.717449	3.200013
16.H	-3.618424	0.946632	1.662693

TS: CSiN + H_2
E (ZORA-BP86-D3(BJ)/TZ2P) = −1800.22
E (ZORA-M06-2x/TZ2P//ZORA-BP86-D3(BJ)/TZ2P) = −2474.41
H = −1714.41
G = −1740.43
N_{imag} = 1,−1131i cm^{-1}

1.Si	0.512047	0.664252	-0.136269
2.N	-0.603219	0.862646	1.167552
3.H	0.853395	-1.007022	-1.928412
4.H	-0.148094	2.907187	1.361013
5.H	-0.770334	-0.506001	2.789214
6.C	-1.276554	-0.237105	1.842848
7.H	-2.319184	0.032473	2.081539
8.H	-1.294999	-1.127029	1.204395
9.C	-0.645175	2.107660	1.924006
10.H	-1.688852	2.411367	2.112989
11.H	-0.141109	2.015442	2.903834
12.H	1.988640	0.504425	0.276010
13.H	1.617910	-0.521051	0.573417
14.C	-0.031013	-0.652663	-1.382047
15.H	-0.528670	-1.520556	-0.934465
16.H	-0.717235	-0.196115	-2.106208

PC: CSiN + H_2
E (ZORA-BP86-D3(BJ)/TZ2P) = −1859.92
E (ZORA-M06-2x/TZ2P//ZORA-BP86-D3(BJ)/TZ2P) = −2542.66
H = −1771.33
G = −1797.62
N_{imag} = 0

1.C	-0.189975	-0.485619	0.923327
2.N	-0.913507	0.734702	0.605056
3.H	0.298472	-0.393019	1.902058
4.H	-0.771234	0.550655	-1.491636
5.H	-1.758197	3.105776	1.216517
6.Si	-1.498796	1.779456	1.855216
7.H	-2.928676	0.251341	3.222495
8.H	-0.404700	1.887767	2.867838
9.C	-1.532278	0.718024	-0.710569
10.H	-2.295779	-0.076883	-0.815688
11.H	-2.015875	1.682111	-0.914946
12.H	0.595089	-0.674588	0.171514
13.H	-0.844732	-1.378024	0.950603
14.C	-3.069803	1.224197	2.732398
15.H	-3.357538	1.952642	3.504327
16.H	-3.906885	1.131924	2.027138

TS: CGeN + H_2
E (ZORA-BP86-D3(BJ)/TZ2P) = −1762.43
E (ZORA-M06-2x/TZ2P//ZORA-BP86-D3(BJ)/TZ2P) = −2442.54
H = −1677.55
G = −1704.95
N_{imag} = 1,−1189i cm^{-1}

1.Ge	0.493660	0.704360	-0.182229
2.N	-0.801357	0.963348	1.132416
3.H	0.977992	-1.164328	-1.830635

4.H	-0.102129	2.904692	1.551872
5.H	-0.671440	-0.650660	2.536730
6.C	-1.360020	-0.211825	1.785600
7.H	-2.299386	0.058387	2.296204
8.H	-1.596931	-0.987902	1.048432
9.C	-0.509147	2.031614	2.077982
10.H	-1.435377	2.343042	2.588151
11.H	0.217157	1.731127	2.861404
12.H	1.968909	0.858247	0.309616
13.H	1.609369	-0.343056	0.795914
14.C	0.055623	-0.838906	-1.338801
15.H	-0.366032	-1.670396	-0.768761
16.H	-0.661270	-0.504822	-2.094996

PC: CGeN + H_2
E (ZORA-BP86-D3(BJ)/TZ2P) = −1813.31
E (ZORA-M06-2x/TZ2P//ZORA-BP86-D3(BJ)/TZ2P) = −2502.76
H = −1725.79
G = −1752.92
N_{imag} = 0

1.C	-0.179381	-0.432197	0.941924
2.N	-0.739129	0.854567	0.550661
3.H	0.407911	-0.326419	1.863867
4.H	-0.857769	0.423071	-1.504153
5.H	-1.868979	3.208802	1.315977
6.Ge	-1.535436	1.834215	1.924041
7.H	-2.930918	-0.003850	3.044886
8.H	-0.466490	1.951037	3.026062
9.C	-1.509081	0.764769	-0.682714
10.H	-2.363089	0.057441	-0.626138
11.H	-1.901914	1.752673	-0.957989
12.H	0.498886	-0.798348	0.153492
13.H	-0.943681	-1.220230	1.108247
14.C	-3.167015	0.986672	2.639771
15.H	-3.589450	1.604047	3.441579
16.H	-3.915444	0.878502	1.846610

TS: CGeP + H_2
E (ZORA-BP86-D3(BJ)/TZ2P) = −1708.95
E (ZORA-M06-2x/TZ2P//ZORA-BP86-D3(BJ)/TZ2P) = −2344.46
H = −1626.66
G = −1655.44
N_{imag} = 1,−1096i cm^{-1}

1.C	-0.087349	-0.643120	1.080452
2.P	-0.182817	1.123276	0.497163
3.H	0.484524	-0.683204	2.014615
4.H	-0.740084	0.329265	-1.749230
5.H	-0.967469	3.025689	2.925484
6.Ge	-1.777224	2.062453	1.951287
7.H	-2.370199	-0.224335	3.093888
8.H	-0.428377	1.896542	3.032008
9.C	-1.306036	0.863704	-0.974072
10.H	-2.208181	0.282951	-0.740646
11.H	-1.602452	1.838452	-1.379409
12.H	0.444463	-1.230584	0.319143
13.H	-1.072306	-1.099325	1.244095
14.C	-2.839901	0.762592	3.054765
15.H	-2.934963	1.163280	4.069743
16.H	-3.837314	0.675995	2.611445

PC: CGeP + H_2
E (ZORA-BP86-D3(BJ)/TZ2P) = −1753.79
E (ZORA-M06-2x/TZ2P//ZORA-BP86-D3(BJ)/TZ2P) = −2398.09
H = −1669.03
G = −1698.14
N_{imag} = 0

1.C	-0.048862	-0.577248	1.047027
2.P	-0.266008	1.155593	0.385975
3.H	0.594887	-0.549606	1.934686
4.H	-1.176910	0.293388	-1.717319
5.H	-2.101771	3.401809	1.678381
6.Ge	-1.600232	2.004328	2.101284
7.H	-2.809297	-0.131107	2.862334
8.H	-0.722845	2.174478	3.360039
9.C	-1.612596	0.834822	-0.867323
10.H	-2.446525	0.243956	-0.466242
11.H	-1.997574	1.791872	-1.240096
12.H	0.457413	-1.181736	0.283010
13.H	-0.997932	-1.064318	1.306679
14.C	-3.148669	0.857376	2.534116
15.H	-3.731018	1.316533	3.341081
16.H	-3.793159	0.741475	1.655925

TS: CGeAs + H_2
E (ZORA-BP86-D3(BJ)/TZ2P) = −1687.11
E (ZORA-M06-2x/TZ2P//ZORA-BP86-D3(BJ)/TZ2P) = −2323.04
H = −1605.51
G = −1635.78
N_{imag} = 1,−1089i cm^{-1}

1.C	-0.048647	-0.710523	1.078524
2.As	-0.143329	1.171801	0.420268
3.H	0.544702	-0.733111	1.998264
4.H	-0.932883	0.232652	-1.828917
5.H	-1.001944	3.017983	2.993209
6.Ge	-1.829590	2.097930	1.977741
7.H	-2.332429	-0.247699	3.054829
8.H	-0.430524	1.921020	3.000602
9.C	-1.455750	0.796200	-1.046816
10.H	-2.311241	0.211176	-0.692228
11.H	-1.805574	1.747043	-1.462501
12.H	0.447011	-1.313632	0.308063
13.H	-1.044836	-1.122182	1.271926
14.C	-2.820690	0.730818	3.071501
15.H	-2.887744	1.088188	4.104873
16.H	-3.831587	0.641075	2.659831

PC: CGeAs + H_2
E (ZORA-BP86-D3(BJ)/TZ2P) = −1731.01
E (ZORA-M06-2x/TZ2P//ZORA-BP86-D3(BJ)/TZ2P) = −2375.41
H = −1646.86
G = −1677.45
N_{imag} = 0

1.C	-0.053424	-0.677418	1.079884
2.As	-0.200692	1.181084	0.350431
3.H	0.576072	-0.660463	1.976140
4.H	-1.283001	0.241304	-1.773673
5.H	-2.156742	3.421869	1.740989
6.Ge	-1.631291	2.025299	2.142121
7.H	-2.808817	-0.142805	2.853676
8.H	-0.779452	2.196579	3.419754
9.C	-1.694641	0.803282	-0.927590

10.H	-2.493039	0.219188	-0.457762
11.H	-2.098498	1.752680	-1.295518
12.H	0.428583	-1.302724	0.319786
13.H	-1.035112	-1.096124	1.325427
14.C	-3.163143	0.844030	2.536756
15.H	-3.764743	1.279509	3.342818
16.H	-3.792841	0.730081	1.647752

TS: CSnN + H_2
E (ZORA-BP86-D3(BJ)/TZ2P) = −1730.86
E (ZORA-M06-2x/TZ2P//ZORA-BP86-D3(BJ)/TZ2P) = −2402.19
H = −1646.95
G = −1675.14
N_{imag} = 1,-1168i cm^{-1}

1.C	-0.221472	-0.382922	0.987165
2.N	-0.595094	0.964628	0.575198
3.H	0.196664	-0.367218	2.001378
4.H	-0.217987	0.660780	-1.466877
5.H	-0.834066	3.334152	2.580693
6.Sn	-1.779938	2.037564	1.917067
7.H	-2.298686	-0.192025	3.451308
8.H	-0.329384	2.124404	3.209845
9.C	-1.044352	0.991276	-0.813745
10.H	-1.909566	0.329907	-1.026894
11.H	-1.316083	2.012837	-1.109369
12.H	0.555702	-0.766345	0.302590
13.H	-1.054875	-1.115857	0.966357
14.C	-2.843070	0.749659	3.345938
15.H	-2.884550	1.272621	4.305571
16.H	-3.851344	0.571336	2.964117

PC: CSnN + H_2
E (ZORA-BP86-D3(BJ)/TZ2P) = −1777.92
E (ZORA-M06-2x/TZ2P//ZORA-BP86-D3(BJ)/TZ2P) = −2461.21
H = −1692.08
G = −1720.80
N_{imag} = 0

1.C	-0.016066	-0.401563	0.826123
2.N	-0.598110	0.879777	0.450919
3.H	0.565912	-0.302621	1.752770
4.H	-0.685623	0.464018	-1.609945
5.H	-1.882619	3.473966	1.287248
6.Sn	-1.501695	1.941955	1.975827
7.H	-3.029924	-0.044968	3.086431
8.H	-0.308147	2.050448	3.212572
9.C	-1.353071	0.785341	-0.791070
10.H	-2.193160	0.059271	-0.755142
11.H	-1.763939	1.766399	-1.066650
12.H	0.675256	-0.743000	0.035677
13.H	-0.762492	-1.210916	0.974334
14.C	-3.292471	0.945339	2.699958
15.H	-3.750653	1.535518	3.501197
16.H	-4.007965	0.836403	1.878208

Table S16. Cartesian coordinates (in Å), energies (in kcal mol^{-1}), and number of imaginary frequencies of all stationary points, computed at COSMO(toluene)ZORA-BP86/TZ2P.

H_2
E = -155.30
H = -147.07
G = -156.36
N_{imag} = 0

1.H	0.000000	0.000000	-0.375060
2.H	0.000000	0.000000	0.375060

H_3C–C–NMe_2 (CCN)
E = -1718.39
H = -1638.35
G = -1662.60
N_{imag} = 0

1.C	-0.064625	0.507243	0.572445
2.N	-1.304978	0.589676	0.154962
3.H	0.759875	0.331718	-1.524523
4.H	-1.933153	0.741212	2.137513
5.H	-2.538630	-0.276825	-1.334410
6.C	-1.842380	0.565219	-1.230656
7.H	-2.390723	1.496308	-1.422969
8.H	-1.039187	0.461781	-1.960912
9.C	-2.385951	0.729401	1.143750
10.H	-2.938877	1.662603	0.965184
11.H	-3.087237	-0.112364	1.053830
12.C	1.018237	0.365924	-0.450526
13.H	1.731143	1.190329	-0.293384
14.H	1.589789	-0.542415	-0.204102

H_3C–Si–NMe_2 (CSiN)
E = -1657.36
H = -1679.89
G = -1604.87
N_{imag} = 0

1.Si	0.105823	0.504914	0.812420
2.N	-1.489099	0.603648	0.137745
3.H	1.104222	1.190500	-1.431137
4.H	-2.271449	0.776198	2.086729
5.H	-2.606404	-0.280367	-1.429808
6.C	-1.917957	0.563769	-1.258612
7.H	-2.455992	1.488233	-1.526408
8.H	-1.060086	0.454288	-1.927816
9.C	-2.627053	0.750254	1.048912
10.H	-3.179121	1.681732	0.842312
11.H	-3.330353	-0.091343	0.938509
12.C	1.197085	0.324395	-0.757207
13.H	2.249873	0.249693	-0.451803
14.H	0.950427	-0.580721	-1.334182

H_3C–Ge–NMe_2 (CGeN)
E = -1632.89
H = -1555.95
G = -1581.99
N_{imag} = 0

1.Ge	0.139772	0.522329	0.842924
2.N	-1.564620	0.591685	0.111260
3.H	1.095262	1.182973	-1.521744
4.H	-2.360988	0.778476	2.051585

5.H	-2.642134	-0.330998	-1.461559
6.C	-1.967729	0.525636	-1.285956
7.H	-2.515834	1.437752	-1.581099
8.H	-1.096671	0.419430	-1.939373
9.C	-2.705206	0.733408	1.010175
10.H	-3.274548	1.653343	0.790872
11.H	-3.400437	-0.118094	0.909507
12.C	1.227930	0.320425	-0.852976
13.H	2.288285	0.254460	-0.576952
14.H	0.953320	-0.592015	-1.401464

H_3C–Ge–PMe_2 (CGeP)

E = −1557.28
H = −1482.99
G = −1512.59
N_{imag} = 0

1.Ge	-1.900563	2.086889	0.012017
2.P	-0.979891	0.071988	0.239910
3.H	-1.584694	2.877731	-2.430449
4.H	-2.286414	-0.528876	2.185763
5.H	1.154626	-1.096471	-0.069125
6.C	0.303340	-0.846011	-0.715359
7.H	-0.117418	-1.770609	-1.131381
8.H	0.658263	-0.216985	-1.539415
9.C	-1.518533	-1.044697	1.597054
10.H	-1.945155	-1.972966	1.195419
11.H	-0.677120	-1.295523	2.255910
12.C	-0.854493	2.675180	-1.634761
13.H	-0.358305	3.623156	-1.385514
14.H	-0.106996	1.967289	-2.007287

H_3C–Ge–$AsMe_2$ (CGeAs)

E = −1529.82
H = −1456.32
G = −1486.99
N_{imag} = 0

1.Ge	-1.925588	2.047761	-0.042198
2.As	-0.440110	0.222404	0.660479
3.H	-1.169518	1.922741	-2.499844
4.H	-2.252833	-0.574832	2.285286
5.H	0.751216	-1.632367	-0.648091
6.C	0.110883	-0.802182	-0.966140
7.H	-0.760795	-1.190463	-1.502799
8.H	0.684256	-0.136910	-1.619685
9.C	-1.698684	-1.087294	1.491149
10.H	-2.401195	-1.489982	0.754066
11.H	-1.115695	-1.904102	1.930549
12.C	-0.875788	2.598629	-1.680405
13.H	-1.132590	3.624693	-1.969881
14.H	0.210469	2.513782	-1.548768

H_3C–Sn–NMe_2 (CSnN)

E = −1611.38
H = −1535.00
G = −1562.13
N_{imag} = 0

1.Sn	-1.934438	0.496146	1.124075
2.N	-1.367275	1.075278	-0.779527
3.H	0.432721	-1.020844	1.139397
4.H	-2.669226	2.732740	-0.696100
5.H	-0.885700	0.122386	-2.613267

6.C	-0.410623	0.429253	-1.662553
7.H	0.418939	1.112580	-1.924482
8.H	0.016919	-0.462127	-1.191021
9.C	-1.951376	2.264848	-1.383421
10.H	-1.177173	3.014002	-1.632952
11.H	-2.483041	2.023023	-2.322431
12.C	-0.624932	-1.300967	1.241628
13.H	-0.768992	-1.783709	2.216807
14.H	-0.877256	-2.021025	0.450820

TS: CCN + H_2
E = −1860.75
H = −1772.48
G = −1798.15
$\boldsymbol{N}_{\mathbf{imag}}$ = 1,−820i cm^{-1}

1.C	0.331001	0.594095	0.000074
2.N	-0.435300	0.757209	1.062711
3.H	0.883245	-0.573519	-1.679162
4.H	-0.900820	2.527633	2.111893
5.H	-2.401628	0.509904	1.766197
6.C	-1.544082	-0.110231	1.477155
7.H	-1.841167	-0.770229	0.660189
8.H	-1.241498	-0.715906	2.345006
9.C	-0.086865	1.794220	2.034700
10.H	0.072354	1.340304	3.022891
11.H	0.827823	2.295684	1.707640
12.H	1.523499	0.795384	0.158297
13.H	2.431359	0.169202	0.530862
14.C	0.015781	-0.451244	-1.019537
15.H	-0.245686	-1.442104	-0.617847
16.H	-0.836619	-0.111326	-1.628172

PC: CCN + H_2
E = −1929.25
H = −1834.24
G = −1857.84
$\boldsymbol{N}_{\mathbf{imag}}$ = 0

1.C	-0.395843	-0.411444	1.132060
2.N	-0.904755	0.888279	0.707084
3.H	0.247705	-0.286474	2.013285
4.H	-1.135828	0.349669	-1.306400
5.H	-1.766362	2.650088	1.395073
6.C	-1.544541	1.647210	1.791124
7.H	-2.641834	0.072680	2.863925
8.H	-0.789244	1.779935	2.580887
9.C	-1.732576	0.777762	-0.489304
10.H	-2.628449	0.135379	-0.358698
11.H	-2.070410	1.775979	-0.798786
12.H	0.209348	-0.846830	0.325084
13.H	-1.189998	-1.144389	1.385862
14.C	-2.822982	1.056263	2.410911
15.H	-3.190056	1.723101	3.202848
16.H	-3.625890	0.949171	1.669730

TS: CSiN + H_2
E = −1787.98
H = −1702.12
G = −1785.61
$\boldsymbol{N}_{\mathbf{imag}}$ = 1,−1168i cm^{-1}

1.Si	0.511756	0.653885	-0.132911
2.N	-0.608118	0.857751	1.170173

3.H	0.867349	-1.002962	-1.934252
4.H	-0.167991	2.911576	1.344054
5.H	-0.760976	-0.496687	2.809457
6.C	-1.273781	-0.239604	1.863273
7.H	-2.315344	0.029238	2.107895
8.H	-1.295317	-1.136914	1.234794
9.C	-0.650315	2.111121	1.918829
10.H	-1.694103	2.407349	2.118764
11.H	-0.134030	2.031699	2.893359
12.H	1.986869	0.472740	0.288803
13.H	1.595774	-0.524191	0.568401
14.C	-0.024273	-0.647087	-1.399973
15.H	-0.538849	-1.514620	-0.970539
16.H	-0.691918	-0.174423	-2.131459

PC: CSiN + H_2
E = −1847.72
H = −1759.37
G = −1785.61
N_{imag} = 0

1.C	-0.182521	-0.487496	0.917578
2.N	-0.904232	0.738902	0.600443
3.H	0.307999	-0.399460	1.895880
4.H	-0.764758	0.546934	-1.498393
5.H	-1.765461	3.101055	1.220632
6.Si	-1.508282	1.772309	1.857605
7.H	-2.944627	0.257359	3.241927
8.H	-0.413120	1.884753	2.870160
9.C	-1.525866	0.717474	-0.718148
10.H	-2.288404	-0.078310	-0.820396
11.H	-2.010456	1.680213	-0.927331
12.H	0.600502	-0.677570	0.163995
13.H	-0.839659	-1.377820	0.943515
14.C	-3.079959	1.226665	2.742864
15.H	-3.351493	1.966920	3.510076
16.H	-3.924078	1.138532	2.045242

TS: CGeN + H_2
E = −1750.38
H = −1665.53
G = −1692.91
N_{imag} = 1,−1233i cm^{-1}

1.Ge	0.498919	0.690487	-0.178624
2.N	-0.802942	0.952369	1.139324
3.H	0.985405	-1.168086	-1.836340
4.H	-0.127284	2.912743	1.529894
5.H	-0.661722	-0.629965	2.581985
6.C	-1.351363	-0.217827	1.817150
7.H	-2.296068	0.050555	2.319420
8.H	-1.574348	-1.013976	1.096963
9.C	-0.521252	2.041896	2.069696
10.H	-1.449997	2.348305	2.578610
11.H	0.210242	1.763584	2.855869
12.H	1.976748	0.793713	0.342474
13.H	1.609533	-0.362417	0.787798
14.C	0.056071	-0.828494	-1.366980
15.H	-0.406124	-1.658722	-0.826476
16.H	-0.627272	-0.460278	-2.138529

PC: CGeN + H_2
E = −1801.23
H = −1713.91
G = −1741.06
N_{imag} = 0

1.C	-0.167500	-0.437328	0.931008
2.N	-0.737799	0.851500	0.547225
3.H	0.413156	-0.336575	1.857901
4.H	-0.836396	0.431268	-1.514473
5.H	-1.877081	3.198237	1.315254
6.Ge	-1.551678	1.822470	1.927859
7.H	-2.960922	0.008215	3.081679
8.H	-0.475316	1.942527	3.023941
9.C	-1.498167	0.762052	-0.696704
10.H	-2.344829	0.046290	-0.651714
11.H	-1.898926	1.747443	-0.970218
12.H	0.518665	-0.790120	0.143070
13.H	-0.925605	-1.232887	1.084298
14.C	-3.186447	0.995836	2.663472
15.H	-3.584345	1.635013	3.461345
16.H	-3.947787	0.890811	1.882182

TS: CGeP + H_2
E = −1694.02
H = −1611.75
G = −1640.68
N_{imag} = 1,−1140i cm^{-1}

1.C	-0.056447	-0.669570	1.048961
2.P	-0.202280	1.104762	0.500022
3.H	0.522587	-0.712565	1.978935
4.H	-0.699148	0.357435	-1.772745
5.H	-0.977344	3.023466	2.906219
6.Ge	-1.799749	2.034836	1.965482
7.H	-2.440757	-0.213613	3.165354
8.H	-0.451406	1.904719	3.043693
9.C	-1.292129	0.862787	-0.997924
10.H	-2.189078	0.261897	-0.798557
11.H	-1.595332	1.842797	-1.386009
12.H	0.489924	-1.223570	0.272588
13.H	-1.026531	-1.158455	1.206838
14.C	-2.879305	0.787159	3.112653
15.H	-2.942071	1.215348	4.119265
16.H	-3.886114	0.725410	2.686383

PC: CGeP + H_2
E = −1738.60
H = −1654.04
G = −1683.33
N_{imag} = 0

1.C	-0.017987	-0.594476	1.020983
2.P	-0.286572	1.142931	0.393402
3.H	0.623941	-0.563614	1.910094
4.H	-1.116878	0.305615	-1.748413
5.H	-2.109810	3.391903	1.681908
6.Ge	-1.630980	1.987068	2.113700
7.H	-2.880673	-0.105377	2.940638
8.H	-0.733623	2.167871	3.359191
9.C	-1.590030	0.825636	-0.904692
10.H	-2.428241	0.216351	-0.542245
11.H	-1.974901	1.784345	-1.273868
12.H	0.509996	-1.163941	0.244396

13.H	-0.950505	-1.118528	1.267895
14.C	-3.199588	0.884646	2.596505
15.H	-3.748024	1.381578	3.405548
16.H	-3.866667	0.769192	1.735140

TS: CGeAs + H_2
E = −1671.40
H = −1589.83
G = −1620.24
N_{imag} = 1,−1130i cm^{-1}

1.C	-0.019744	-0.741514	1.043923
2.As	-0.163010	1.152683	0.425404
3.H	0.589422	-0.768899	1.953588
4.H	-0.879299	0.267854	-1.864990
5.H	-1.019145	3.022163	2.971479
6.Ge	-1.856546	2.068330	1.992627
7.H	-2.401668	-0.236247	3.145268
8.H	-0.460420	1.936110	3.015223
9.C	-1.435621	0.795856	-1.080685
10.H	-2.289009	0.184265	-0.768421
11.H	-1.791276	1.753324	-1.476690
12.H	0.481908	-1.312263	0.252436
13.H	-1.001515	-1.184797	1.241922
14.C	-2.861012	0.756638	3.142311
15.H	-2.896552	1.148399	4.165129
16.H	-3.881472	0.686651	2.750422

PC: CGeAs + H_2
E = −1715.08
H = −1631.15
G = −1661.92
N_{imag} = 0

1.C	-0.018604	-0.698277	1.049451
2.As	-0.221295	1.170871	0.360727
3.H	0.615955	-0.680815	1.942403
4.H	-1.216634	0.247490	-1.807319
5.H	-2.167308	3.414253	1.746682
6.Ge	-1.665024	2.009425	2.157397
7.H	-2.887165	-0.113815	2.942002
8.H	-0.792816	2.194911	3.422005
9.C	-1.668575	0.792667	-0.970324
10.H	-2.478133	0.193332	-0.540272
11.H	-2.067142	1.743419	-1.341223
12.H	0.481173	-1.287089	0.271283
13.H	-0.984096	-1.156460	1.289035
14.C	-3.219980	0.874869	2.607513
15.H	-3.787172	1.351142	3.416101
16.H	-3.873558	0.759166	1.735972

TS: CSnN + H_2
E = −1718.47
H = −1634.72
G = −1663.04
N_{imag} = 1,−1225i cm^{-1}

1.C	-0.201413	-0.396130	0.967202
2.N	-0.580100	0.960264	0.575399
3.H	0.218814	-0.397935	1.980862
4.H	-0.224301	0.669067	-1.476998
5.H	-0.830247	3.323615	2.575970
6.Sn	-1.785013	2.022265	1.927453
7.H	-2.317678	-0.178966	3.515109

8.H	-0.322761	2.138153	3.214846
9.C	-1.044034	0.995137	-0.813045
10.H	-1.909997	0.333716	-1.020057
11.H	-1.321066	2.017830	-1.100507
12.H	0.575291	-0.767978	0.275262
13.H	-1.033375	-1.129345	0.935949
14.C	-2.861881	0.756918	3.366015
15.H	-2.930537	1.312891	4.305755
16.H	-3.859122	0.564748	2.961925

PC: CSnN + H_2
E = −1765.48
H = −1679.87
G = −1708.56
N_{imag} = 0

1.C	-0.000559	-0.404997	0.813040
2.N	-0.594054	0.877877	0.446287
3.H	0.573976	-0.311924	1.745072
4.H	-0.664372	0.473991	-1.621578
5.H	-1.887567	3.461609	1.285603
6.Sn	-1.520061	1.928625	1.982760
7.H	-3.066752	-0.033069	3.124059
8.H	-0.314898	2.036484	3.210060
9.C	-1.341197	0.781822	-0.804769
10.H	-2.170970	0.044502	-0.778974
11.H	-1.764235	1.758580	-1.077686
12.H	0.699217	-0.732235	0.023563
13.H	-0.740153	-1.222240	0.946819
14.C	-3.317460	0.955820	2.726053
15.H	-3.751097	1.568822	3.524281
16.H	-4.044663	0.851366	1.913947

Table S17. Cartesian coordinates (in Å), energies (in kcal mol^{-1}), and number of imaginary frequencies of all stationary points, computed at COSMO(water)ZORA-BP86/TZ2P.

H_2
E = −155.35
H = −147.12
G = −156.41
N_{imag} = 0

1.H	0.000000	0.000000	-0.375277
2.H	0.000000	0.000000	0.375277

H_3C−C−NMe_2 (CCN)
E = −1722.44
H = −1642.34
G = −1667.43
N_{imag} = 0

1.C	-0.062439	0.506169	0.570567
2.N	-1.303274	0.589667	0.159440
3.H	0.751788	0.317525	-1.522297
4.H	-1.950841	0.740025	2.143386
5.H	-2.528107	-0.279866	-1.328162
6.C	-1.837417	0.566124	-1.225729
7.H	-2.391649	1.494609	-1.409892
8.H	-1.035585	0.471186	-1.957479
9.C	-2.389781	0.729728	1.143352
10.H	-2.938600	1.663168	0.959139
11.H	-3.090242	-0.110467	1.043982
12.C	1.020646	0.364428	-0.453984
13.H	1.725960	1.198746	-0.311895
14.H	1.603223	-0.536395	-0.204529

H_3C−Si−NMe_2 (CSiN)
E = −1658.57
H = −1581.19
G = −1606.22
N_{imag} = 0

1.Si	0.105737	0.504226	0.800865
2.N	-1.490268	0.603767	0.138187
3.H	1.109053	1.191345	-1.430199
4.H	-2.274376	0.776792	2.088996
5.H	-2.608992	-0.280227	-1.427858
6.C	-1.920546	0.563810	-1.259395
7.H	-2.458382	1.488503	-1.524725
8.H	-1.063429	0.454171	-1.929318
9.C	-2.628622	0.750536	1.050628
10.H	-3.180028	1.681605	0.842746
11.H	-3.331078	-0.091060	0.939214
12.C	1.202684	0.323899	-0.758497
13.H	2.253679	0.249148	-0.446814
14.H	0.954485	-0.581323	-1.334174

H_3C−Ge−NMe_2 (CGeN)
E = −1633.54
H = −1556.71
G = −1582.81
N_{imag} = 0

1.Ge	0.140479	0.521680	0.835982
2.N	-1.564290	0.591748	0.111269
3.H	1.097468	1.184581	-1.519864
4.H	-2.362217	0.779118	2.053014

5.H	-2.643212	-0.331386	-1.460384
6.C	-1.969141	0.525430	-1.287133
7.H	-2.517962	1.437307	-1.580051
8.H	-1.099004	0.419665	-1.941716
9.C	-2.705716	0.733686	1.011335
10.H	-3.274471	1.653207	0.790767
11.H	-3.400141	-0.117865	0.909837
12.C	1.230946	0.320176	-0.853865
13.H	2.289931	0.253435	-0.572736
14.H	0.953733	-0.591974	-1.401255

H_3C–Ge–PMe_2 (CGeP)
E = −1558.19
H = −1484.01
G = −1513.60
N_{imag} = 0

1.Ge	-1.892870	2.083171	0.008789
2.P	-0.986521	0.064752	0.235976
3.H	-1.585611	2.889003	-2.426336
4.H	-2.287783	-0.531920	2.189091
5.H	1.155106	-1.080146	-0.060171
6.C	0.305637	-0.841442	-0.712859
7.H	-0.108540	-1.771946	-1.121664
8.H	0.656599	-0.214030	-1.539670
9.C	-1.516562	-1.043470	1.601037
10.H	-1.933815	-1.976912	1.202098
11.H	-0.669399	-1.283037	2.256392
12.C	-0.852544	2.675527	-1.636199
13.H	-0.347956	3.617383	-1.380378
14.H	-0.113143	1.961824	-2.014166

H_3C–Ge–$AsMe_2$ (CGeAs)
E = −1530.95
H = −1457.58
G = −1488.22
N_{imag} = 0

1.Ge	-1.924568	2.025755	-0.050101
2.As	-0.409137	0.221911	0.683636
3.H	-1.128298	1.871236	-2.485371
4.H	-2.218384	-0.582724	2.312019
5.H	0.703443	-1.658245	-0.654241
6.C	0.109706	-0.790030	-0.961821
7.H	-0.772716	-1.121987	-1.518340
8.H	0.724601	-0.138811	-1.591532
9.C	-1.694194	-1.080313	1.488357
10.H	-2.420839	-1.434037	0.749421
11.H	-1.127301	-1.930105	1.884881
12.C	-0.880437	2.584761	-1.683144
13.H	-1.183498	3.589103	-2.002651
14.H	0.205653	2.555363	-1.527393

H_3C–Sn–NMe_2 (CSnN)
E = −1612.11
H = −1535.90
G = −1563.12
N_{imag} = 0

1.Sn	-1.932643	0.492861	1.126083
2.N	-1.366382	1.074469	-0.779438
3.H	0.432289	-1.019273	1.141751
4.H	-2.669183	2.732733	-0.694941
5.H	-0.885001	0.122644	-2.615448

6.C	-0.409416	0.429531	-1.665013
7.H	0.419262	1.113591	-1.927752
8.H	0.019794	-0.461818	-1.194658
9.C	-1.951759	2.265068	-1.383018
10.H	-1.178165	3.014617	-1.633259
11.H	-2.484151	2.023783	-2.321758
12.C	-0.624294	-1.303377	1.243567
13.H	-0.772552	-1.782754	2.219857
14.H	-0.879252	-2.020493	0.451000

TS: CCN + H_2
E = −1866.55
H = −1778.90
G = −1805.07
N_{imag} = 1,−1168i cm^{-1}

1.C	0.243588	0.566604	-0.025731
2.N	-0.440588	0.675040	1.079715
3.H	0.897302	-0.680903	-1.616181
4.H	0.625345	2.413617	1.576011
5.H	-1.107825	-0.669483	2.561122
6.C	-1.450418	-0.266455	1.599362
7.H	-2.389970	0.275684	1.760446
8.H	-1.618109	-1.080538	0.893554
9.C	-0.230958	1.844408	1.946213
10.H	-1.128783	2.474969	1.934169
11.H	-0.049231	1.510025	2.974982
12.H	1.535225	1.066824	-0.002413
13.H	2.561181	1.102253	0.138920
14.C	0.045317	-0.604400	-0.928011
15.H	-0.079466	-1.573511	-0.421101
16.H	-0.856925	-0.436583	-1.537683

PC: CCN + H_2
E = −1930.30
H = −1835.36
G = −1858.92
N_{imag} = 0

1.C	-0.396020	-0.410522	1.133325
2.N	-0.894270	0.896329	0.704397
3.H	0.245764	-0.290257	2.016308
4.H	-1.142683	0.345932	-1.307176
5.H	-1.768189	2.653425	1.398029
6.C	-1.542538	1.650867	1.792206
7.H	-2.635946	0.068338	2.856133
8.H	-0.790370	1.782446	2.584888
9.C	-1.733144	0.778864	-0.488307
10.H	-2.624885	0.136117	-0.343088
11.H	-2.075889	1.774809	-0.799176
12.H	0.205120	-0.853083	0.327469
13.H	-1.199553	-1.131701	1.385582
14.C	-2.819617	1.052269	2.405007
15.H	-3.189150	1.716898	3.197613
16.H	-3.620344	0.945649	1.661472

TS: CSiN + H_2
E = −1788.66
H = −1703.04
G = −1729.09
N_{imag} = 1,−1254i cm^{-1}

1.Si	0.501639	0.652604	-0.138495
2.N	-0.638335	0.867488	1.148446

3.H	0.882596	−1.013922	−1.922333
4.H	−0.184354	2.919224	1.338469
5.H	−0.738885	−0.507194	2.777400
6.C	−1.284355	−0.237359	1.853536
7.H	−2.314347	0.034801	2.138013
8.H	−1.333557	−1.126538	1.215028
9.C	−0.645746	2.110682	1.919311
10.H	−1.679957	2.404924	2.163995
11.H	−0.092466	2.014252	2.871497
12.H	1.974072	0.490246	0.304281
13.H	1.587552	−0.502437	0.591289
14.C	−0.016216	−0.656775	−1.401113
15.H	−0.534109	−1.520904	−0.968710
16.H	−0.676665	−0.188730	−2.142054

PC: CSiN + H_2
E = −1848.37
H = −1760.23
G = −1786.46
N_{imag} = 0

1.C	−0.186232	−0.477522	0.927317
2.N	−0.861246	0.775060	0.591537
3.H	0.328258	−0.386547	1.892966
4.H	−0.797355	0.507341	−1.504016
5.H	−1.761962	3.116003	1.230550
6.Si	−1.499875	1.783988	1.858800
7.H	−2.922345	0.228335	3.204959
8.H	−0.411343	1.902923	2.878015
9.C	−1.528327	0.727569	−0.708535
10.H	−2.315613	−0.048300	−0.757943
11.H	−1.991650	1.696803	−0.935167
12.H	0.569743	−0.720000	0.162238
13.H	−0.881890	−1.335671	0.989344
14.C	−3.070534	1.201739	2.717644
15.H	−3.360267	1.929047	3.490382
16.H	−3.903777	1.109695	2.007559

TS: CGeN + H_2
E = −1751.39
H = −1666.75
G = −1694.29
N_{imag} = 1,−1285i cm^{-1}

1.Ge	0.474821	0.701945	−0.199642
2.N	−0.832401	0.972348	1.121648
3.H	1.006181	−1.174210	−1.816686
4.H	−0.102895	2.907064	1.556774
5.H	−0.671648	−0.645208	2.523287
6.C	−1.378585	−0.208841	1.788636
7.H	−2.304074	0.060017	2.324594
8.H	−1.631936	−0.985134	1.056951
9.C	−0.495236	2.025218	2.079809
10.H	−1.397934	2.330121	2.633638
11.H	0.258561	1.702793	2.826767
12.H	1.941643	0.859026	0.341667
13.H	1.605369	−0.287500	0.813701
14.C	0.069005	−0.855489	−1.348230
15.H	−0.365621	−1.686381	−0.786503
16.H	−0.629148	−0.520933	−2.121825

PC: CGeN + H_2
E = −1802.23
H = −1715.06
G = −1742.23
N_{imag} = 0

1.C	-0.172118	-0.430915	0.938739
2.N	-0.713976	0.871053	0.542052
3.H	0.420297	-0.333418	1.858452
4.H	-0.861959	0.409224	-1.511274
5.H	-1.872338	3.207512	1.320373
6.Ge	-1.548298	1.830130	1.930486
7.H	-2.940736	-0.008945	3.054178
8.H	-0.472064	1.952895	3.026671
9.C	-1.502058	0.767934	-0.688492
10.H	-2.356740	0.066373	-0.608829
11.H	-1.894678	1.753257	-0.973541
12.H	0.493922	-0.813331	0.147837
13.H	-0.951424	-1.200373	1.110863
14.C	-3.176156	0.979204	2.642901
15.H	-3.583837	1.609891	3.442385
16.H	-3.928814	0.874259	1.853324

TS: CGeP + H_2
E = −1694.90
H = −1612.77
G = −1641.75
N_{imag} = 1,−1189i cm^{-1}

1.C	-0.047514	-0.672328	1.045300
2.P	-0.209005	1.099411	0.499732
3.H	0.536619	-0.712879	1.972218
4.H	-0.702810	0.353568	-1.770524
5.H	-0.972401	3.023696	2.906033
6.Ge	-1.796045	2.028164	1.973402
7.H	-2.451755	-0.212011	3.168722
8.H	-0.448813	1.904530	3.053284
9.C	-1.295887	0.862071	-0.998004
10.H	-2.193282	0.263657	-0.794526
11.H	-1.596773	1.842901	-1.385888
12.H	0.497004	-1.220285	0.263497
13.H	-1.015272	-1.164182	1.206754
14.C	-2.886654	0.790227	3.114172
15.H	-2.952587	1.221999	4.119088
16.H	-3.889549	0.734051	2.678328

PC: CGeP + H_2
E = −1739.40
H = −1655.00
G = −1684.49
N_{imag} = 0

1.C	-0.009404	-0.594288	1.020353
2.P	-0.296489	1.138457	0.395084
3.H	0.635519	-0.557716	1.907059
4.H	-1.115505	0.301712	-1.748168
5.H	-2.098961	3.395948	1.690654
6.Ge	-1.635816	1.983556	2.118036
7.H	-2.894228	-0.103401	2.930699
8.H	-0.731691	2.157432	3.360713
9.C	-1.592978	0.821234	-0.906616
10.H	-2.429244	0.209855	-0.543814
11.H	-1.979431	1.779620	-1.274958
12.H	0.518158	-1.158776	0.239896

13.H	-0.938180	-1.122933	1.270734
14.C	-3.209487	0.890559	2.594808
15.H	-3.752157	1.387542	3.407714
16.H	-3.875674	0.786877	1.731313

TS: CGeAs + H_2
E = −1671.91
H = −1590.48
G = −1620.94
N_{imag} = 1,−1179i cm^{-1}

1.C	-0.015913	-0.743783	1.039976
2.As	-0.166382	1.148473	0.423698
3.H	0.596705	-0.769556	1.947385
4.H	-0.879378	0.266450	-1.864188
5.H	-1.019104	3.029220	2.968159
6.Ge	-1.850815	2.061480	1.997739
7.H	-2.403062	-0.233103	3.155879
8.H	-0.458687	1.946361	3.025858
9.C	-1.436642	0.797594	-1.082800
10.H	-2.291615	0.189692	-0.767998
11.H	-1.787474	1.756856	-1.478895
12.H	0.484117	-1.309975	0.244291
13.H	-0.997092	-1.187332	1.239885
14.C	-2.861929	0.760012	3.148193
15.H	-2.900290	1.158585	4.168343
16.H	-3.879550	0.690898	2.749045

PC: CGeAs + H_2
E = −1715.52
H = −1632.36
G = −1661.31
N_{imag} = 0

1.C	-0.016058	-0.700483	1.048099
2.As	-0.228070	1.166710	0.363356
3.H	0.620147	-0.681059	1.939864
4.H	-1.210715	0.246302	-1.807625
5.H	-2.166242	3.414248	1.747422
6.Ge	-1.669889	2.006357	2.158937
7.H	-2.889949	-0.112139	2.946601
8.H	-0.793097	2.197010	3.420947
9.C	-1.667580	0.792289	-0.973794
10.H	-2.478654	0.193742	-0.545589
11.H	-2.062873	1.744344	-1.344919
12.H	0.483402	-1.284671	0.266202
13.H	-0.980468	-1.160468	1.288422
14.C	-3.224498	0.876052	2.612383
15.H	-3.788702	1.355711	3.421144
16.H	-3.877151	0.761200	1.740034

TS: CSnN + H_2
E = −1720.00
H = −1636.43
G = −1664.79
N_{imag} = 1,−1295i cm^{-1}

1.C	-0.214927	-0.398129	0.974310
2.N	-0.553067	0.972190	0.580721
3.H	0.207498	-0.412813	1.987153
4.H	-0.236631	0.649083	-1.477816
5.H	-0.826160	3.325699	2.580163
6.Sn	-1.788837	2.026081	1.927839
7.H	-2.279338	-0.182194	3.519478

8.H	-0.309005	2.195729	3.211703
9.C	-1.037055	1.004092	-0.805489
10.H	-1.922141	0.362766	-0.988130
11.H	-1.293797	2.030205	-1.099502
12.H	0.545933	-0.797718	0.280427
13.H	-1.071832	-1.100727	0.948119
14.C	-2.844100	0.738915	3.355549
15.H	-2.945209	1.301747	4.288298
16.H	-3.827084	0.521574	2.929821

PC: CSnN + H_2
E = −1766.87
H = −1681.44
G = −1710.25
N_{imag} = 0

1.C	-0.016671	-0.401341	0.826500
2.N	-0.566376	0.900598	0.442609
3.H	0.568810	-0.315120	1.752313
4.H	-0.688936	0.449096	-1.617297
5.H	-1.884699	3.471887	1.282724
6.Sn	-1.517781	1.941735	1.986930
7.H	-3.025720	-0.057402	3.096960
8.H	-0.305441	2.062931	3.206500
9.C	-1.341060	0.794469	-0.795592
10.H	-2.187356	0.079599	-0.733868
11.H	-1.746062	1.774763	-1.082482
12.H	0.660830	-0.769932	0.036019
13.H	-0.787483	-1.184281	0.981051
14.C	-3.296451	0.928429	2.704659
15.H	-3.743225	1.530687	3.503525
16.H	-4.013565	0.818240	1.884483
